# Spliceosome induction is a druggable dependency of RAS-driven senescence and cancer

**DOI:** 10.1038/s41467-026-71564-z

**Published:** 2026-04-15

**Authors:** Verena Wagner, Laura Bousset, Mariana Ascensão-Ferreira, Bin Sun, José Efren Barragan Avila, Alexandre Kaizeler, Rita Martins-Silva, Mohammad Rahbari, Mirian Fernández-Vaquero, Scott Haston, Michele Tinti, Joaquim Pombo, Sanjay Khadayate, Susanne Roth, Christian M. Schürch, Juan Pedro Martínez-Barbera, Anat Bahat, Keng Boon Wee, Jennifer P. Morton, Nisar Malek, Andrew J. Innes, Santiago Vernia, Nuno L. Barbosa-Morais, Suchira Gallage, Mathias Heikenwalder, Jesús Gil

**Affiliations:** 1 MRC Laboratory of Medical Sciences (LMS), London, UK; 2 Institute of Clinical Sciences (ICS), Faculty of Medicine, Imperial College London, London, UK; 3 University of Tübingen, Faculty of Medicine, Institute for Interdisciplinary Research on Cancer Metabolism and Chronic Inflammation, M3 Research Center for Malignome, Metabolome and Microbiome, Tübingen, Germany; 4 Department of Internal Medicine I, University Hospital Tübingen, Tübingen, Germany; 5 Instituto de Medicina Molecular João Lobo Antunes, Faculdade de Medicina, Universidade de Lisboa, Lisbon, Portugal; 6 Faculdade de Medicina, Universidade de Lisboa, Lisbon, Portugal; 7 German Cancer Research Center (DKFZ), Division of Chronic Inflammation and Cancer, Heidelberg, Germany; 8 Gulbenkian Institute for Molecular Medicine, Lisbon, Portugal; 9 Developmental Biology and Cancer Programme, Birth Defects Research Centre, Great Ormond Street Institute of Child Health, University College London, London, UK; 10 Wellcome Centre for Anti-Infectives Research (WCAIR), School of Life Sciences, University of Dundee, Dundee, UK; 11 Department of General, Visceral and Transplantation Surgery, University Hospital Heidelberg, Heidelberg, Germany; 12 Department of Pathology and Neuropathology, University Hospital and Comprehensive Cancer Center Tübingen, Tübingen, Germany; 13 Cluster of Excellence iFIT (EXC 2180) “Image-Guided and Functionally Instructed Tumor Therapies”, University of Tübingen, Tübingen, Germany; 14 Department of Biomolecular Sciences, The Weizmann Institute of Science, Rehovot, Israel; 15 Institute of Molecular and Cell Biology (IMCB), Agency for Science, Technology, and Research (A*STAR), Singapore, Republic of Singapore; 16 Cancer Research UK Scotland Institute, Garscube Estate, Glasgow, UK; 17 School of Cancer Sciences, University of Glasgow, Glasgow, UK; 18 Centre for Haematology, Department of Immunology and Inflammation, Faculty of Medicine, Imperial College London, London, UK; 19 Institute of Biomedicine of Valencia (IBV), CSIC, and Valencia Biomedical Research Foundation, Centro de Investigación Príncipe Felipe (CIPF), Associated Unit to the IBV-CSIC, Valencia, Spain

**Keywords:** Senescence, Targeted therapies, RNA splicing

## Abstract

RAS family proteins, including HRAS, NRAS, and KRAS, are frequently mutated in cancer. Although there has been recent success in designing inhibitors that target oncogenic RAS, they elicit resistance and treating RAS-driven cancer remains difficult. Here, employing a proteomic analysis, we find that multiple spliceosome components are upregulated in the nuclei of cells undergoing RAS-induced senescence. This upregulation depends on RAS signalling and occurs in both senescent preneoplastic and fully transformed cancer cells. Spliceosome components are also highly expressed in preneoplastic and cancerous lesions in human and murine lung, liver, colorectal, and pancreatic cancers. Using siRNA screens, we identify six spliceosome components, including SF3B1 and RBM39, that are essential in cells expressing oncogenic RAS. We find that SF3B1 is required in these cells for maintaining splicing fidelity. By combining transcriptome and splicing analyses with functional screens, we identify the RNA Pol II-associated factor SPT5 as a key mediator of the SF3B1 effects. Importantly, using mouse models of liver cancer, we show that RBM39 and SF3B1 inhibitors are effective in targeting both preneoplastic lesions and aggressive tumours expressing oncogenic RAS. In summary, our study highlights the spliceosome as a promising target for RAS-driven cancers capable of inhibiting both cancer initiation and progression.

## Abstract (web summary)

While there have been recent improvements, RAS-driven cancers remain difficult to treat. Here, the authors identify the spliceosome as a vulnerability in RAS-induced senescent cancer cells and show that therapeutically targeting this is effective in preclinical models of RAS-driven liver cancer.

## Introduction

RAS family proteins are mutated in up to a third of all human cancers^1^. Whereas in normal (non-transformed) cells RAS proteins are transiently activated in response to growth factors, missense mutations in KRAS, NRAS, or HRAS can lead to constitutive activation, which can trigger oncogene-induced senescence (OIS)^2^. OIS is a cellular stress response that occurs in preneoplastic lesions^3^ and restrains tumorigenesis, controlling cancer initiation events^4–6^. However, with continuing oncogenic RAS activity, cells eventually escape senescence and undergo unrestrained proliferation and tumorigenesis by engaging signalling cascades such as the mitogen-activated protein kinase (MAPK) or phosphoinositide 3-kinase (PI3K) pathways^7^. Mutations in KRAS, found in up to 20% of lung cancers, 80%-100% of pancreatic cancers, and 50% of colorectal cancers, are often associated with a poor therapeutic response and prognosis^1^. Despite the recent success with drugs targeting the KRAS^G12C^ allele^8^, or more widely oncogenic RAS^9^, inhibiting RAS remains a challenge due to acquired resistance^10^. Combinatorial treatment strategies inhibiting different components of the RAS pathway are being trialled, but the rewiring of signalling often leads to resistance^11^. Targeting vulnerabilities associated with oncogenic RAS represents an alternative therapeutic approach. Earlier studies have already identified some selective vulnerabilities associated with RAS mutants^12,13^.

RNA splicing is a fundamental step in the expression of most mammalian genes. In addition to removing introns to produce mature mRNAs, splicing influences transcription and translation, controls mRNA quality, and, through alternative splicing, expands proteomic diversity^14^. Accumulating evidence has shown that dysregulated mRNA splicing is present in many tumours^15^, and recurrent mutations of splicing factors such as SF3B1, SRSF2, UAF1, and ZRSR2 are found in different cancers^16^. Comprehensive analyses of the transcriptome have revealed modifications in both constitutive and alternative splicing that are associated with cancer^17^. These modifications can promote tumorigenesis by different mechanisms. For example, tumour-associated splicing isoforms can promote metastasis^18^, alter metabolism^19^, or influence sensitivity to apoptosis^20^. Moreover, certain tumours such as MYC-driven cancers depend on the spliceosome to maintain metabolic growth^21,22^. Splicing has been suggested to be a vulnerability of different tumour types, including pancreatic cancer^23^, lung cancer^24^ and glioblastoma^25^. While there is some evidence that splicing might also be a vulnerability of Ras-expressing cells^23,24^, the rationale and specific vulnerabilities have not been systematically explored.

Studies over the past decade have shown that splicing factors and specific splicing events can be targeted to treat cancer and other diseases^26^. For instance, spliceosome-mutant cancers are sensitive to SF3B1 inhibitors^27^. In the case of spinal muscular atrophy, modulation of splicing with oligonucleotides enables the expression of a gene that complements the disease deficiency^28^. This approach is increasingly being considered a therapeutic option^29^.

Here, we identify vulnerabilities associated with senescence. To this end, we have characterised proteomic changes in the nuclei of cells undergoing RAS-induced senescence and find a global upregulation of spliceosome components. Interestingly, further examination show that this upregulation is associated with oncogenic RAS expression, rather than senescence per se, as it occurs in several different contexts involving RAS activation, including senescent and transformed cancer cells, and different preneoplastic and cancerous lesions, both in mouse models and patient samples. Using genetic screens, we identify six splicing factors, including RBM39 and SF3B1, that constitute specific vulnerabilities in cells expressing oncogenic RAS. We show that SF3B1 is required for maintaining accurate splicing in cells expressing oncogenic RAS, including the splicing of *SUPT5H*, encoding for the RNA Pol II-associated factor SPT5. By combining transcriptome and splicing analyses with functional screens, we show that SPT5 represents an additional vulnerability of oncogenic RAS-expressing cells. Finally, using models of liver cancer, we show that RBM39 and SF3B1 inhibitors are effective in targeting both preneoplastic lesions and aggressive tumours expressing oncogenic RAS.

## Results

### Multiple spliceosome components are upregulated during RAS-induced senescence

**Fig. 1:**
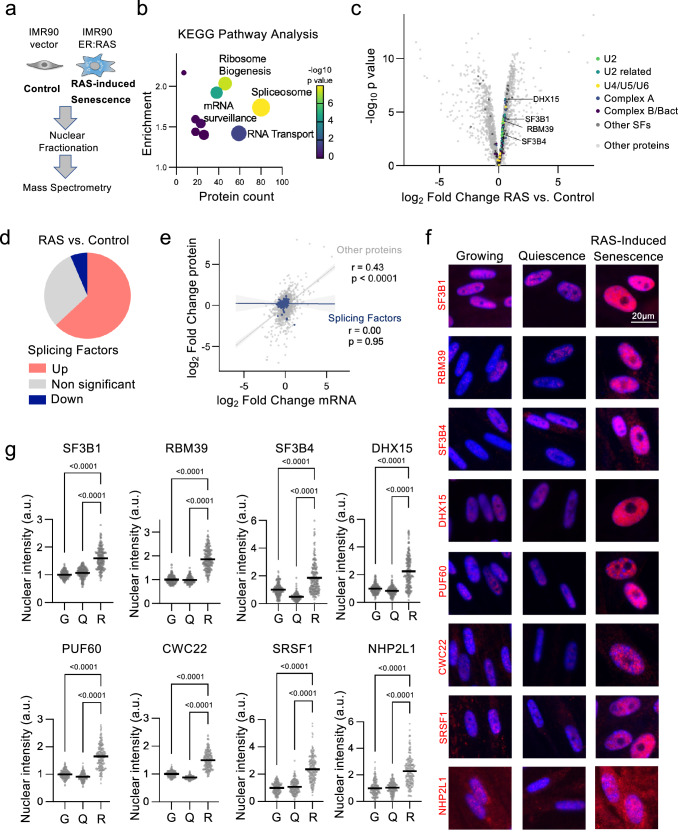
Multiple spliceosome components are upregulated during RAS-induced senescence. **a**–**d** Mass spectrometry of nuclear proteins in cells undergoing RAS-induced senescence. **a** Experimental design. *n* = 5 biological replicates of 4OHT-treated IMR90 ER:RAS cells and *n* = 5 biological replicates of the control cells (IMR90 vector +4OHT). **b** KEGG Pathway Analysis of nuclear proteins in IMR90 cells undergoing RAS-induced senescence. DAVID analysis using all identified nuclear proteins as background (https://david.ncifcrf.gov/summary.jsp, data retrieved December 2021, version 6.8). **c** Volcano plot showing differential levels of nuclear proteins during RAS-induced senescence. Splicing factors (SFs) are coloured as shown in the legend. All other proteins are shown in grey. **d** Percentage of splicing factors that are significantly upregulated, unchanged, or significantly downregulated in the nuclear proteome of IMR90 cells undergoing RAS-induced senescence. **e** Comparison between mRNA and nuclear protein levels in cells IMR90 ER:RAS cells compared with IMR90 vector controls. Splicing factors are represented in blue, and all other proteins are in grey. Simple linear regression, regression curves are represented as plain lines, shaded lines indicate 95% CI (confidence bands). Pearson r and *p* values are indicated. **f**, **g** Expression of different splicing factors in growing (“G”; IMR90 vector 10% FBS + 4OHT), quiescent (“Q”; IMR90 vector 0.1% FBS + 4OHT), or IMR90 cells undergoing RAS-induced senescence (“R”; IMR90 ER: RAS + 4OHT) at day 6 post-induction. **f** Representative images. Scale bar, 20 μm. **g** Plots showing nuclear intensities per cell and mean values, *n* = 200 cells. 1 out of 3 independent experiments. Ordinary one-way ANOVA with Sidak’s multiple comparisons test. Source data are provided as a Source Data file.

As part of our efforts to study senescence, we aimed to identify vulnerabilities associated with cells undergoing oncogene-induced senescence (OIS). OIS, which can be triggered by the activation of oncogenic RAS, occurs early during tumorigenesis and plays critical roles in limiting and influencing tumour progression^30^. We employed this system to characterise proteomic changes associated with OIS, and to identify cellular processes that may represent vulnerabilities. We used IMR90 ER:RAS cells that express a chimeric fusion between oncogenic HRAS^G12V^ and the ligand-binding domain of the estrogen receptor (ER)^31^. Treatment of these cells with 4-hydroxy-tamoxifen (4OHT) activates RAS, triggering senescence^32,33^, while similar treatment has no effect in control IMR90 cells infected with an empty vector (Supplementary Fig. 1). To identify processes relevant to OIS, we treated IMR90 ER:RAS cells with 4OHT for six days, isolated nuclear fractions, and performed mass spectrometry (Fig. 1a and Supplementary Fig. 2a).

Among the bona fide nuclear proteins more highly upregulated in senescent cells, we identified transcription factors such as RELA (known to be induced on senescence^34^) or BHLHE40 (Supplementary Data 1). Ontology analysis of the nuclear proteome revealed strong upregulation of pathways involved in mRNA surveillance, RNA transport, ribosome biogenesis, and the spliceosome (Fig. 1b). Although spliceosome components were not among the proteins showing the highest fold-change in terms of expression relative to controls (Fig. 1c and Supplementary Data 1), we observed a consistent, significant upregulation of multiple splicing proteins, belonging to different complexes, during RAS-induced senescence (Fig. 1d). Moreover, a detailed look into the levels of proteins of one of those complexes (the U2 snRNP complex) showed that several components, in addition to SF3B1, exhibit similar patterns of upregulation (Supplementary Fig. 2b).

Interestingly, while most changes in protein expression during RAS-induced senescence showed a significant correlation with changes in transcription (*p* < 0.001), that was not the case for splicing factors (*p* = 0.95, Fig. 1e), with most of the splicing factors that were upregulated at the protein levels showing no significant changes or downregulation at the mRNA level (Supplementary Fig. 2c). These results suggest that the increase of spliceosome components observed during RAS-induced senescence was regulated post-transcriptionally.

To confirm that multiple spliceosome components were upregulated in the nuclei of cells undergoing RAS-induced senescence and that the effect was due to RAS-induced senescence rather than cell cycle arrest, we carried out quantitative immunofluorescence in growing, quiescent, and senescent (undergoing RAS-induced senescence) IMR90 cells. We took advantage of a panel of antibodies recognising spliceosome components (SF3B1, RBM39, SF3B4, DHX15, PUF60, CWC22, SRSF1, and NHP2L1). Multiple spliceosome components were specifically upregulated in senescent but not in quiescent cells, arguing against the idea that the upregulation was associated with cell cycle arrest (Fig. 1f, g). Treatment of IMR90 vector or IMR90 ER:RAS cells with 4OHT further confirmed that splicing factors are upregulated in nuclei of cells undergoing RAS-induced senescence (Supplementary Fig. 2d–f). To understand that these effects were not cell line-specific, we used BJ human fibroblasts expressing an inducible ER:RAS protein. BJ ER:RAS cells underwent senescence upon treatment with 4OHT and displayed a similar upregulation of splicing factors (Supplementary Fig. 3).

### The global upregulation of spliceosome components is specifically driven by RAS signalling, not senescence induction

Senescence is a heterogeneous response that depends on cell type and inducer^35^. To understand if the global upregulation of splicing factors was a specific response to RAS or a general senescence hallmark, we treated IMR90 cells with several different senescence inducers: the chemotherapeutic drugs etoposide and doxorubicin, the CDK4/6 inhibitor palbociclib, or γ-irradiation (Supplementary Fig. 4). We took advantage of gene set enrichment analysis (GSEA) to confirm senescence induction in the different conditions (Supplementary Fig. 4a). Quantitative immunofluorescence (IF) showed a significant upregulation of the nuclear splicing factors during RAS-induced senescence, but not during other types of senescence (Supplementary Fig. 4b). These results argue against the idea that the global splicing upregulation was a general consequence of senescence.

**Fig. 2:**
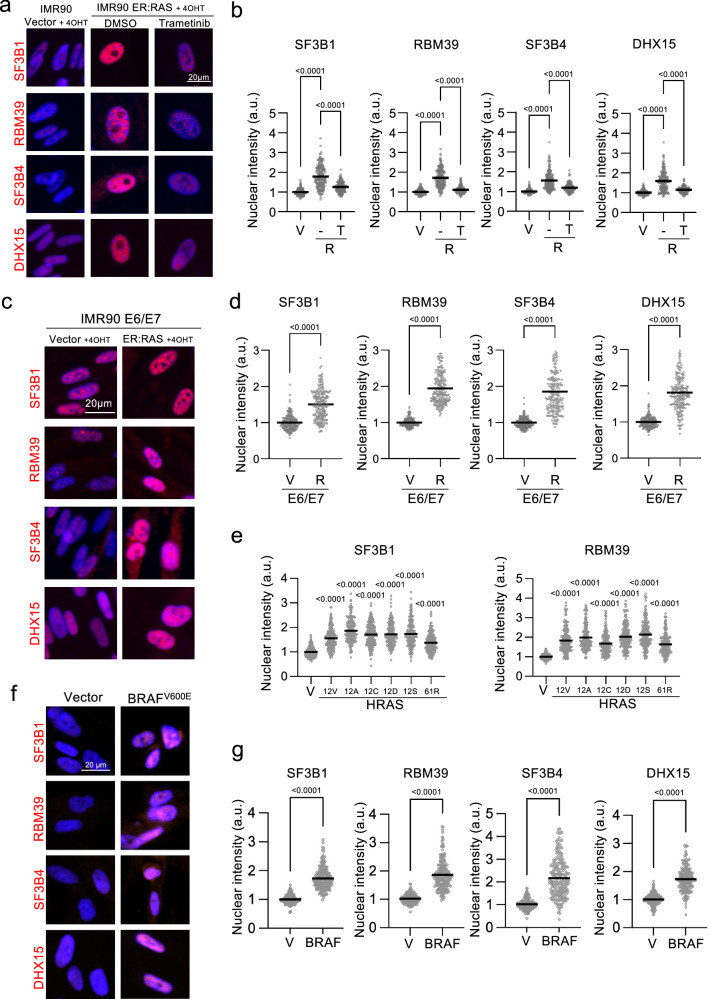
Global upregulation of spliceosome components is associated with oncogenic RAS signalling. **a**, **b** Immunofluorescence staining of different splicing factors in control IMR90 cells (“V”; vector +4OHT) or IMR90 ER:RAS cells undergoing senescence (“RAS”; IMR90 ER:RAS + 4OHT) without (DMSO, -), or with Trametinib treatment (50 nM) from day 4 to day 6 (Trametinib, T). Cells were analysed on day six after induction with 4OHT. **a** Representative images. Scale bar 20 μm. **b** Plots showing single-cell nuclear intensities and mean values. *n* = 200 cells per condition. 1 out of 3 independent experiments. Ordinary one-way ANOVA with Sidak’s multiple comparisons test. **c**, **d** Immunofluorescence staining of different splicing factors (SF3B1, RBM39, SF3B4, and DHX15) in IMR90 E6/E7 cells expressing RAS (“RAS”; IMR90 E6/E7 ER:RAS + 4OHT) and their corresponding controls (“V”; IMR90 E6/E7 vector + 4OHT). **c** Representative images. Scale bar 20 μm. **d** Plots showing single-cell nuclear intensities and mean values, *n* = 200 cells per condition. 1 out of 3 independent experiments. Unpaired t-test, two-tailed. **e** Quantification of the nuclear intensity of splicing factors SF3B1 and RBM39 in IMR90 cells expressing the indicated HRAS mutant or their corresponding vector (V) controls. Plots showing single-cell nuclear intensities and mean values, *n* = 200 cells. 1 out of 3 independent experiments. Ordinary one-way ANOVA with Sidak’s multiple comparisons test. **f**, **g** Immunofluorescence staining of different splicing factors (SF3B1, RBM39, SF3B4, and DHX15) in IMR90 expressing a BRAF^V600E^ (referred to as BRAF) or the corresponding controls (vector, referred to as V). **f** Representative images. Scale bar, 20 μm. **g** Plots showing single-cell nuclear intensities and mean values, *n* = 200 cells per condition. 1 out of 3 independent experiments. Unpaired t-test, two-tailed. Source data are provided as a Source Data file.

To investigate if spliceosome upregulation was linked to RAS signalling rather than to senescence per se, we treated IMR90 ER:RAS cells with trametinib, an inhibitor of MEK, a key downstream effector of RAS and RAF^36^. Trametinib treatment abrogated the upregulation of multiple nuclear spliceosome components (Fig. 2a, b and Supplementary Fig. 5a, b). We next expressed the E6 and E7 oncoviral proteins in IMR90 ER:RAS cells. Expression of E6 and E7, respectively, blocks p53 and Rb signalling, preventing the induction of senescence upon RAS expression^2^. In those conditions, we still observed upregulation of multiple spliceosome components in the nuclei of E6/E7 expressing cells upon RAS activation (Fig. 2c, d and Supplementary Fig. 5c), further supporting the idea that spliceosome upregulation was linked to RAS signalling rather than senescence. Consistent with this conclusion, we observed induction of spliceosome components in IMR90 ER:RAS cells after only 3 days of 4OHT treatment (Supplementary Fig. 5d, e), at a stage where cells hyperproliferate preceding the growth arrest observed upon senescence induction^37^.

The missense G12V mutation is a frequent oncogenic alteration that can impact all RAS family members, but there are many other oncogenic RAS missense mutations^38^. To test whether the upregulation of splicing factors was specifically associated with HRAS^G12V^ or rather a result of any oncogenic activation of RAS, we generated vectors to constitutively express either HRAS^G12V^ or five other HRAS mutations found in tumours (G12A, G12C, G12D, G12S, and G61R). Quantitative IF analysis showed a significant upregulation of nuclear splicing factors in all oncogenic HRAS mutants tested (Fig. 2e and Supplementary Fig. 5f), suggesting that this effect is generally associated with oncogenic RAS activation and is not unique to the G12V mutation. While mutations in RAS family proteins are common in cancer, some tumour types, such as melanoma, instead display frequent mutations in BRAF, a downstream effector of RAS signalling^39^. Interestingly, expression of the oncogenic BRAF^V600E^ allele in IMR90 cells also resulted in an upregulation of several splicing factors (Fig. 2f, g). To confirm that the induction of splicing factors was due to RAS signalling and not related to the proliferation status of the cells, we conducted BrdU incorporation assays. We observed a dissociation between the proliferation status of RAS-expressing cells (e.g., IMR90 ER:RAS cells were arrested at day 6 but not at day 3, while a high percentage of IMR90 E6/E7 ER:RAS cells incorporated BrdU, Supplementary Fig. 5g, h) and the ability to induce splicing factors.

To understand the mechanism behind RAS-mediated upregulation of the splicing factors, we analysed how different inhibitors affected RAS-mediated SF3B1 expression (Supplementary Fig. 6a). While inhibition of p38MAPK or S6K did not affect SF3B1 induction, several mTOR inhibitors (Rapamycin, Temsirolimus and Torin 1) prevented the RAS-mediated SF3B1 induction (Supplementary Fig. 6b, c). Altogether, the above results suggest that the induction of the splicing machinery is not a consequence of senescence or the proliferative status of cells but rather is associated with oncogenic RAS signalling and is related, at least in part, to mTOR-mediated translational control.

### The splicing machinery is expressed at high levels in RAS-driven premalignant and malignant lesions

**Fig. 3:**
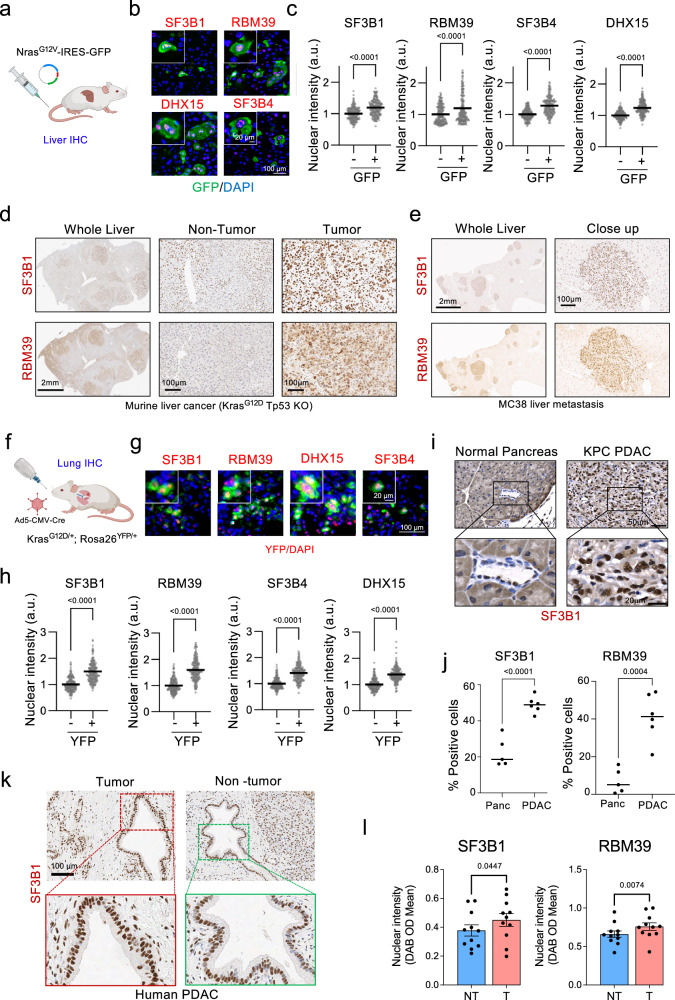
High expression of splicing factors in RAS-driven premalignant and malignant lesions. **a**–**c** Upregulation of splicing factors in NRAS^G12V^-positive hepatocytes. **a** NRAS^G12V^-IRES-GFP was delivered to NSG mouse livers by hydrodynamic tail vein injection. Mice were sacrificed on day nine and livers processed for immunohistochemistry (*n* = 4). Created in BioRender. Wagner, V. (2025) https://BioRender.com/pf423gw. **b** Liver sections co-stained for GFP (green, NRAS^G12V^-positive cells) and SF3B1, RBM39, DHX15, or SF3B4 (red). **c** Quantification of nuclear splicing factors intensities in GFP-negative (-) or positive (+) hepatocytes. Single-cell and mean values, *n* = 200 cells. 1 out of 4 mice. Unpaired t-test, two-tailed. **d** Immunohistochemistry staining of SF3B1 and RBM39 in whole livers, tumour area, and non-neoplastic surrounding parenchyma of *KRAS*^*G12D*^*;Trp53* KO liver cancer model (*n* = 15). Scale bars 2 mm and 100 μm. **e** SF3B1 and RBM39 expression in livers with MC38 (Kras-mutant colorectal cancer) metastasis (*n* = 5). Scale bars 2 mm and 100 μm. **f**–**h** Upregulation of splicing factors in KRAS^G12D^-expressing lung cells. **f** Ad5-CMV-Cre recombinase was delivered to *KRAS*^*G12D/+*^*;Rosa*^*26YFP/+*^ mice by adenoviral inhalation. Mice were sacrificed 8 weeks later (*n* = 3). Created in BioRender. Wagner, V. (2025) https://BioRender.com/2r95qh6. **g** Lung sections co-stained for YFP (green, KRAS^G12D^-harbouring cells) and SF3B1, RBM39, DHX15, or SF3B4 (red). **h** Splicing factors nuclear intensity quantification in YFP negative (-) or positive (+) cells. Single-cell and mean values, *n* = 200 cells per condition. 1 out of 3 mice. Unpaired t-test, two-tailed. **i**, **j** SF3B1 expression in the pancreas (*n* = 5) and pancreatic ductal adenocarcinomas (PDAC) of KPC mice (*LSL-Kras*^*G12D*^*/+; LSL-Trp53*^*R172H/+*^*; Pdx1-Cre*) (*n* = 6). **i** Representative immunohistochemistry images. Scale bars 50 μm and 20 μm. **j** Percentage of SF3B1 or RBM39-positive cells in normal pancreas (Panc) and PDAC. Individual values and mean values. Unpaired t-test, two-tailed. **k**, **l** SF3B1 (*n* = 11) and RBM39 (*n* = 12) expression in human PDAC pancreas sections. **k** Representative SF3B1 staining in tumour (T) and adjacent non-tumour (NT) regions. **l** Quantification of SF3B1 (left) and RBM39 nuclear staining (right). Scale bar, 100 μm. Paired t-test, two-tailed. Source data are provided as a Source Data file.

To investigate whether the RAS-driven upregulation of spliceosome components was also observed in vivo, we first took advantage of a liver model of tumour initiation. In this model, OIS is induced in hepatocytes after hydrodynamic tail vein injection (HDTVI) of a transposon expressing NRAS^G12V^-IRES-GFP^4^ (Fig. 3a). Indeed, immunofluorescence analysis showed that levels of different splicing factors (SF3B1, RBM39, SF3B4, and DHX15) were higher in GFP-positive (NRAS^G12V^-expressing) than GFP-negative hepatocytes (Fig. 3b, c).

We then asked whether the higher levels of expression of splicing components also occurred in later tumour stages. In a model of aggressive liver cancer driven by expression of KRAS^G12D^ and CRISPR-mediated deletion of *Trp53* (*Kras*^G12D^/*Trp53* KO mouse)^40^, SF3B1 and RBM39 expression were consistently higher in liver tumours when compared with adjacent non-tumour regions of the same sections (Fig. 3d). Moreover, analysis of liver sections of mice bearing tumour xenografts derived from MC38 KRAS-mutant colorectal cancer cells^41,42^ also revealed higher expression of spliceosome factors in liver metastases when compared to the surrounding normal liver parenchyma (Fig. 3e and Supplementary Fig. 7a).

Lung adenocarcinoma (LUAD) and pancreatic adenocarcinoma (PDAC) are among the tumour types with a higher frequency of RAS mutations^1^. We investigated whether our observations extended to those tumour types as well. We analysed lung lesions in *Kras*^*G12D/+*^*;ROSA26*^*YFP/+*^ mice, in which KRAS^G12D^ expression had been induced by inhalation of adenoviral particles delivering Cre recombinase to lung cells (Fig. 3f). Mice were sacrificed eight weeks later. Levels of expression of spliceosome components SF3B1, RBM39, SF3B4, and DHX15 in KRAS^G12D^-expressing, YFP-positive cells were significantly higher when compared to surrounding (YFP-negative) lung cells (Fig. 3g, h). Expression of SF3B1 and RBM39 was also elevated in a *Kras*^*G12D*^*/Trp53*^*R172H*^ PDAC model (KPC model: *LSL-Kras*^*G12D/+*^*; LSL-Trp53*^*R172H/+*^*; Pdx1-Cre*)^43–45^ when compared to the normal duct epithelium (Fig. 3I, j and Supplementary Fig. 7b). Importantly, staining of sections from PDAC patients showed higher expression of SF3B1 and RBM39 within the tumors, when compared with adjacent normal tissue, with more than 50% of PDAC cases expressing high levels of SF3B1 and RBM39 (Fig. 3k, l and Supplementary Fig. 7c). Overall, the above results confirm increased expression of multiple splicing components in vivo, in various RAS-driven premalignant and malignant lesions in mouse and human tissue specimens.

### The spliceosome is a vulnerability of cells undergoing RAS-induced senescence

**Fig. 4:**
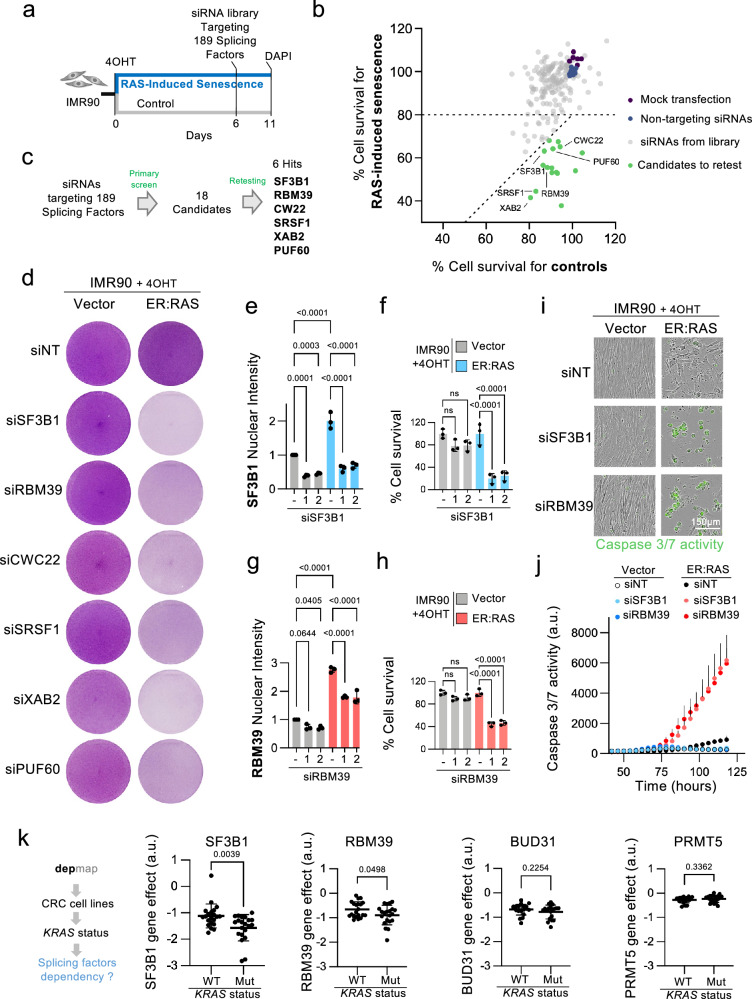
The spliceosome is a vulnerability of cells undergoing RAS-induced senescence. **a**–**c** siRNA screen identifying splicing factors as potential therapeutic targets in RAS-induced senescence. **a** An arrayed siRNA library containing a pool of four siRNAs per gene targeting 189 splicing factors was assembled. IMR90 ER:RAS and IMR90 vector control cells were treated with 4OHT and reverse-transfected with the siRNAs six days post-treatment. Cells were fixed five days after siRNA transfection, and cell numbers were quantified upon DAPI staining. **b** Primary screen results. Hits were selected based on selective killing of RAS-induced senescent cells. siRNAs selected for retesting are marked in green, and identified targets are indicated. **c** Scheme of the primary siRNA screen, retesting, and target selection. **d** Representative crystal violet staining of IMR90 vector or IMR90 ER:RAS cells transfected with siRNAs and fixed five (siRNAs against SF3B1) or six days later. **e**, **f** Knockdown of SF3B1 in RAS-induced senescent IMR90 cells (blue) and their corresponding controls (grey). -, non-targeting siRNA; 1, siSF3B1.1; 2, siSF3B1.2. **e** Quantification of SF3B1 nuclear intensity (immunofluorescence staining two days after siRNA transfection). **f** Cell survival in per cent five days after siRNA transfection. Individual values, mean, and standard deviation. *n* = 3 independent experiments. Ordinary one-way ANOVA with Sidak’s multiple comparisons test. ns, not significant; **g**, **h** Knockdown of RBM39 in IMR90 cells undergoing RAS-induced senescence (blue) and their corresponding controls (grey). -, non-targeting siRNA; 1, siRBM39.1; 2, siRBM39.2. **g** Quantification of RBM39 nuclear intensity (immunofluorescence staining three days after siRNA transfection). **h** Cell survival six days after siRNA transfection in per cent. Individual values, mean, and standard deviation. *n* = 3 independent experiments. Ordinary one-way ANOVA with Sidak’s multiple comparisons test. ns = not significant; Representative pictures (at 96 h post-transfection (**i**) and quantification (**j**) of caspase 3/7 activity. Mean and positive standard deviation. *n* = 3 independent experiments. **k** Data on colorectal carcinoma (CRC) cell lines were retrieved from the Dependency Map database (https://depmap.org/portal/) to analyze splicing factor dependencies based on KRAS status (WT, wild-type; Mut, mutant). *n* = 21 different cell lines. Individual and mean values. Unpaired t test. Source data are provided as a Source Data file.

We reasoned that the increased expression of splicing factors in cells expressing oncogenic RAS may reflect a requirement for increased splicing activity, which may represent a vulnerability of these cells. We, therefore, asked whether the knockdown of specific splicing factors may be selectively detrimental to RAS-expressing cells. To this end, we generated a siRNA library targeting 189 splicing factors and examined how these siRNAs affected the viability of IMR90 cells undergoing RAS-induced senescence, relative to control cells not expressing RAS (Fig. 4a). This screen identified 18 genes whose knockdown selectively killed RAS-expressing cells (Fig. 4b, c). We then performed a secondary screen, testing four independent siRNAs targeting each of the 18 candidates, and confirmed that knockdown of six splicing factors (namely *SF3B1*, *RBM39*, *CWC22*, *SRSF1*, *XAB2*, and *PUF60*) preferentially killed IMR90 cells undergoing RAS-induced senescence (Fig. 4c, d).

The splicing factors whose knockdown produced the strongest synthetic lethal effect in RAS-expressing cells were *SF3B1* and *RBM39*. We confirmed that two independent siRNAs targeting *SF3B1* knocked down its expression (Fig. 4e and Supplementary Fig. 8a) while selectively killing RAS-expressing cells (Fig. 4f). Similarly, siRNAs knocking down *RBM39* preferentially killed RAS-expressing cells (Fig. 4g, h and Supplementary Fig. 8b). The preferential killing of RAS-expressing cells observed upon knockdown of either *SF3B1* or *RBM39* was due to apoptosis, as noticed by the increase in Caspase-3/7 activity that started 3 days after transduction with the siRNAs (Fig. 4I, j). Finally, we also confirmed that two independent siRNAs knocking down *CWC22* (Supplementary Fig. 8c), *SRSF1* (Supplementary Fig. 8d), *PUF60* (Supplementary Fig. 8e), or *XAB2* (Supplementary Fig. 8f) preferentially killed RAS-expressing cells. Interestingly, colorectal carcinoma (CRC) cell lines with hotspot *KRAS* mutation were significantly more sensitive to *SF3B1* or *RBM39* (but not *BUD31* or *PRMT5*) knockout than their corresponding counterparts (Fig. 4k). Moreover, Depmap also unveiled a positive significant correlation between *KRAS* and *SF3B1* and *KRAS* and *RBM39* expression in cancer cells (Supplementary Fig. 9a, b). Previous work has shown that PRMT5 and BUD31 regulate splicing programmes necessary for the survival of MYC-dependent tumours^21,22^. Importantly, CRC-bearing *KRAS* hotspot mutations did not display enhanced sensitivity to *BUD31* or *PRMT5* depletion (Fig. 4k). In addition, MYC expression did not positively correlate with that of *SF3B1* or *RBM39* in cancer cells (Supplementary Fig. 9c, d). Altogether, these results reveal a dependency of cells expressing oncogenic RAS on specific splicing components that is distinct from the dependencies observed in cells expressing oncogenes such as MYC.

### SF3B1 is needed to maintain efficient splicing in cells expressing oncogenic RAS

**Fig. 5:**
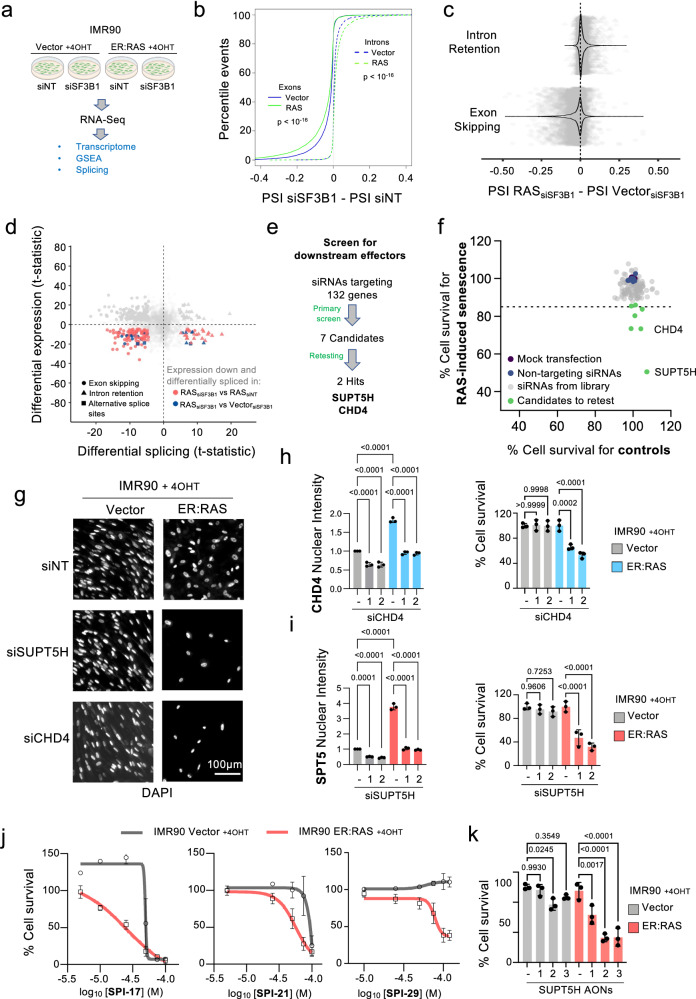
SPT5 is a therapeutic vulnerability of cells expressing oncogenic RAS. **a** RNA-sequencing assessed SF3B1 knockdown in IMR90 cells expressing oncogenic RAS (RAS, IMR90 ER:RAS + 4OHT) versus control (Vector, IMR90 Vector + 4OHT). Cells were reversely transfected with siRNAs against SF3B1 (siSF3B1) or non-targeting (siNT) on day 6 after 4OHT treatment. Created in BioRender. Wagner, V. (2025) https://BioRender.com/ut56gut. **b** Empirical cumulative distributions of ΔPSI (siSF3B1 vs siNT) in RAS (green) and control (blue) for 10,340 “cassette” exons (solid lines) and 5,069 retained introns (dashed lines). **c** SF3B1 knockdown induces more alternative splicing changes in RAS than control cells. ΔPSI of exon skipping and intron retention events in RAS vs vector-expressing IMR90 cells transfected with siSF3B1. **d** Candidate downstream effectors were selected from genes both differentially spliced and downregulated in RAS cells after SF3B1 knockdown. **e**, **f** siRNA screen to identify vulnerabilities underlying SF3B1 dependency in RAS cells. **e** A siRNA library (pools of 4 siRNAs per gene) targeting the 132 genes from **d** was generated and screened as in Fig. 4. Seven primary hits were identified; SUPT5H and CHD4 knockdowns selectively kill RAS-senescent cells. **f** Primary screen results; hits selected for selective killing of RAS-senescent cells. siRNAs selected for retesting are marked in green. **g**–**i** Knockdown of CHD4 (**h**) or SUPT5H (**i**) selectively kill RAS-expressing cells. **g** Representative DAPI staining. CHD4 (**h**) or SPT5 (**i**) nuclear intensity (left) and survival (right) after knockdown with two different siRNAs in RAS and control cells. Individual values, mean, and standard deviation. *n* = 3 independent experiments. Ordinary one-way ANOVA with Sidak’s test. **j** Dose-response of three SPT5 inhibitors in RAS-expressing and control cells. Drugs were added on day six after 4OHT, survival analysed on day 10. Mean and standard deviation, *n* = 3 independent experiments. Nonlinear regression (variable slope, four parameters). **k** Antisense oligonucleotides (AONs) targeting SUPT5H splicing selectively kill RAS-expressing cells. Survival (percentage) measured three days after transfection. Individual values, mean, and standard deviation. *n* = 3 independent experiments. AONs 1-3 target SUPT5H; -, non-targeting AON. Ordinary two-way ANOVA with Sidak’s test. Source data are provided as a Source Data file.

To explore the mechanism underlying this splicing dependency, we focused on SF3B1 and determined the global effect of *SF3B1* knockdown on splicing by using RNA sequencing (RNA-Seq, Fig. 5a). We collected cells at 48 h post-siRNA transfection. At that stage, there is significant SF3B1 knockdown, but the RAS-expressing cells are not yet undergoing apoptosis (Supplementary Fig. 10a). We analysed how *SF3B1* knockdown affected splicing events in cells expressing oncogenic RAS or their control counterparts, taking advantage of RNA-Seq and beta distribution-based analysis of differential alternative splicing^46^(Supplementary Fig. 10b–e). Following the key role of SF3B1 in recognising 3′ splice sites^47^, knockdown of *SF3B1* affected multiple splicing events, causing an increase in intron retention and exon skipping (Fig. 5b and Supplementary Fig. 10b–e) in both conditions. Strikingly, *SF3B1* knockdown induced more splicing alterations in cells expressing oncogenic RAS when compared to their control counterparts [2,296 differentially spliced (ΔPSI ≥ 0.05 and probability of differential splicing *P*_diff_ ≥ 0.80) events in control and 3,658 in oncogenic RAS-expressing cells], suggesting that cells expressing oncogenic RAS have a greater dependency on *SF3B1* for maintaining splicing fidelity. By comparing how *SF3B1* knockdown altered splicing, we observed an increase in exon skipping and intron retention in cells expressing oncogenic RAS (Fig. 5b, c and Supplementary Fig. 10f, g). Furthermore, gene set enrichment analysis (GSEA) suggested that *SF3B1* knockdown upregulated nonsense-mediated decay (NMD), consistent with the idea that the splicing changes lead to an increase in aberrant splicing events (Supplementary Fig. 10h). However, we did not observe a significant increase in the accumulation of poison exons (exons containing a premature stop codon) upon *SF3B1* knockdown in cells expressing oncogenic RAS (Supplementary Fig. 10d, e, g, i, j), suggesting that the upregulation of NMD could be compensatory. This was corroborated by the very low inclusion levels of poison exons in those cells (Supplementary Fig. 10k). Nonetheless, it must be noted that mRNAs including poison exons, like those including frame-disrupting retained introns, are not easily detected by RNA-Seq because of polyA-selection. Sensitivity for NMD-target molecules is therefore very low.

To understand if RBM39 has similar effects, we performed RNA-seq following *RBM39* knockdown in IMR90 cells expressing oncogenic RAS or control (DMSO-treated) cells (Supplementary Fig. 11a). While *RBM39* knockdown led to changes in splicing, including in exon skipping and intron retention (Supplementary Fig. 11b–e), they were less extensive than those upon *SF3B1* knockdown and showed reduced RAS-specific enhancement (Supplementary Fig. 11d, e). Correlation analyses confirmed that SF3B1 and RBM39 regulate distinct sets of splicing events and gene expression changes (Supplementary Fig. 11f, g). Taken together, the above results show that cells expressing oncogenic RAS are demanding towards the splicing machinery and that it is possible to target specific splicing factors, such as SF3B1 or RBM39, to disrupt splicing and reduce the viability of the cells.

### The transcriptional regulator SPT5 is a therapeutic vulnerability of cells expressing oncogenic RAS

Next, we concentrated on investigating why *SF3B1* knockdown constitutes a vulnerability for RAS-expressing cells. A consequence of aberrant splicing is a decrease in mRNA levels due to nonsense-mediated decay (NMD) or alternative mechanisms. We wondered whether any of the transcripts whose splicing was impacted and whose expression decreased by the loss of SF3B1 would also represent vulnerabilities in oncogenic RAS-expressing cells. We, therefore, looked for transcripts that were both differentially/aberrantly spliced in *SF3B1* knockdown cells and showed decreased overall expression. We identified 138 such genes (Fig. 5d), of which 132 were differentially regulated in cells expressing oncogenic RAS (Supplementary Fig. 12a).

We then asked whether depletion of these genes would be lethal in oncogenic RAS-expressing cells. We generated a siRNA library targeting these 132 genes (Fig. 5e) and assessed the effect that their depletion had on the viability of cells expressing oncogenic RAS or their control counterparts (Fig. 5f). The initial screen identified seven candidates whose depletion resulted in the selective death of oncogenic RAS-expressing cells. Upon retesting, using independent siRNAs, we confirmed that two of these genes, *SUPT5H* (the gene encoding SPT5) and *CHD4* (Fig. 5g, i), were essential in RAS-expressing cells.

Analyses of distributions of inclusion of alternatively spliced sequences suggest that *SUPT5H* and *CHD4* are specifically regulated by SF3B1, but not by RBM39 (Supplementary Figs. 11f, g and 12b–d). Splicing of the *SUPT5H* microexon and the *CHD4* intron, differentially spliced concomitantly with their cognate gene downregulation in oncogenic RAS-expressing cells, was markedly altered upon SF3B1 knockdown, whereas no significant changes were observed following RBM39 knockdown. The effect of *SF3B1* knockdown on the splicing of *SUPT5H* and *CHD4* was further confirmed by qRT-PCR using specific primers (Supplementary Fig. 13).

SPT5 is a subunit of the transcription elongation factor DSIF. It stabilises RNA polymerase II and promotes promoter proximal pausing and transcription elongation, therefore playing an important role in the regulation of transcription^48^. CHD4 is a chromatin remodelling enzyme involved in regulating gene repression^49^. Taking advantage of two independent siRNAs each, targeting either *CHD4* (Fig. 5h) or *SUPT5H* (Fig. 5i), we confirmed that the knockdown of these genes resulted in the selective killing of cells expressing oncogenic RAS (Fig. 5g–i). For SPT5, we also used three recently identified SPT5 inhibitors (SPI-17, SPI-21, or SPI-29) that mimic *SUPT5H* knockdown and tested them in concentrations close to their described IC_50_ values^50^. SPT5 inhibitors preferentially kill cells expressing oncogenic RAS (Fig. 5j). Finally, we confirmed that a change in *SUPT5H* splicing could selectively kill oncogenic RAS-expressing cells. To do so, we took advantage of antisense oligonucleotides (AONs) that induce exon skipping by generating steric hindrance^33^. Specifically, we designed three AONs that induced skipping of the differentially regulated exon 5 (microexon HsaEX0062941) of *SUPT5H*. We confirmed that the AONs induced exon skipping and resulted in reduced overall levels of *SUPT5H* mRNA (Supplementary Fig. 14a, b). Importantly, transduction of the AONs targeting *SUPT5H* reduced the viability of IMR90 cells expressing oncogenic RAS but not that of their control counterparts (Fig. 5k). Similar results were observed with AONs targeting *CDH4* (Supplementary Fig. 14c–e).

We wondered whether NMD contributed to explaining the decreased expression of SPT5 and CDH4. To investigate this, we knocked down *SMG1*, a gene involved in NMD. Taking advantage of a vector (HBB PTC39) that expresses a β-globin-PTC39 NMD reporter, we observed that SMG1 results in higher expression of the reporter, consistent with partial NMD inhibition (Supplementary Fig. 15a, b). Partial inhibition of NMD caused by the knockdown of *SMG1* increased *SUPT5H* (Supplementary Fig. 15e) and *CHD4* (Supplementary Fig. 15e) levels. Taken together, these results are consistent with the idea that aberrant splicing of specific transcripts, such as *CHD4* and *SUPT5H*, may contribute to the sensitivity of cells expressing oncogenic RAS to splicing inhibition.

### Cells expressing oncogenic RAS show elevated SPT5 expression along with increased levels of transcription

**Fig. 6:**
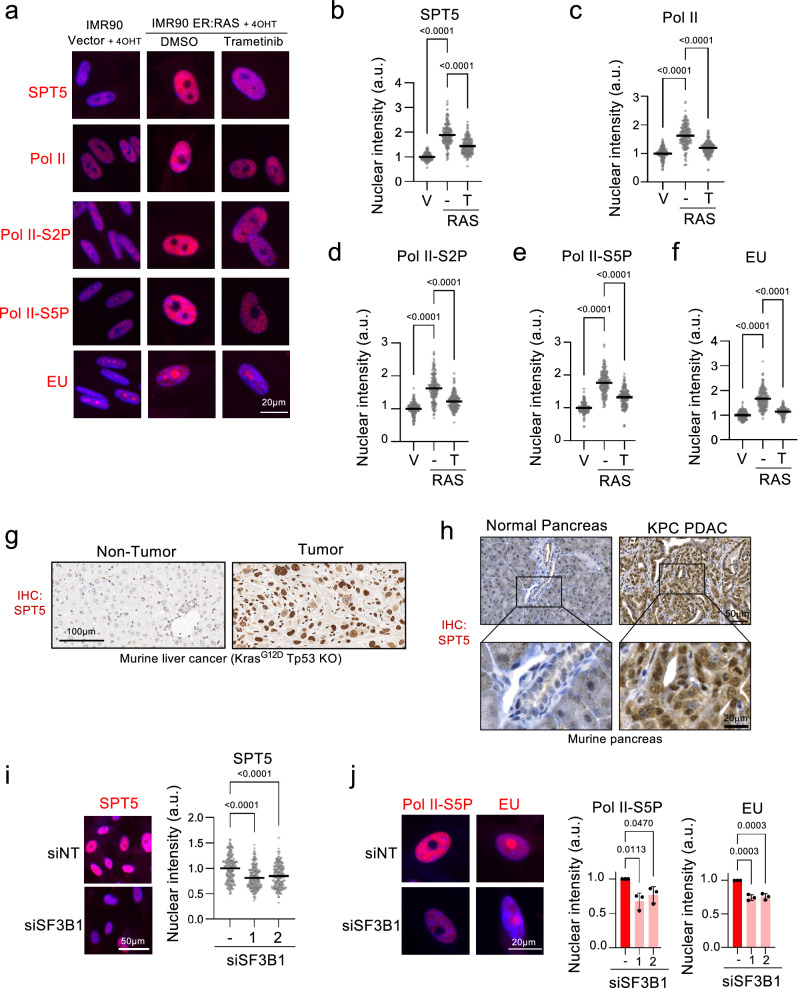
Cells expressing oncogenic RAS show elevated SPT5 expression and increased levels of transcription. **a**–**f** Increased RNA Polymerase II (Pol II)-mediated transcription in cells expressing oncogenic RAS. Cells expressing oncogenic RAS (IMR90 ER:RAS + 4OHT, referred to as RAS) or their control counterparts (IMR90 vector +4OHT, referred to as V) were treated with 4OHT. Trametinib (T) or DMSO (-) was added on day four. Cells were fixed on day six, and immunofluorescence staining was conducted. To assess newly synthesised RNA, 5-ethynyl uridine (EU) incorporation was measured by click chemistry staining. **a** Representative images. Scale bar, 20 μm. Quantification of SPT5 (**b**), total levels of RNA polymerase II (Pol II, **c**), RNA polymerase II phosphorylated at S2 of the carboxy-terminal domain (CTD; Pol II-S2P, **d**), RNA polymerase II phosphorylated at S5 of the CTD (Pol II-S5P, **e**), and newly synthesised RNA/incorporated EU (**f)**. Single-cell nuclear intensities and mean values, *n* = 200 cells. 1 out of 3 independent experiments. Ordinary one-way ANOVA with Sidak’s multiple comparisons test. Immunohistochemistry staining of SPT5 in mouse models of KRAS-dependent liver cancer (*Kras*^*G12D*^*;Trp53* KO model, *n* = 15, **g**), and pancreatic cancer (*KPC: LSL-Kras*^*G12D*^*/+; LSL-Trp53*^*R172H/+*^*; Pdx1-Cre*. *n* = 6, **h**) compared to surrounding normal liver (*n* = 15) or pancreatic (*n* = 5) parenchyma, respectively. Representative images. **i**, **j** Knockdown of *SF3B1* results in reduced *SUPT5H* expression and decreased transcription. IMR90 cells expressing oncogenic RAS (IMR90 ER:RAS cells) were transfected with siRNAs targeting *SF3B1* (1, siSF3B1.1; 2, siSF3B1.2) or non-targeting controls (-, siNT) six days after induction with 4OHT. Levels of SPT5 (**i**), Pol II-S5P (**j**, left), and EU as a surrogate of transcription (**j**, right) were measured. **i** Representative pictures (scale bar 50 μm) and quantification of single cell nuclear intensities and mean values, *n* = 200 cells. 1 out of 3 independent experiments. Ordinary one-way ANOVA with Dunnett’s multiple comparisons test. **j** Representative images (scale bar 20 μm) and quantification of EU and Pol II CTD phospho S5 nuclear intensities. Individual values, mean, and standard deviation. *n* = 3 independent experiments. Ordinary one-way ANOVA with Dunnett’s multiple comparisons test. Source data are provided as a Source Data file.

To gain insights into the link between oncogenic RAS expression and vulnerability to loss of SPT5, we examined the relation between oncogenic RAS, SPT5, and transcriptional regulation. Quantitative immunofluorescence analysis (Fig. 6a, b) showed that nuclear SPT5 levels increased upon expression of oncogenic RAS, in a manner dependent on MEK signalling. The increase in SPT5 levels correlated with higher levels of total RNA Pol II (Fig. 6a, second panels from top and Fig. 6c) as well as an increase in RNA Pol II phosphorylation on serine 2 and serine 5, which is associated with active transcription (Fig. 6a, d, e). Thus, cells expressing oncogenic RAS also express higher levels of SPT5 and exhibit increased active transcription. To directly measure levels of RNA synthesis, we pulsed cells with the uridine analogue 5-ethynyl-uridine (EU), which is incorporated into nascent RNA and can be visualised using click chemistry^51^. Consistent with the above results, we found a pan-nuclear increase in EU-signal in cells expressing oncogenic RAS (Fig. 6a, f), suggesting elevated RNA Pol II-dependent transcription in RAS-expressing cells.

To confirm that the increase in SPT5 levels was due to oncogenic RAS expression, we examined a few other cellular contexts. We observed an increase in SPT5 levels and elevated RNA Pol II-dependent transcription in cells expressing oncogenic RAS, even if the senescence pathways were disabled (Supplementary Fig. 16a–f).

Moreover, analysis of a pan-cancer dataset using Depmap (https://depmap.org/portal/) unveiled positive correlations between *KRAS* (but not *MYC*) and *SUPT5H* expression in cancer cells (Supplementary Fig. 16g, h). Significantly, there was also a positive correlation between the dependency of cancer cell lines on *SF3B1* and *SUPT5H* (Supplementary Fig. 16i). Furthermore, analysis of liver (Fig. 6g) and pancreatic (Fig. 6h and Supplementary Fig. 16j) murine tumours driven by oncogenic KRAS also showed elevated levels of SPT5 in aberrant hepatocytes or pancreatic cells, respectively. Finally, to understand how SF3B1 might affect SPT5 levels, RNA Pol II, and transcriptional regulation, we knocked down *SF3B1*. SF3B1 depletion (Fig. 6i) or its inhibition using Pladienolide B (Supplementary Fig. 16k) resulted in reduced SPT5 protein levels. Moreover, we also observed a decrease in levels of RNA Pol II phosphorylated at serine 5 and in nascent RNAs upon *SF3B1* knockdown (Fig. 6j). These results reveal a link between SF3B1-dependent splicing regulation and transcriptional regulation in oncogenic RAS-expressing cells and are consistent with the idea that SPT5 may mediate, at least in part, the effects of SF3B1 on mRNA transcription in these cells^52^.

### SF3B1 inhibition targets premalignant lesions driven by oncogenic RAS

**Fig. 7:**
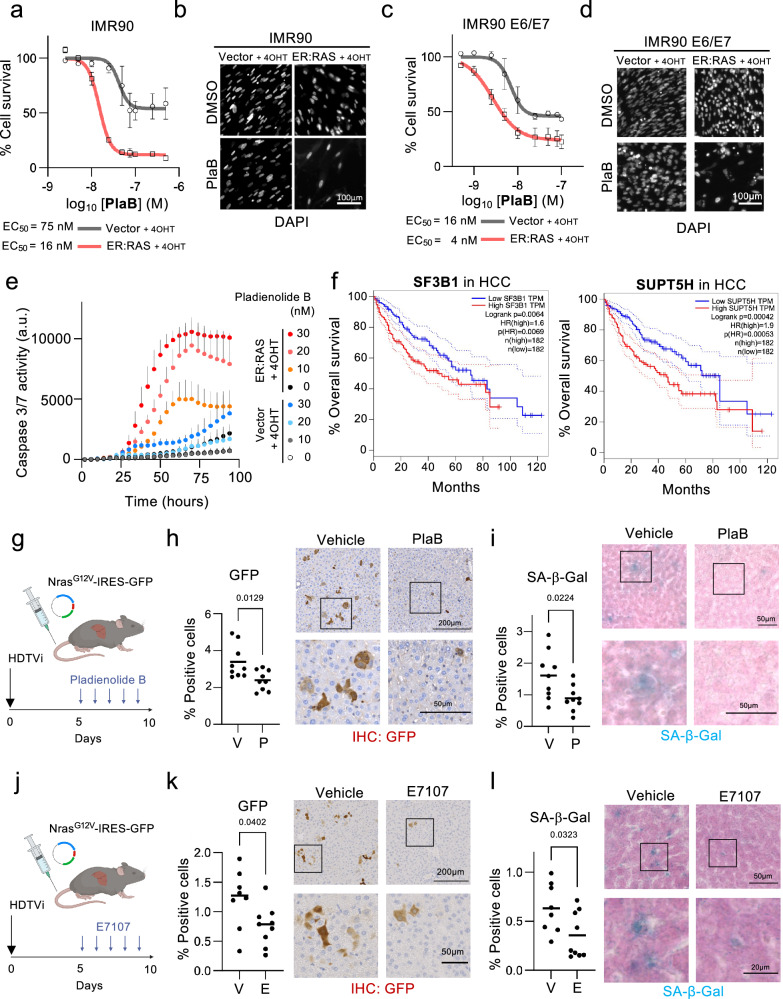
SF3B1 inhibition targets oncogenic RAS-driven preneoplastic lesions. Dose-response analysis of Pladienolide B (PlaB) (**a**–**c**) on survival of IMR90 cells undergoing RAS-induced senescence and their corresponding controls (IMR90 ER:RAS and IMR90 vector cells) (**a**, **b**) and of IMR90 E6/E7 cells expressing oncogenic RAS and respective control cells (IMR90 E6/E7 ER:RAS and IMR90 E6/E7 vector) (**c**, **d**). PlaB was added six days after 4OHT induction for IMR90 ER:RAS and vector cells (**a**, **b**), after three days for IMR90 E6/E7 ER:RAS and vector cells (**c**, **d**).Cells were analysed four days later. Mean and standard deviation, *n* = 3 independent experiments. **b**–**d** Representative images of DAPI-stained cells. Scale bar, 100 μm. **e** Quantification of Caspase 3/7 activity. Mean values and positive standard deviation. *n* = 3 independent experiments. **f** Survival curves for hepatocellular carcinoma (HCC) cases stratified by high (red; cutoff-high 50%) or low (blue; cutoff-low 50%) expression of *SF3B1* (left) and *SUPT5H* (right). Curves were generated with Gene Expression Profiling Interactive Analysis (GEPIA). **g**–**l** A transposon expressing oncogenic NRAS^G12V^ and GFP (NRAS^G12V^-IRES-GFP) was delivered to 8-week-old female C57BL/6 mice via hydrodynamic tail vein injection (HTVI). **g** PlaB (10 mg/kg) was injected intraperitoneally daily from day five to day nine after HTVI. Mice were sacrificed on day ten. Created in BioRender. Wagner, V. (2025) https://BioRender.com/7hsoeyo. GFP immunohistochemistry staining (scale bars 200 and 50 μm) (**h**) and SA-β-galactosidase assay (scale bars 50 and 20 μm) (**i**). Quantifications (left) and representative images (right). Vehicle (V, *n* = 9 mice), Pladienolide B (PlaB/P, *n* = 9 mice). Individual and mean values. Unpaired t-test, two-tailed. **j** E7107 (2 mg/kg) was injected intraperitoneally daily from day five to day nine after HTVI. Mice were sacrificed on day ten. Created in BioRender. Wagner, V. (2025) https://BioRender.com/7hsoeyo. GFP immunohistochemistry staining (scale bars 200 and 50 μm) (**k**) and SA-β-galactosidase assay (**l**). Quantifications (left) and representative images (scale bars 50 and 20 μm) (right). Vehicle (indicated by V, *n* = 8 mice), E7107 (indicated by E, *n* = 9 mice). Individual values and means. Unpaired t-test, two-tailed. Source data are provided as a Source Data file.

Next, we investigated whether the SF3B1 dependency observed in cells expressing oncogenic RAS could be exploited therapeutically. We first confirmed that, as with *SF3B1* knockdown, the SF3B1 inhibitor Pladienolide B (PlaB)^53,54^ also selectively kills IMR90 cells expressing oncogenic RAS (Fig. 7a, b and Supplementary Fig. 17a), irrespective of whether senescence was engaged or not (Fig. 7c, d). PlaB induced apoptosis in RAS-expressing cells, as suggested by the selective increase of caspase 3/7 activity (Fig. 7e) and the increased survival in the presence of inhibitors of apoptosis (but not of ferroptosis, necroptosis or pyroptosis, Supplementary Fig. 17b). PlaB did not selectively kill cells undergoing senescence induced by doxorubicin, etoposide, palbociclib or γ-irradiation (Supplementary Fig. 17c), in agreement with the lack of SF3B1 upregulation on those conditions (Supplementary Fig. 4b). Moreover, PlaB selectively killed BJ cells expressing oncogenic RAS (Supplementary Fig. 17d), but not cells expressing activated AKT (myrAKT) or with reduced levels of the tumour suppressor p16 or p53 (Supplementary Fig. 17e, f). Moreover, taking advantage of data derived from The Cancer Genome Atlas (TCGA), we observed that hepatocellular carcinoma (HCC) patients with high levels of either *SF3B1* or *SUPT5H* displayed significantly worse survival than their low-expressing counterparts (Fig. 7f).

Given this, we then asked whether SF3B1 inhibition could eliminate cells expressing oncogenic RAS in vivo. To do so, we exploited a model of liver cancer initiation in mice, in which senescence is induced in preneoplastic hepatocytes upon hydrodynamic tail vein injection (HDTVI) of a transposon plasmid expressing oncogenic RAS (NRAS^G12V^-IRES-GFP)^4^. As shown above, SF3B1 and RBM39 expression are upregulated in preneoplastic hepatocytes in this model (Fig. 3a–c). We treated mice with the SF3B1 inhibitors E7107 or PlaB^53^ for five consecutive days, starting five days after injection (Fig. 7g–l). Treatment with either SF3B1 inhibitor resulted in a significant reduction of NRAS^G12V^-positive hepatocytes (as suggested by GFP staining, Fig. 7h, k) and senescence (Fig. 7I, l).

### RBM39 inhibition targets premalignant lesions driven by oncogenic RAS

**Fig. 8:**
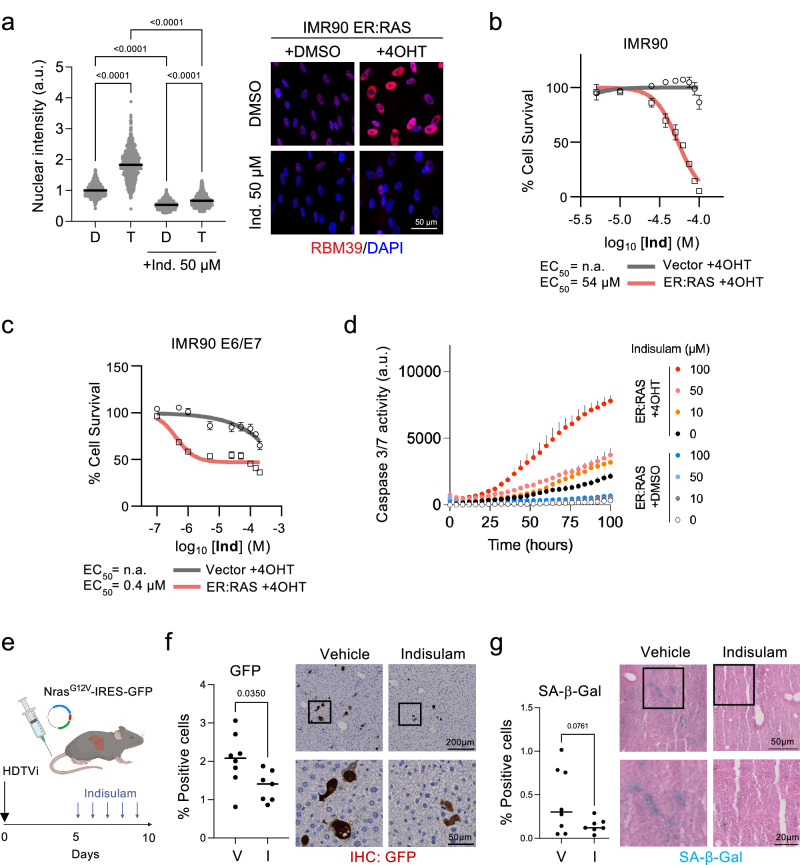
The RBM39 degrader indisulam targets oncogenic RAS-driven preneoplastic lesions. **a** Quantification (left) and representative images (right) of RBM39 immunostaining of IMR90 ER:RAS induced with 4OHT (T) or DMSO (D) for seven days, then treated with 50 μM of indisulam (Ind) for twenty-four hours. Mean and individual values, *n* = 1899 (D), 751(T), 1446 (D + indisulam) and 763 (T + indisulam) cells. 1 out of 3 independent experiments. Ordinary one-way ANOVA with Tukey’s multiple comparisons test. Scale bar, 50 μm. Dose-response curves of empty vector-expressing IMR90 ( + 4OHT, control) and RAS-expressing IMR90 ER:RAS ( + 4OHT, RAS) (**b**) and E6/E7 oncoproteins-expressing IMR90 control ( + 4OHT, E6/E7) and E6/E7 oncoproteins-expressing IMR90 RAS ( + 4OHT, E6/E7 RAS) (**c**) treated with indisulam (Ind). Mean and standard deviation, *n* = 3 independent experiments. **d** Quantification of Caspase 3/7 activity measured using the CellEvent^TM^ Caspase-3/7 Green Detection Reagent and Incucyte live cell imaging after treatment with increasing doses of Indisulam. Plots show mean values and positive standard deviation. Mean and standard deviation, *n* = 3 independent experiments. **e** Experimental design. A transposon expressing oncogenic NRAS^G12V^ and GFP (NRAS^G12V^-IRES-GFP) was delivered to 8-week-old female C57BL/6 mice via hydrodynamic tail vein injection (HTVI). Indisulam (25 mg/kg) was injected intraperitoneally daily from day five to day nine after HTVI. Mice were sacrificed on day ten. Created in BioRender. Wagner, V. (2025) https://BioRender.com/7hsoeyo. GFP IHC staining (**f**) and SA-β-galactosidase assay (**g**). Quantifications (left) and representative images (right, scale bars as indicated). Vehicle (V, *n* = 8 mice), Indisulam (I, *n* = 7 mice). Individual and mean values are plotted. Unpaired t-test, two-tailed. Source data are provided as a Source Data file.

We also investigated whether RBM39 inhibition using indisulam could be exploited therapeutically to target RAS-expressing cells. Indisulam is a molecular glue that targets RBM39 for degradation^55,56^. We confirmed that indisulam treatment ablated RBM39 in IMR90 ER:RAS cells (Fig. 8a). Moreover, indisulam selectively killed IMR90 cells expressing oncogenic RAS (Fig. 8b, c), irrespective of whether senescence was engaged or not, by inducing apoptosis (Fig. 8d). Indisulam selectively killed BJ expressing oncogenic RAS (Supplementary Fig. 18a), but not cells expressing activated AKT (myrAKT) or with reduced levels of the tumour suppressor p16 or p53 (Supplementary Fig. 18b, c). We took advantage of the model of RAS-induced liver preneoplasias and treated mice with indisulam for five consecutive days, starting five days after injection (Fig. 8e). Indisulam treatment significantly reduced NRAS^G12V^-positive hepatocytes (as suggested by GFP staining, Fig. 8f) and showed a non-significant reduction of senescent cells (Fig. 8g).

### The SF3B1 inhibitor E7107 targets aggressive liver tumours driven by oncogenic RAS

**Fig. 9:**
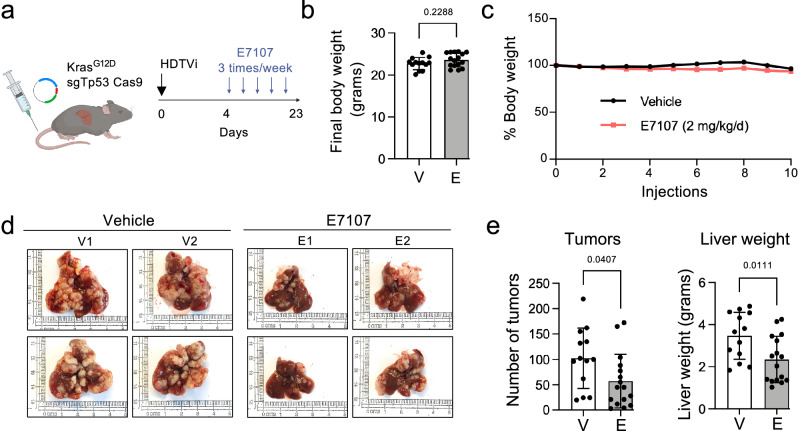
The SF3B1 inhibitor E7107 targets aggressive tumours driven by oncogenic RAS. **a** Experimental design. Plasmids expressing *Kras*^*G12D*^, *sgTrp53*, and *Cas9* were delivered to the livers of 8-week-old male C57BL/6 mice by HTVI. Treatment with E7107 (2 mg/kg, indicated as E) or vehicle (V) started on day four. Mice received intraperitoneal injections three times per week (10 injections in total), and all animals were sacrificed on day 23. Created in BioRender. Wagner, V. (2025) https://BioRender.com/7hsoeyo. **b**, **c** SF3B1 inhibitor E7107 does not cause loss of body weight in mice. Final body weight (grams) and % body weight (relative to initial weight). Individual, mean values and standard deviation of vehicle-treated (indicated by V, *n* = 15) and E7107-treated (E, *n* = 16) mice are plotted in (**b**). Mean values are plotted in (**c**). Unpaired t-test, two-tailed. ns, not significant. **d** Representative images of the livers of two mice treated with vehicle (V1 and V2) or E7107 (E1 and E2). **e** Tumour numbers and liver weight in mice treated with vehicle (*n* = 13) or E7107 (*n* = 16). Individual values and means with standard deviation are plotted. Unpaired t-test, two-tailed. Source data are provided as a Source Data file.

Finally, we took advantage of a mouse model of aggressive liver cancer (*KRAS*^*G12D*^*;Trp53* KO)^40^, which also displays high levels of SF3B1 (Fig. 3d). For these experiments, we chose to use the SF3B1 inhibitor E7107, which has greater stability compared to PlaB and has been used more widely in pre-clinical models^57^ and humans^58^. We treated the mice with E7107 three times a week (Fig. 9a) and confirmed that the treatment regimen was well tolerated, e.g., weight was unaffected by the treatment (Fig. 9b, c). As previously reported in refs. ^40,59^, mice developed aggressive multifocal liver tumours about three weeks after injection (Fig. 9d). Importantly, mice treated with E7107 displayed a significantly lower liver weight relative to controls and a significant reduction in tumour numbers (Fig. 9d, e). Taken together, these results demonstrate that SF3B1 and RBM39 inhibitors are effective therapeutics for the treatment of RAS-driven lesions.

## Discussion

Despite being altered in up to a third of human cancers, RAS family proteins have been considered undruggable until recently. Novel approaches, highlighted by the success of covalent inhibitors against the KRAS^G12C^ allele^11^ and the development of inhibitors with broader specificity towards oncogenic RAS^9^, are opening new possibilities. However, rapidly acquired resistance remains an untackled challenge^10,60^. Thus, it is vital to pursue alternative, complementary approaches to treat RAS-driven tumours, including combinatorial treatments^61,62^ and targeting RAS-specific vulnerabilities^12,13^.

In this study, we employed nuclear proteomics to reveal that cells expressing oncogenic RAS display high levels of multiple spliceosome components in their nuclei. Furthermore, high expression of splicing factors is observed in both preneoplastic and malignant lesions. Specifically, cells expressing oncogenic RAS depend on several splicing factors for viability, including SF3B1 and RBM39. These spliceosome components, therefore, represent vulnerabilities that can be exploited to treat RAS-driven malignancies. Missense mutations lock RAS family proteins into a constitutively active state. Constitutively active RAS drives cellular transformation by activating signalling pathways that promote proliferation, shift metabolism towards aerobic glycolysis, and alter the tumour microenvironment via the secretion of chemokines and cytokines^63^. To be able to implement this tumorigenic program, cells need to modify their transcriptome, which in turn requires active and efficient transcriptional and splicing machinery. This may explain why efficient splicing is a requirement and a vulnerability of cells expressing oncogenic RAS.

A similar dependency on the spliceosome has been shown in MYC-dependent tumours^21,22^. As with oncogenic RAS, MYC expression induces profound phenotypic alterations that depend on changes in transcription^64,65^. Therefore, reliance and dependency on core pathways (metabolism, transcription, splicing) is perhaps not a peculiarity of RAS-driven tumours but a more general characteristic of the highly proliferative state associated with oncogenic transformation. However, while MYC- and RAS-driven tumours have a similar dependency on the spliceosome, the mechanisms underlying this dependency appear to diverge. Spliceosome upregulation in MYC-driven tumours occurs at the transcriptional level^21^, whereas here we show that RAS induces a post-transcriptional accumulation of multiple spliceosome components in the nuclei. Furthermore, although there might be some overlaps (e.g., reliance on SF3B1 for both MYC- and RAS-driven tumours^21^ and this study), the specific splicing vulnerabilities appear to be unique to each tumour type. BUD31 and PRMT5 are the main spliceosome vulnerabilities identified in MYC-driven tumours^21,22^. Our splicing-focused RNAi screen identified SF3B1, RBM39, CWC22, SRSF1, XAB2, and PUF60 as key spliceosome vulnerabilities of RAS-driven tumours, providing a broader picture. Overall, it is tempting to speculate that oncogene-driven cancer may have a common reliance on maintaining efficient splicing, but that specific vulnerabilities might be unique to each tumour type.

Expression of oncogenic RAS causes a senescence-like response^2^ that limits tumorigenesis^4–6^ and can be observed in preneoplastic lesions^3^. Although RAS-induced senescence initially hampers tumorigenesis, the persistence of senescent cells in the tumour microenvironment eventually contributes to tumour progression. Indeed, the elimination of senescent cells reduces both the number of PanIN lesions and the incidence of PDAC in a KRAS-driven model of pancreatic cancer^66^. Importantly, the spliceosome is upregulated not only in RAS-driven tumours but also in cells in which RAS expression causes senescence. Interfering with splicing targets both populations of RAS-expressing cells (senescent and transformed) and therefore has a multifactorial effect on tumour growth by targeting not only the tumour cells themselves but also preneoplastic senescent cells that can fuel tumour progression.

To understand why splicing inhibition is a vulnerability of cells expressing oncogenic RAS, we knocked down *SF3B1* and observed that splicing patterns are significantly more impacted in cells expressing oncogenic RAS compared to controls. These cells show significantly more intron retention, more aberrant splicing, and upregulation of NMD. To further explore the consequences of the aberrant splicing, we conducted an RNAi screen targeting genes showing both changes in splicing and expression. Those experiments identified two genes, the transcriptional regulator *CHD4* and the RNA Pol II-associated factor *SUPT5H (*the gene encoding SPT5), as potential mediators that may contribute to the dependency of oncogenic RAS-expressing cells on splicing. Consistent with this idea, SPT5 levels increase in cells expressing oncogenic RAS. We found the link to SPT5 particularly interesting given previous work showing that SF3B1 inhibition interferes with RNA Pol II-dependent transcription^52^. Since SPT5 coordinates multiple functions of RNA Pol II^48^, the effects on SPT5 might explain how interfering with splicing impinges on transcription. Interestingly, MYC recruits SPT5 to RNA Polymerase II to facilitate elongation^67^. SPT5 may therefore be a common vulnerability of both MYC- and RAS-driven tumours. Our results suggest that SPT5 and CDH4 levels might be lower, at least in part, as a result of NMD. However, the alternative splicing events of interest are unlikely to be the culprits, as they are frame-preserving. With the drop in splicing efficiency induced by knocking down *SF3B1*, several frame-disrupting introns likely get retained in both genes, triggering NMD. Such events would not be easily detected by RNA-Seq because transcripts are polyA-selected and, therefore sensitivity for NMD target molecules is very low.

Over the last decade, our understanding of the role of splicing dysregulation in cancer has increased substantially^15^. Importantly, based on work showing mutations in spliceosome components in cancer^27^, compounds targeting SF3B1 and other splicing factors^26^, as well as RNA-based molecules modifying specific splicing events, are currently under development^68^. Our work provides a rationale for extending the use of these inhibitors in RAS-driven cancers. Our study identifies six splicing factors as potential therapeutic targets for eliminating RAS-dependent cancer cells. Inhibitors for two of these factors, SF3B1 and RBM39, already exist. In particular, SF3B1 inhibitors are in clinical trials for haematological malignancies^69^. Moreover, inhibitors of SPT5 are also in development^50^. Overall, we show that co-transcriptional splicing is a hitherto unknown Achilles heel of RAS-driven lesions and that several components involved in splicing and transcription, such as SF3B1, RBM39, and SPT5, are promising targets in RAS-dependent cancers.

## Methods

### Ethics

This research complied with all relevant ethical regulations and was approved and overseen by the following ethics review boards. Pancreatic cancer tissue specimens were obtained from patients who underwent pancreatic resection at the Department of Surgery at Heidelberg University Hospital and provided prior written informed consent under a research protocol approved by the Medical Ethics Committee of Heidelberg University (301/2001 and 159/2002, amendment of May 8^th^, 2012). Liver tumorigenesis experiments and MC38 liver metastasis experiments were performed following German law and with approval from the Regierungspräsidium Karlsruhe (G139/19 and G221/19). All other mouse procedures were performed under license, according to the UK Home Office Animals (Scientific Procedures) Act 1986, ARRIVE 2.0., and local institutional guidelines. The lung cancer experiments were approved by the UCL ethical review committee. Liver cancer initiation experiments were approved by the animal welfare and ethical review board at Imperial College London. Pancreatic cancer experiments were approved by the University of Glasgow’s animal welfare and ethical review board.

### Antibodies

The following antibodies were used in this study: Rabbit polyclonal anti-SF3B1 (Thermo Fisher Scientific, PA5-19679, lot: VL3135364) 1:500-1:1000; Rabbit monoclonal [EPR11986] anti-SF3B1 (Abcam ab172634, lot: 1003548) 1:1000-1:5000; Rabbit polyclonal anti-HCC1 (RBM39) (Thermo Fisher Scientific, PA5-51606, lot: 000018860), 1:100-1:200; Rabbit monoclonal [2D2C8] anti-HCC1 (RBM39) (ProteinTech, 67420-1-Ig, lot:10022276) 1:1000; Rabbit polyclonal anti-HCC1 (RBM39) (Abcam, ab244254, lot 1037608-1) 1:2000; Rabbit polyclonal anti-SF3B4 (Proteintech, 10482-1-AP, lot: 00053884) 1:200; Rabbit polyclonal anti-DHX15 (Atlas Antibodies, HPA047047, lot: 000043569) 1:50-1:200; Rabbit polyclonal anti-PUF60 (Thermo Fisher Scientific, PA5-21411, lot: VA2931357) 1:200; Rabbit polyclonal anti-CWC22 (Thermo Fisher Scientific, PA5-57796, lot: A96309) 1:200; Mouse monoclonal anti-SRSF1 (clone: 103) (Thermo Fisher Scientific, 32-4600, lot: TJ275392) 1:100; Rabbit polyclonal anti-NHP2L1 (Thermo Fisher Scientific, PA5-22010, lot: TH2624110) 1:500; Rabbit polyclonal anti-RNA polymerase II CTD repeat YSPTSPS (Abcam, ab26721, lot: GR3305785-3) 1:1000; Rabbit polyclonal anti-RNA polymerase II CTD repeat YSPTSPS (phospho S2) (Abcam, ab5095, lot: GR3386086-1) 1:1000; Recombinant rabbit monoclonal [EPR19015] anti-RNA polymerase II CTD repeat YSPTSPS (phospho S5) (Abcam, ab193467, lot: GR243496-3) 1:250-1:500; Rat monoclonal [3H9] anti-GFP (Chromotek, 029762, lot: 80626001AB) 1:200; Recombinant rabbit monoclonal [EPR5145(2)] anti-SPT5 (Abcam, ab126592, lot: GR155828-1) 1:200; Mouse monoclonal [3F2/4] anti-CHD4 (Abcam, ab264521, lot: GR3269483-1) 1:200; Recombinant rabbit monoclonal [EPR14104] anti-GFP (Abcam, ab183734, lot: GR298298-24) 1:200; Rabbit polyclonal anti-Histone H3 (Abcam, ab1791, lot: GR65697-1) 1:200; Mouse monoclonal (DM1A) anti-α-Tubulin (Cell Signaling, 3873, lot: 15) 1:800; Rabbit polyclonal anti-SPT5 (Thermo Fisher Scientific, PA5-56100, lot: 000012280) 1:25-1:50; Rabbit monoclonal [EPR21954] anti-TATA binding protein (TBP) (Abcam, ab220788, lot: GR3357208-3) 1:1000; Rabbit monoclonal [D2N5G] anti-β-Tubulin (Cell Signaling Technology 15115, lot: 3) 1:1000; Mouse monoclonal (9A3) anti-DYKDDDDK Tag (Cell Signaling Technology 8146, lot: 5) 1:800; Goat anti-mouse IgG (H + L), Alexa Fluor® 488, conjugated (Thermo Fisher Scientific, A-11029) 1:2000; Goat anti-mouse IgG (H + L), Alexa Fluor® 594, conjugated (Thermo Fisher Scientific, A-11032) 1:2000; Goat anti-rat IgG (H + L), Alexa Fluor® 488, conjugated (Thermo Fisher Scientific, A-11006) 1:2000; Goat anti-rabbit IgG (H + L), Alexa Fluor® 594, conjugated (Thermo Fisher Scientific, A-11037) 1:2000; Goat anti-Rat IgG (H + L) Secondary Antibody, Biotin (Thermo Fisher Scientific, A18869) 1:200; DyLight® 488 Streptavidin (Vector Laboratories, SA-5488-1) 1:200.

### Cell lines

IMR90 human fibroblasts (isolated from normal lung tissue derived from a 16-week-old female, CCL-186) were purchased from the American Type Culture Collection (ATCC). BJ human normal foreskin fibroblasts derived from a neonatal male were obtained from ATCC (CRL-2522). To generate BJ cells expressing ER:RAS^G12V^, IMR90 cells expressing ER:myrAKT, ER:RAS^G12V^, human papillomavirus 16 proteins E6/E7, HRAS^G12A^, HRAS^G12C^, HRAS^G12D^, HRAS^G12S^, HRAS^Q61R^, or BRAF^V600E^, retroviral infections were carried out as previously described^33,70^. To generate IMR90 expressing shRNAs against p16 or p53, lentiviral infections were carried out as previously described^70^. All cells were cultured in DMEM (Gibco) supplemented with 10% fetal bovine serum (FBS, Sigma) and 1% antibiotic-antimycotic solution (Gibco). To induce expression of Hras^G12V^, IMR90 ER:RAS cells, IMR90 E6/E7 ER:RAS, BJ ER:RAS, and HBF ER:RAS cells were treated with 100 nM 4-hydroxytamoxifen (4OHT, Sigma) continuously, and media were exchanged every three days. If not otherwise indicated, IMR90 RAS cells were induced for 6 days, IMR90 E6/E7 RAS cells were induced for 3 days, and BJ ER:RAS and HBF ER:RAS cells were induced for 7 days. For therapy-induced senescence, IMR90 cells were treated with 0.5 µM Doxorubicin (Sigma) for 24 h, 35 µM Etoposide (Sigma) for 48 h, 1 µM Palbociclib (Selleckchem) (continuously, change of media every 3 days), or γ-irradiated with 10 Gy. MC38 mouse cells were a gift from Nuh Rahbari and have been described previously^41,42^.

### Mice experiments

For expression of NRAS^G12V^ in the liver, 4-8-week-old female C57BL/6 or NSG mice were purchased from Charles River UK, Ltd. Intrahepatic delivery of pCaNIGmirE-5’ (NRAS^G12V^) and pPGK-SB13 was performed via hydrodynamic tail vein injection (HDTVI) as previously described^4^. Treatment with SF3B1 small molecule inhibitors or indisulam was carried out from day 5 on for 5 consecutive days (intraperitoneal injection once per day). Mice were sacrificed on day 10. Mice were monitored to ensure humane endpoints approved by Imperial College animal welfare and ethical review board were not exceeded. Mice were kept under specific pathogen­free barrier conditions within individually ventilated cages on a 12­hour light/dark cycle between 21–23 °C. Mice were given *ad libitum* access to standard rodent chow and water. For analysis of NRAS-GFP+ cells by immunohistochemistry, the tissue was fixed in formalin 10%. For the cytochemical assay of SA-β-gal activity, tissue was embedded in OCT compound and frozen.

*LSL-Kras*^*G12D*^^71^ and *Rosa26loxP-STOP-loxP-YFP/* + ^72^ mice have been previously described. For the experiment, male and female in a mixed genetic background were used. The lung tumour induction experiments were performed as previously described^73^. Briefly, for lung tumour induction in *Kras*^*G12D/+*^*;Rosa26loxP-STOP-loxP-YFP/+* mice, Ad5CMVCre (AdCre) adenoviral particles (University of Iowa, Viral Vector Core) were diluted in DMEM to reach a concentration of 5 × 10^5^ particles/μl, and CaCl_**2**_ was added to reach a final concentration of 4 mM. Six to eight-week-old mice were anaesthetised by a single intraperitoneal injection of a Domitor/Ketamine solution (0.3 mg/kg Domitor (Vetoquinol) and 60 mg/kg Ketamine (Dechra) in 0.9% NaCl). Once anaesthetised, 25 μl of the Ad5CMVCre solution was delivered under each nostril per mouse (i.e., 50 μl of the solution used in total per mouse) for passive inhalation. Following the delivery of AdCre, mice were given an intraperitoneal injection of 1 mg/kg of Antisedan (Zoetis) in 0.9% NaCl to reverse the sedative and analgesic effects. Mice were then allowed to recover on a heated mat. Mice were sacrificed two months after induction. Mice were housed in the Biological Services Unit at University College London under specific pathogen–free conditions in individually ventilated cages (IVCs) with environmental enrichment. Animals were maintained on a 12-hour light/12-hour dark cycle at 20–24 °C with 45–65% relative humidity, and provided with standard rodent chow and water ad libitum. Animal health and welfare were monitored daily in line with ARRIVE 2.0 guidelines.

To assess the effects of SF3B1 inhibitors on RAS-driven liver cancer in vivo, 8-week-old male C57BL/6 mice were acquired from Janvier Labs, and plasmids (pT3-EF1a-KRAS^G12D^-IRES-EGFP (KRAS^G12D^), CRISPR/Cas9 px330-sgTRP53 (TRP53), pCMV-SB13 (Sleeping Beauty)) were delivered to the livers via HDTVI. Animals received intraperitoneal injections of vehicle or E7107 (2 mg/kg/day) 3 times per week starting 4 days after HDTVI. Mice were killed 23 days after HDTVI, and organs were harvested and weighed. Tumour burden was quantified macroscopically from the median lobes. None of the tumours in Fig. 9 exceeded 1 cm^3^, in accordance with the permit G139/19. Mice were kept under specific pathogen­free barrier conditions within individually ventilated cages on a 12­hour light/dark cycle between 21–23 °C. Mice were given *ad libitum* access to standard rodent chow and water.

The MC38 CRC liver metastasis mouse model, as well as the KPC PDAC mouse model, have been described previously in refs. ^41–45^. For the KPC PDAC mouse model, mice of both sexes were used, in approximately equal proportion. Close monitoring ensured humane endpoints approved by the University of Glasgow animal welfare and ethical review board were not exceeded. Mice were culled when exhibiting moderate clinical signs of tumour development (swollen abdomen, loss of body conditioning, yellowing of skin on ears and feet, whitening of feet and/or ear tips, diarrhoea, altered respiration) or general clinical signs of ill health (piloerection, hunched appearance, subdued behaviour, reduced mobility, excessive aggression to cage mates) not improving within a working day. Mice were provided with standard diet and water ad libitum and maintained in conventional cages with tissue, fun tunnels, and nesting material for social, environmental and behavioural enrichment. Where possible, single housing of mice was avoided.

### Vector construction

pLNC ER:RAS^G12V^ and pLNCX2 E6/E7 have been described previously in refs. ^74,75^. pCaNIGmirE-5’ (NRAS^G12V^) and pPGK-SB13 were a gift from Lars Zender^4^. pT3-EF1a-KRAS^G12D^-IRES-EGFP (KRAS^G12D^), px330-sgTRP53 and pCMV-SB13 have been described before^40,76^ and were a gift from Darjus Tschaharganeh. pBabe puro empty vector was a gift from Hartmut Land, Jay Morgenstern, and Bob Weinberg (Addgene plasmid # 1764; http://n2t.net/addgene:1764; RRID: Addgene_1764)^77^. pBabe puro HRAS^G12V^ vector (referred to as HRAS V12) was a gift from William Hahn (Addgene plasmid # 9051; http://n2t.net/addgene:9051; RRID:Addgene_9051). 901 pLNCX myr HA Akt1 was a gift from William Sellers (Addgene plasmid # 9005; http://n2t.net/addgene:9005; RRID:Addgene_9005)^78^. HRAS mutants G12A, G12C, G12D, G12S, and Q61R were generated by site-directed mutagenesis using the QuikChange II kit (Agilent, 200523) according to the manufacturer’s instructions. Mutagenic primers were designed using the web-based QuickChange Primer Design Program available online at www.agilent.com/genomics/qcpd. The sequences can be found in Supplementary Table 1. pBabe B-Raf V600E was a gift from Channing Der (Addgene plasmid # 17544; http://n2t.net/addgene:17544; RRID:Addgene_17544). The nonsense-mediated decay reporter construct 3×FLAG_NanoLuc_HBB_PTC39-pCI-Neo was custom-synthesised by GenScript Biotech (USA), and its sequence is provided in Supplementary Table 2. The insert was subsequently cloned into the pLVX-IRES-Puro vector using the XhoI and XbaI restriction sites. The pLVX-IRES-Puro plasmid backbone was obtained from Takara Bio (USA).

### Cell fractionation and immunoblot

Cell fractionation was carried out as previously described in ref. ^79^. Hypotonic buffer and detergent were purchased (Nuclear Extract Kit, Active Motif, 40010). Cells were trypsinised, washed with ice-cold PBS, and resuspended in a hypotonic buffer. After 30 min., detergent was added, and cells were vortexed briefly to release nuclei. After centrifugation, the supernatant (cytosolic fraction) was collected, and nuclei were washed with hypotonic buffer. Nuclei were lysed with RIPA buffer (80 mM Tris at pH 8.0, 150 mM NaCl, 1% Triton X-100, 0.5% Na-Doc, 0.1% SDS, 1 mM EDTA) supplemented with one tablet of phosphatase and one tablet of protease inhibitors (Roche) per 10 mL of RIPA. Pierce™ BCA Protein Assay Kit (Thermo Fisher Scientific, 23227) was used to determine protein concentrations. For Western Blot, protein lysates were denatured in Laemmli sample buffer (Bio-Rad, 1610747), run on precast gels (Bio-Rad), and transferred to nitrocellulose membranes. Membranes were blocked with 5% milk (w/v) in PBS for 1 h and incubated with primary antibodies overnight at 4 °C. After three washes with PBS, membranes were incubated with secondary antibodies conjugated with horseradish peroxidase (HRP) and visualised using an ECL system (Amersham ECL Prime Western Blotting Detection Reagent, Cytiva, RPN2236). Protein bands were detected with AMERSHAM IMAGER 680 (GE Healthcare).

### Sample processing for mass spectrometry

IMR90 vector cells and IMR90 ER:RAS cells (*n* = 5 biological replicates of each) were treated with 4OHT and collected for fractionation 6 days after 4OHT treatment. Samples (100 µg/sample) were processed using the Filter Aided Sample Preparation (FASP) protocol^80^. A similar protocol has been described before^81^. Briefly, samples were loaded onto 30 kDa centrifugal concentrators (Millipore, MRCF0R030) to carry out buffer exchange by centrifugation on a benchtop centrifuge (15 min, 12,000 g). Several buffer exchanges were performed using UA buffer (8 M urea in 20 mM HEPES, pH=8), reduction with 10 mM DTT in UA buffer (30 min, 40 °C), and alkylation with 50 mM chloroacetamide in UA buffer (30 min, 25 °C). Later, buffer exchange into UA buffer and 50 mM ammonium bicarbonate was performed. Digestion was done using mass spectrometry-grade trypsin (Promega, V5280). Tryptic peptides were collected by centrifugation into a fresh collection tube (10 min, 12,000 g) and washed off the concentrator with 0.5 M sodium chloride for maximal recovery. Following acidification with 1% trifluoroacetic acid (TFA) to a final concentration of 0.2%, collected protein digests were desalted using Glygen C18 spin tips (Glygen Corp, TT2C18.96) and peptides eluted with 60% acetonitrile, 0.1% formic acid (FA). Eluents were then dried using vacuum centrifugation.

### Liquid chromatography-tandem mass spectrometry (LC-MS/MS) analysis

This was performed using the protocol described in ref. ^82^. Briefly, dried tryptic digests were redissolved in 0.1% TFA by shaking (1200 rpm) for 30 min and sonication on an ultrasonic water bath for 10 min, followed by centrifugation (16,000 g, 5 °C) for 10 min. LC-MS/MS analysis was performed using an Ultimate 3000 RSLC nano liquid chromatography system (Thermo Scientific) coupled to a Q-Exactive HF-X mass spectrometer (Thermo Scientific) via an EASY spray source (Thermo Scientific). For LC-MS/MS analysis, redissolved protein digests were injected and loaded onto a trap column (Acclaim PepMap 100 C18, 100μm × 2 cm) for desalting and concentration at 8 μL/min in 2% acetonitrile, 0.1% TFA. The final on-column peptide concentration was 1500 ng per injection. Peptides were then eluted online to an analytical column (Acclaim Pepmap RSLC C18, 75μm × 50 cm) at a flow rate of 250nL/min. Peptides were separated using a 120-minute gradient, 4-25% buffer B for 90 min, followed by 25-45% buffer B for another 30 min (composition of buffer B – 80% acetonitrile, 0.1% FA) and subsequent column conditioning and equilibration. Eluted peptides were analysed by MS operating in positive polarity using a data-dependent acquisition mode. Ions for fragmentation were determined from an initial MS1 survey scan at 120,000 resolution, followed by HCD (Higher Energy Collision Induced Dissociation) of the top 30 most abundant ions at 15,000 resolution. MS1 and MS2 scan AGC targets were set to 3 × 10^6^ and 5 × 10^4^ for maximum injection times of 25 ms and 50 ms, respectively. A survey scan m/z range of 350-1750 was used, normalised collision energy set to 27%, charge exclusion enabled with unassigned, and +1 charge states rejected, and a minimal AGC target of 8e3.

### Mass spectrometry raw data processing

Data were processed using MaxQuant (v1.6.2.3), with database searches carried out with the in-built Andromeda search engine against the SwissProt *H. sapiens* database (version 20190621, number of entries: 20,444). A reverse decoy search approach was used at a 1% false discovery rate (FDR) for both peptide spectrum matches and protein groups. Search parameters included: maximum missed cleavages set to 2, fixed modification of cysteine carbamidomethylation, and variable modifications of methionine oxidation and protein N-terminal acetylation. Label-free quantification was enabled with an LFQ minimum ratio count of 1. The ‘Match between runs’ function was used with match and alignment time limits of 0.7 and 20 min, respectively.

### Analysis of proteomics data

Analysis was performed using the Jupyter Notebook (https://jupyter.org/) with Python and R scripts. The output protein group file of the MaxQuant program was used to extract and process the data using the SciPy Python ecosystem^83^. Protein groups annotated by the MaxQuant program as ‘Only identified by site’ (*n* = 31), ‘Reverse’ (*n* = 45), and ‘Potential contaminant’ (*n* = 53) were removed from the analysis. Protein groups identified with less than 2 unique peptides were removed from the analysis (*n* = 485). Moreover, protein groups with an Andromeda score of less than 20 were filtered out. The value distribution of the Lfq data was visualised with a box plot. Principal Component Analysis of the top 500 expressed genes was used to evaluate the separation between IMR90 control (con, *n* = 5) and IMR90 RAS (ind, *n* = 5) samples. We performed sample-specific imputation of the missing MaxQuant Lfq data using random draws from a Gaussian distribution centred on the minimal value of each sample. The sample reproducibility was evaluated by computing the Pearson correlation coefficient (PCC) of each data column across replicas and between conditions. The PCC values were visualised with a heat map. The Lfq values were log_2_ transformed, and the R limma package^84^ was used to identify proteins with different abundance between the nuclei of control and senescent samples using a linear model with empirical Bayes statistics. The distribution of the obtained p-values was visualised with a histogram. Pathway analysis was conducted with DAVID 6.8, including all significantly upregulated proteins in IMR90 RAS vs IMR90 control cells and using all identified nuclear proteins as background (https://david.ncifcrf.gov/home.jsp)^85,86^. To further analyse the data, we manually curated a list to identify splicing factors among the detected nuclear proteins. We included 244 proteins that were purified repeatedly with spliceosomal complexes^87^ as well as all proteins in the MaxQuant protein group output file described as “splicing factors”. log_2_ fold change values and -log_10_ p values obtained in the limma analysis were used to visualize the proteome in IMR90 RAS vs IMR90 control cells (splicing factors vs all other identified proteins, Fig. 1c). To compare and analyze the changes observed on protein level with transcriptomics of senescent vs control cells, we used gene expression data obtained by RNA sequencing as described below. The gene name of the leading protein in each protein group of the MaxQuant output file was extracted. Afterwards, gene names were used to find the log2 fold change on the RNA level. log_2_ fold change values were visualised with a scatter plot, and association was evaluated by simple linear regression and Pearson correlation (Fig. 1e).

### Drugs and treatments

Trametinib (GSK1120212) (S2673), Liproxstatin-1 (S7699), Necrostatin-1 (S8037), and VX-765 (S2227) were purchased from Selleckchem. Pladienolide B (CAS 445493-23-2) was purchased from Santa Cruz Biotechnology (sc-391691). Indisulam (CAS 165668-41-7) was obtained from Merck (SML1225). E7107 (CAS 630100-90-2) was a gift from H3 Biomedicine. Emricasan (HY-10396), Q-VD-OPh (HY-12305), Ferrostatin-1 (HY-100579), Necrosulfosamine (HY-100573), Ac-YVAD-cmk (HY-16990), Rapamycin (HY-10219), Temsirolimus (HY-50910), Torin-1 (HY-13003), LY2584702 (HY-12493A), PF708671 (HY-15773), BMS-582949 (HY-14305A), SB203580 (HY-10256) were purchased from MedChemExpress.

For tissue culture experiments, all compounds were reconstituted in DMSO and added to the media at the indicated concentrations. IMR90 ER:RAS cells were induced with 4OHT for 6 days, and IMR90 RAS and IMR90 control cells were treated with splicing inhibitors and SPT5 inhibitors from day 6 to day 10 (4 days), and with the cell death inhibitors from day 6 to day 10 (3 days), unless otherwise stated. IMR90 E6/E7 ER:RAS cells were induced with 4OHT for 3 days, and IMR90 E6/E7 RAS and IMR90 E6/E7 control cells were treated with splicing inhibitors and SPT5 inhibitors for 4 days. For analysis of SPT5 protein expression, IMR90 ER:RAS cells were treated with 30 nM Pladienolide B for 24 h.

For the treatment of mice, E7107 was reconstituted in sterile PBS with 4% Tween 80 and 10% Ethanol. Pladienolide B and Indisulam were reconstituted in a 5% glucose solution with 6.5% Tween 80 and 3.5% DMSO. Dissolved compounds were filtered through 0.2 µm sterile filters. Mice were weighed every two days to monitor toxicity and to allow weight-based drug dosing. All compounds were given intraperitoneally at the following dosages: E7107, 2 mg/kg/day, Pladienolide B, 10 mg/kg/day, and Indisulam, 25 mg/kg/day.

### Growth and viability assays, live cell imaging

For assessment of viability, cells were fixed in 4% paraformaldehyde (w/v) and stained with 1 µg/ml DAPI (Sigma). High content analysis was used to determine the number of living cells by counting the nuclei per well (see high content analysis). The percentage of living cells was calculated by dividing the number of cells after drug treatment/after siRNA transfection by the number of cells treated with vehicle/transfected with non-targeting siRNAs. For colony-forming assays/crystal violet staining, cells were seeded in 6 cm dishes. Cells were fixed with 0.5% glutaraldehyde (w/v), and plates were then stained with 0.2% crystal violet (w/v). Live cell imaging was carried out using the IncuCyte System (Sartorius) and CellEvent™ Caspase-3/7 Green Detection Reagent (Thermo Fisher Scientific, C10423).

### Immunofluorescence staining of cells

Cells were grown in 96-well plates and fixed with 4% paraformaldehyde (w/v). After permeabilisation with 0.2% Triton X-100 (v/v), the blocking solution (1% BSA (w/v) in PBS) was added. Cells were incubated with primary antibodies in blocking solution for 1 h, then washed 3x with PBS. Secondary antibodies in the blocking solution were added for 45 min. Nuclei were stained with 1 µg/ml DAPI (Sigma) for 15 min.

### Measurement of newly synthesised RNA

Click chemistry staining of 5-ethynyl uridine (Thermo Fisher Scientific, E10345) to quantify newly synthesised RNA was carried out as previously described in ref. ^51^. 0.5 mM 5-ethynyl uridine was added to cells for 3 h before cells were fixed with 4% paraformaldehyde (w/v). After permeabilisation with 0.2% Triton X-100 (v/v), staining solution (100 mM Tris pH 8.5; 1 mM CuSO_4_; 10 µM Alexa Fluor 594 Azide (Alexa Fluor™ 594 Azide, mixed isomers, Thermo Fisher Scientific, A10270); 100 mM ascorbic acid) was added and cells were incubated for 30 min. Cells were washed several times with PBS and counterstained with 1 µg/ml DAPI (Sigma) for 15 min.

### High content analysis

High content analysis was performed as previously described in refs. ^33,70,88^. Imaging was carried out using the automated high-throughput microscope IN Cell Analyzer 2000 or IN Cell Analyzer 2500 HS and the IN Cell Investigator 2.7.3 software (GE Healthcare). To assess the number of living cells per well, images were taken with a 10x objective, and multiple fields were acquired to cover the whole well. For immunofluorescence imaging, a 20x objective was used, and multiple fields were acquired. For all experiments, 3-4 wells per sample were acquired.

### siRNA transfections and siRNA screens

All siRNAs were ordered from Dharmacon. Custom libraries were curated using pooled siRNAs: siRNA library “splicing factors” (Supplementary Data 2) and siRNA “downstream effectors” (Supplementary Data 4). For the “splicing factors” library, 189 splicing factors were selected, including all “core” splicing factors as well as splicing factors significantly upregulated (*p*-value < 0.05; log2 fold change >0.25) in IMR90 RAS vs IMR90 control cells as seen by mass spectrometry^87^. H_2_O and non-targeting siRNA controls were included on all plates. For the “downstream effectors” library, we selected all genes with significant differential splicing and concomitant downregulation at the gene expression level. For retesting of siRNAs, libraries containing individual siRNAs (4 per gene) were used (“splicing factors retesting”, Supplementary Data 3, and “downstream effectors retesting”, Supplementary Data 5). Reverse siRNA transfections were carried out as previously described in refs. ^33,70^. siRNAs at a concentration of 1 µM were preincubated with plain DMEM and DharmaFECT 1 transfection reagent (Dharmacon, T-2001). Cells were trypsinised and resuspended in DMEM containing 10% FCS only (no antibiotics). The media was replaced 18-24 h after transfection. Details of siRNAs used in this study can be found in Supplementary Table 3.

### Design and transfection of antisense oligonucleotides (AONs)

The design of AONs has been described previously in ref. ^33^. Sequences of AONs used in this study are provided in Supplementary Table 5. IMR90 vector and IMR90 ER:RAS cells were reversely transfected after 6 days of treatment with 4OHT, and RNA was collected after another 24 h. For details of reverse transfections, see siRNA transfections above.

### Cytochemical SA-β-galactosidase assay

Cytochemical assay of SA-β-gal activity on frozen tissue sections was carried out as previously described in ref. ^70^. Tissue sections were fixed with 0.5% glutaraldehyde (w/v) for 15 min, washed 3x with PBS, and then incubated with X-gal staining solution (1 mg/mL X-Gal, Thermo Scientific, 5 mM K_3_[Fe(CN)_6_], and 5 mM K_4_[Fe(CN)_6_], in PBS 1 mM MgCl_2_ pH=6) at 37 °C until cells turned blue. Tissues were counterstained with eosin, dehydrated, and permanently mounted. Bright-field images were taken, and the positive area was quantified with Fiji/ImageJ software (https://imagej.net/software/fiji/)^89^. To exclude luminal spaces in the tissue sections, the SA-β-gal positive area was divided by the total area (determined by eosin staining).

### Immunofluorescence staining of tissue sections

Tissues were fixed in 10% formalin solution overnight and embedded in paraffin. Sections of tissue samples were rehydrated for staining. Heat-induced epitope retrieval was carried out using citrate buffer pH 6. Sections were permeabilised with Triton 0.2% (v/v) in PBS and blocked with CAS-Block solution (Thermo Fisher Scientific), followed by incubation with primary antibodies overnight at 4 °C in Dako antibody diluent (Agilent). After washing with PBS (3x10min), IF staining was continued by the addition of secondary antibodies in antibody diluent. For lung sections, YFP was stained with a GFP antibody followed by amplification of signal using a biotinylated secondary antibody and DyLight® 488 Streptavidin (Vector Laboratories). Afterwards, sections were incubated with 1 µg/ml DAPI (Sigma) for 10 min, washed with PBS and ddH_2_O, and mounted with Fluoromount-G aqueous mounting solution (SouthernBiotech, 0100-01).

### Histology and immunohistochemistry staining of tissue sections

Histology, immunohistochemistry, scanning, and automated analysis have been described previously in refs. ^90,91^. IHC staining was also carried out manually (e.g., for quantification of NRAS-positive hepatocytes, staining of SF3B1, RBM39, and SPT5 in the KPC PDAC mouse model). Sections of tissue samples were rehydrated for staining. Heat-induced epitope retrieval was carried out using citrate buffer pH 6. Sections were permeabilised with Triton 0.2% (v/v) in PBS and blocked with CAS-Block solution (Thermo Fisher Scientific), followed by incubation with primary antibodies overnight at 4 °C in Dako antibody diluent (Agilent). Sections were afterwards blocked with H_2_O_2_ 3% (w/w) in PBS for 10 min. After washing with PBS (3x10min), tissue sections were incubated with SignalStain® Boost IHC Detection Reagent (HRP, Rabbit) (Cell Signaling) for 45 min and then washed with PBS (3x10min). Afterwards, SignalStain® DAB Substrate was added for 2-5 min, and sections were immediately washed in ddH_2_O. After counterstaining with hematoxylin, sections were dehydrated and mounted with VectaMount® Permanent Mounting Medium (Vector Laboratories).

### Chemicals for immunohistochemistry staining

The following chemicals and buffers were used for immunohistochemistry staining of paraffin sections: SignalStain® Boost IHC Detection Reagent (HRP, Rabbit) (Cell Signaling, 8114); SignalStain® DAB Substrate Kit (Cell Signaling, 8059); CAS-Block™ Histochemical Reagent (Thermo Fisher Scientific, 008120); Dako Antibody Diluent (Agilent, S0809); VectaMount® Permanent Mounting Medium (Vector Laboratories, H-5000-60); Bond citrate solution (Leica, AR9961); Bond EDTA solution (Leica, AR9640); Bond proteolytic enzyme kit (Leica, AR9551); Bond primary antibody diluent (Leica, AR9352); Bond polymer refine detection kit (Leica, DS9800).

### Total RNA extraction

Cells were scraped and homogenised in 0.8 ml TRIzol^TM^ RNA isolation reagent (Thermo Fisher Scientific, 15596026). After the addition of 200 µl of chloroform, lysates were vortexed and then centrifuged to separate the RNA-containing phase. Further extraction of RNA was performed using the RNAeasy® Mini Kit (Qiagen, 74104). RNA concentration was measured using a NanoDrop® spectrophotometer at an absorbance of 260 nm.

### cDNA synthesis and RT-PCR

cDNA synthesis from total RNA was carried out using random hexamer primers and the SuperScript® II Reverse Transcriptase (RT) kit (Thermo Fisher Scientific, 18064022) according to the manufacturer’s instructions. For quantification of alternative splicing of SUPT5H and CHD4, primers were designed to be specific for either of the two isoforms. Primers were designed using Primer3 (http://bioinfo.ut.ee/primer3/). For absolute quantification of isoforms, standard curves were generated using DNA templates. DNA templates were similar to the expected amplicon of the primer pair, and AAGAA was added to both the 5′ and 3′ ends. This method has been described previously in ref. ^21^. Samples were run on a CFX96™ Real-Time PCR Detection system (Bio-Rad), and PCR amplification was performed using SYBR® Green PCR Master Mix (Applied Biosystems, 4309155). Primer sequences can be found in Supplementary Table 4. For the analysis of alternatively spliced transcripts by microfluidic electrophoresis, a third primer pair was designed to amplify both isoforms of SUPT5H and CHD4, respectively. PCR amplification and fragment analysis on the Agilent 2100 Bioanalyzer were performed as previously described (Georgilis et al., 2018). Primer sequences are listed in Supplementary Table 4.

### RNA-sequencing

For RNA-sequencing and subsequent gene expression analysis of splicing factors, RNA of the IMR90 vector and IMR90 ER:RAS cells were collected on day eight. Four replicates were included in the experiment. For RNA-sequencing and subsequent differential alternative splicing analysis after knockdown of SF3B1 and RBM39, IMR90 control cells, and IMR90 ER: RAS cells were transfected with two different non-targeting siRNAs, two different siRNAs targeting SF3B1, and two different siRNAs targeting RBM39 on day 6. RNA was collected 48 h after transfection. Three replicates were included in the experiment.

RNA-sequencing (RNA-seq) libraries were prepared as previously described in ref. ^33^ and were run on a HiSeq2500. 40-50 million single-end 50 bp reads were obtained per replicate. The quality of the raw sequencing reads was assessed using FASTQC. For gene expression analysis, RNA-seq samples were aligned to the human genome assembly hg19 with Tophat (v.2.0.11)^92^ with parameters ‘–library-type fr-firststrand’ and using Ensembl v.72 gene annotation. Gene expression levels were quantified as the number of uniquely aligned reads overlapping with gene coordinates, using the *feature counts* function from the R package Rsubread^93^. Differentially expressed genes between conditions were identified using the DESeq2 R package^94^, and genes with Benjamini–Hochberg corrected *p*-values  <  0.05 were defined as differentially expressed. For Gene Set Enrichment Analysis (GSEA)^95^, genes were ranked using “Wald statistics” obtained from DESeq2, and GSEA was carried out on all curated gene sets available in MSigDB (http://software.broadinstitute.org/gsea/msigdb). Gene expression quantification considered in the differential alternative splicing analyses was obtained with vast-tools^96–98^ as explained below.

### Differential alternative splicing analysis

Alternative splicing quantification was performed with vast-tools^96–98^ version 2. Reads in FASTQ files were aligned (vast-tools *align* with --expr parameter to include expression quantification) against VAST-DB hg19 annotation for *Homo sapiens*^96^, and alignments from multiple-lane FASTQ files were merged per biological sample (vast-tools *merge*). Summary tables for inclusion level quantification (per cent spliced-in, PSI) of annotated alternative splicing events and gene expression quantifications were obtained with vast-tools *combine*.

To ensure sufficient and reliable read coverage, only events with PSI quantification for all samples and a minimum mappability-corrected read coverage score of “VLOW” across all 24 samples (see vast-tools’ GitHub description manual for details on the number of reads per event type associated with the mappability-corrected coverage score) were considered. To ensure sufficient variability in alternative splicing events across conditions, only events with a minimum PSI range (difference between the maximum and minimum of PSI values across samples) of 5 were considered. Redundant inclusion quantifications for alternative splice site (“Alt3” and “Alt5”) events, i.e., PSIs based on reads that share a splice junction, were identified based on event ID, and one event per set of events reflecting alternative splice site choices was removed. Only intron retention events without statistically significant evidence for a high imbalance based on a binomial test comparing exon-intron junctions and intron body read counts^98^ for all samples were considered.

Differential alternative splicing analyses and visualisation of results were performed using the betAS package^46^, which relies on beta distributions to model the precision of PSI estimates from their supporting coverage (#inc and #exc) extracted from “Quality” columns in vast-tools’ summary table. All contrasts between pairs of conditions (IMR90 vector siNT, IMR90 ER: RAS siNT, IMR90 vector siSF3B1, and IMR90 ER:RAS siSF3B1 for the initial setup (Fig. 5a)) were tested for differential alternative splicing, allowing the ranking of differential alternative splicing events according to a compromise between the magnitude of splicing changes (ΔPSI) and their supporting evidence (P_diff_, probability of differential splicing). For the second experimental setup (Supplementary Fig. 11a), we compared pairs of IMR90 ER:RAS cells transfected with either siNT, siSF3B1, or siRBM39, and treated with either DMSO or 4OHT, to assess the impact of splicing factor knockdown in both control and oncogenic RAS-induced conditions. Under the hypothesis that SF3B1 and RBM39 depletion affects gene expression via splicing inhibition, we performed differential gene expression analysis to identify genes with significant differential alternative splicing and concomitant downregulation of gene expression levels as candidate “downstream effectors”. For this purpose, gene expression quantification was obtained with vast-tools. To ensure enough coverage for reliable differential expression analysis, we empirically applied expression filtering based on the visualisation of distributions of raw read counts. For the gene expression analyses in the original setup (Fig. 5a), only genes with a minimum of 5 raw counts per sample for all samples were considered (12,360 genes out of the 19,509 detected by vast-tools). For the second setup (Supplementary Fig. 11a), we required a minimum of 10,000 total counts across all samples, resulting in 10,623 genes retained out of 19,842 detected. Differential gene expression analysis was performed using R packages limma^84^ and edgeR^99^. Raw library size scaling was performed using the *calcNormFactors* function (edgeR), and linear modelling (*lmFit* function) was applied on normalised read counts (with *voom* function) for all contrasts between pairs of conditions. For each contrast, we obtained each gene’s log_2_ fold-change in expression, with its significance given by the FDR-adjusted p-value of an empirical Bayes t-statistic corrected to have its standard errors moderated across genes^100^.

Linear modelling with the same design was applied to logit-transformed PSIs of events considered “alternative”, i.e., that have 1 $\le$ PSI $\le$ 99 for all samples, using the *logit* function from the car R package. Empirical Bayes statistics (B-statistics) giving the log-odds ratios of differential gene expression and alternative splicing were obtained with limma’s *eBayes* function. Concomitant analysis of differential gene expression and alternative splicing results allowed selection of candidate downstream effectors of the spliceosome impairment induced by SF3B1 depletion as genes with significant differential alternative splicing (P_diff_ > 0.95 and B-statistic _diff. splicing_ > 0) and downregulation of gene expression (B-statistic _diff. gene expression_ > 0 and log_2_ fold-change _diff. gene expression_ < 0) in either IMR90 ER:RAS siSF3B1 vs IMR90 vector siSF3B1 or IMR90 ER:RAS siSF3B1 vs IMR90 ER:RAS siNT contrasts. The events selected in the analyses of the initial setup, specifically those in *CHD4* and *SUPT5H*, were further explored in the new setup by comparing the t-statistics or ΔPSI of siNT against siRBM39 or siSF3B1, in IMR90 ER:RAS cells exposed to DMSO or 4OHT.

### Software

The following additional software was used in this study: GraphPad Prism 9 (https://www.graphpad.com/scientific-software/prism/); GSEA version 2.0.12 (https://software.broadinstitute.org/geas/msigdb^95,101^; DAVID 6.8 (https://david.ncifcrf.gov/home.jsp)^85,86^; Cancer Dependency Map (https://depmap.org/portal/); Fiji/ImageJ (https://imagej.net/software/fiji/)^89^; Aperio ImageScope (ver. 12.4.0.5043) (Leica, https://www.leicabiosystems.com/en-de/digital-pathology/manage/aperio-imagescope/).

### Database analysis

Analysis of cancer cell dependencies and gene expression was carried out using the Cancer Dependency Map data explorer (https://depmap.org/portal/interactive/).

### Statistical analysis

GraphPad Prism version 9 was used for statistical analysis. Two-tailed, unpaired Student’s t-test was used to compare the two groups. One-way or two-way analysis of variance (ANOVA) with Dunnett’s or Sidak’s multiple comparisons test was used to compare three or more groups. Values are represented as mean values with standard deviation unless otherwise indicated. ns = not significant. For in vivo studies, mice were randomly assigned to treatment groups. For in vitro studies, all replicates represent independent experiments. For in vivo studies, replicates represent individual mice.

### Reporting summary

Further information on research design is available in the Nature Portfolio Reporting Summary linked to this article.

## Supplementary information

Supplementary information

Description of Additional Supplementary Files

Sup Dataset 1

Sup Dataset 2

Sup Dataset 3

Sup Dataset 4

Sup Dataset 5

Reporting Summary

Transparent Peer Review file

## Source data

Source Data

## Data Availability

RNA-seq data generated in this study have been deposited in the GEO database under accession codes GSE209624, GSE162175, and GSE297268. Mass spectrometry data are deposited in the ProteomeXchange database under accession code PXD035299. The remaining data are available within the Article, Supplementary Information or Source Data file. Source data are provided with this paper.

## Code availability

The files and scripts used for the analysis of the proteomics data are deposited on GitHub (mtinti/spliceosome-senescence) and archived at Zenodo (10.5281/zenodo.7858968)^102^.
