# Supplementary material for: Spliceosome induction is a druggable dependency of RAS-driven senescence and cancer
Source: Nat Commun. 2026 Apr 15;17:5208. doi: 10.1038/s41467-026-71564-z (PMC13254117; doi:10.1038/s41467-026-71564-z)
Supplement: Supplementary file 1 — Supplementary information [file 41467_2026_71564_MOESM1_ESM.pdf]

SUPPLEMENTARY INFORMATION for

Spliceosome induction is a druggable dependency of RAS-driven senescence and cancer

Verena Wagner, Laura Bousset, Mariana Ascensão-Ferreira, Bin Sun, José Efren Barragan Avila, Alexandre Kaizeler, Rita Martins-Silva, Mohammad Rahbari, Mirian Fernández-Vaquero, Scott Haston, Michele Tinti, Joaquim Pombo, Sanjay Khadayate, Susanne Roth, Christian M. Schürch, Juan Pedro Martínez-Barbera, Anat Bahat, Keng Boon Wee, Jennifer P. Morton, Nisar Malek, Andrew J. Innes, Santiago Vernia, Nuno L. Barbosa-Morais, Suchira Gallage, Mathias Heikenwalder and Jesús Gil

Including:

- **18 Supplementary Figures and their legends.**
- **5 Supplementary Tables:**
  - Supplementary Table 1. Primer sequences for mutagenesis
  - Supplementary Table 2 Sequence of 3xFLAG_NanoLuc_HBB PTC39
  - Supplementary Table 3. siRNAs
  - Supplementary Table 4. Primer sequences
  - Supplementary Table 5. AON sequences
- **3 Supplementary Source Data (uncropped western blots):**
  - Supplementary Source Data 1. Relative to Supplementary Figure 2a
  - Supplementary Source Data 2. Relative to Supplementary Figure 2f
  - Supplementary Source Data 3 Relative to Supplementary Figures 13c, 13f

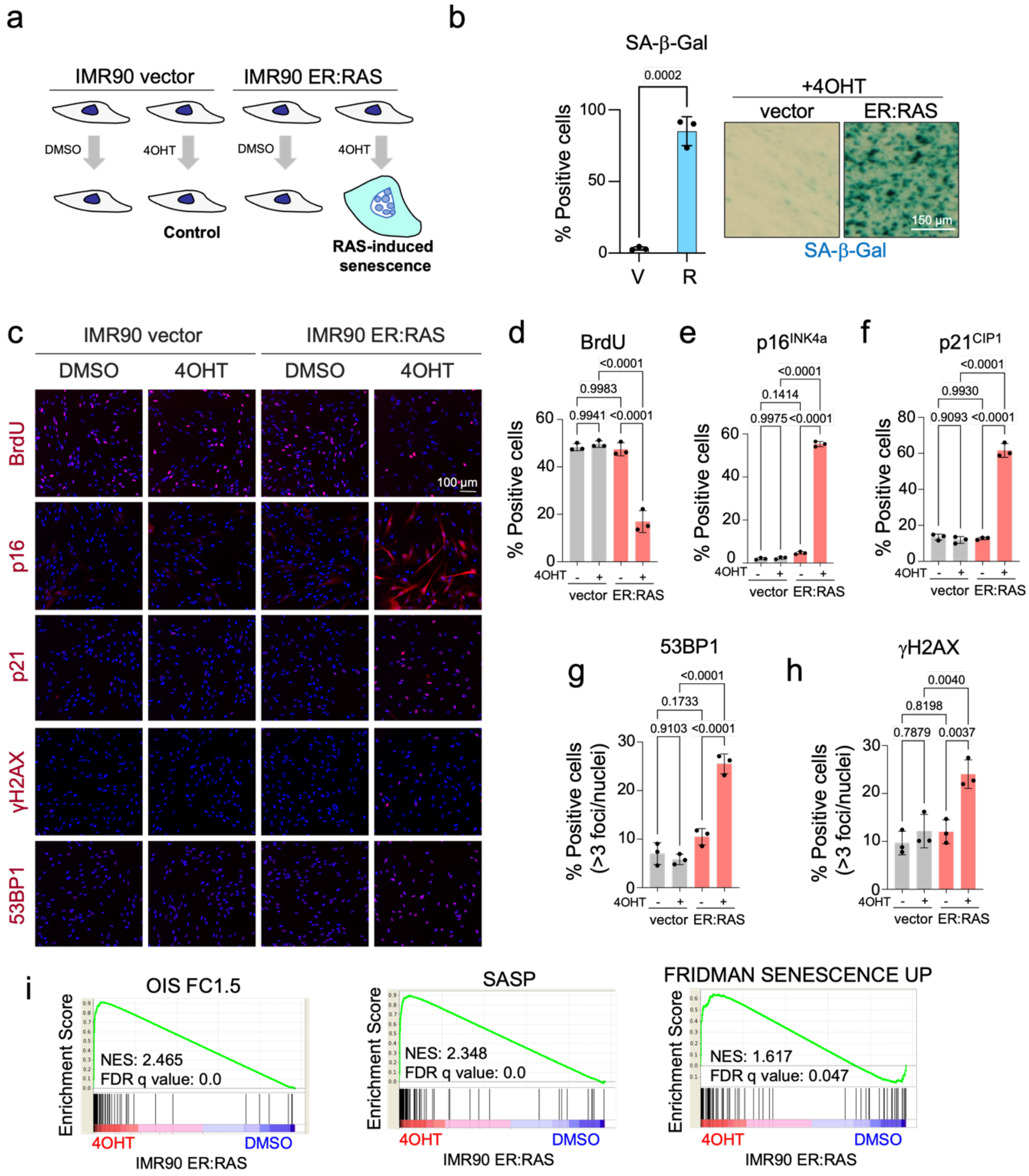

Supplementary Figure 1. IMR90 ER:RAS as a system of oncogene-induced senescence. Related to Figure 1. a, IMR90 cells expressing a 4OHT inducible version of oncogenic RAS (IMR90 ER: RAS) and their corresponding controls (IMR90 vector) were treated with DMSO or 4OHT (which activated the ER:RAS chimeric protein in IMR90 ER:RAS cells). Samples were collected six days after treatment with 4OHT. Highlighted are the control and RAS-induced senescence conditions used across the paper. **b,** Representative images (right) and quantification of the percentage of cells positive for SA-β-Gal staining (left) in the indicated cells. IMR90 Vector + 4OHT (vector, V) versus IMR90 ER:RAS + 4OHT (RAS, R). Average values, mean, and standard deviation of n = 3 independent experiments are shown. Unpaired

t-test, two-tailed. **c-h,** Representative images (**c**) and quantitation of immunofluorescence staining (**d-h**) of the indicated cells (at day 6 post-induction). Results show the percentage of positive cells for BrdU incorporation (**d**), expression of $p16^{INK4a}$ (**e**) or $p21^{CIP1}$ (**f**), or cells showing more than three 53BP1 (**g**) or γH2AX (**h**) foci per nucleus. Average values, mean, and standard deviation of n = 3 independent experiments are shown. Ordinary one-way ANOVA with Dunnett's multiple comparisons test. ns, not significant. **i,** Gene set enrichment analysis plots (GSEA) for senescence-related signatures. The OIS FC1.5 and SASP signatures were derived from [32]. NES, normalised enrichment score. FDR, false discovery rate. Source data are provided as a Source Data file.

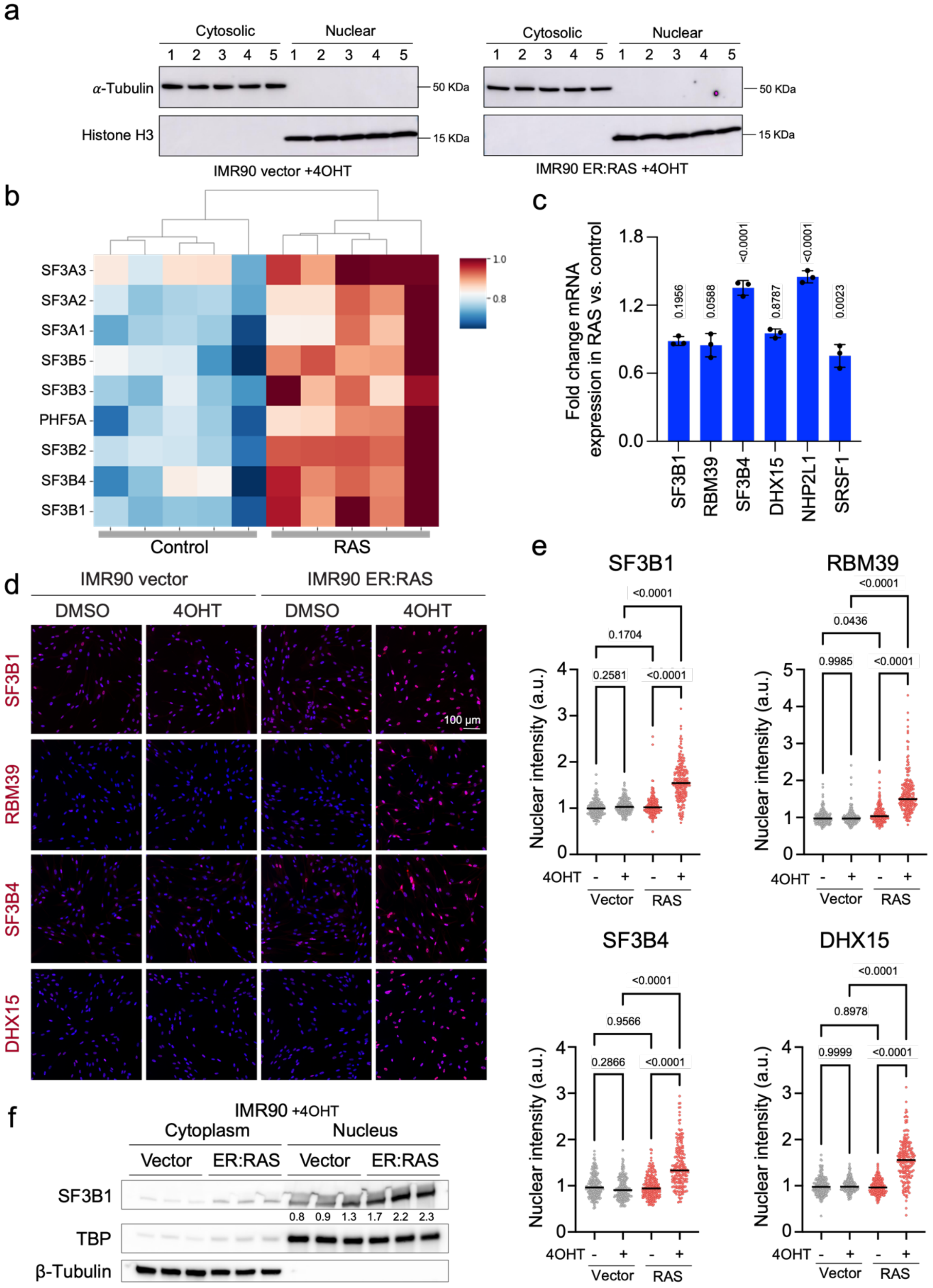

Supplementary Figure 2. Upregulation of splicing factors in cells undergoing RAS-induced senescence. Related to Figure 1. a, Immunoblots of nuclear and cytosolic fractions. Biological replicates were used to carry out mass spectrometry. (n = 5). Antibodies against β-Tubulin and TBP were used as loading controls for cytoplasmic and nuclear proteins, respectively. **b,** Heatmap showing upregulation of multiple U2 snRNP components in the nuclei

of RAS-expressing cells. Heatmap derived from the MS data. Biological replicates were used to carry out mass spectrometry. (n = 5). **c,** Fold change in the mRNA levels of the indicated splicing factors in IMR90 ER:RAS relative to IMR90 vector cells at day 6 post-treatment with 4OHT. Average values, mean, and standard deviation of n = 3 independent experiments are shown. Ordinary one-way ANOVA with Dunnett's multiple comparisons test. ns, not significant. **d-e,** Representative images (d) and quantification (e) of nuclear intensities of the indicated splicing factors in IMR90 ER:RAS (RAS) or IMR90 vector (vector) cells. Single-cell nuclear intensities and mean values, n = 200 cells per condition. Representative data of one out of three independent experiments. Scale bar, 100 µm. Representative data of one out of three independent experiments. Ordinary one-way ANOVA with Dunnett's multiple comparisons test. ns, not significant. **f,** Immunoblot of SF3B1 from nuclear and cytosolic fractions and quantification of the relative abundance compared to TBP. Antibodies against β-Tubulin and TBP were used as loading controls for cytoplasmic and nuclear proteins, respectively. n = 3 biological replicates. Source data are provided as a Source Data file. Uncropped western blots are shown at the end of the Supplementary Information.

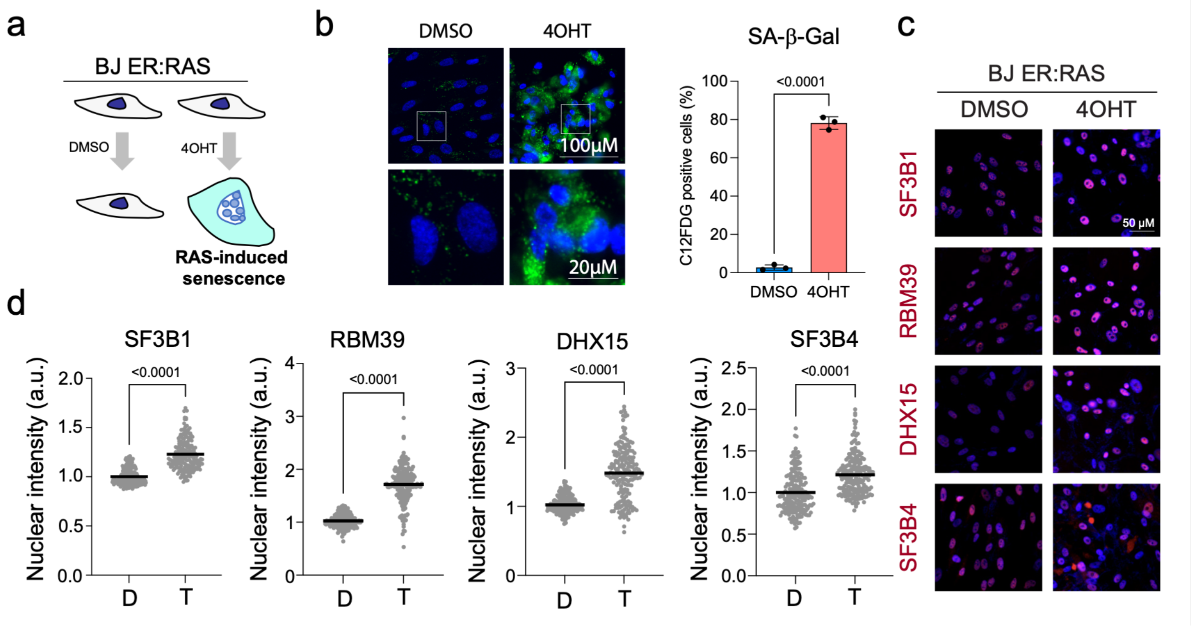

Supplementary Figure 3. Expression of splicing factors in RAS^{V12}-induced senescent BJ fibroblasts. Related to Figure 2. a, BJ ER:RAS^{V12} fibroblasts were treated for seven days with DMSO (control condition) or with 4OHT to induce the expression of the RAS^{V12} oncogene and their subsequent entry into senescence. **b,** Representative images (left) and quantification (right) of the SA-β-galactosidase staining using C12-FDG fluorescent substrate. Mean and standard deviation. n = 3 independent experiments. Scale bar, 100 μm; higher magnification: scale bar, 20 μm. Unpaired t-test, two-tailed. **c-d,** Representative images (**c**) and quantification (**d**) of nuclear intensities of the indicated splicing factors in BJ ER:RAS treated with DMSO (D) or 4OHT (T). Representative data of one out of three independent experiments. Single-cell nuclear intensities and mean values, n = 200 cells per condition. Unpaired t-test, two-tailed. Source data are provided as a Source Data file.

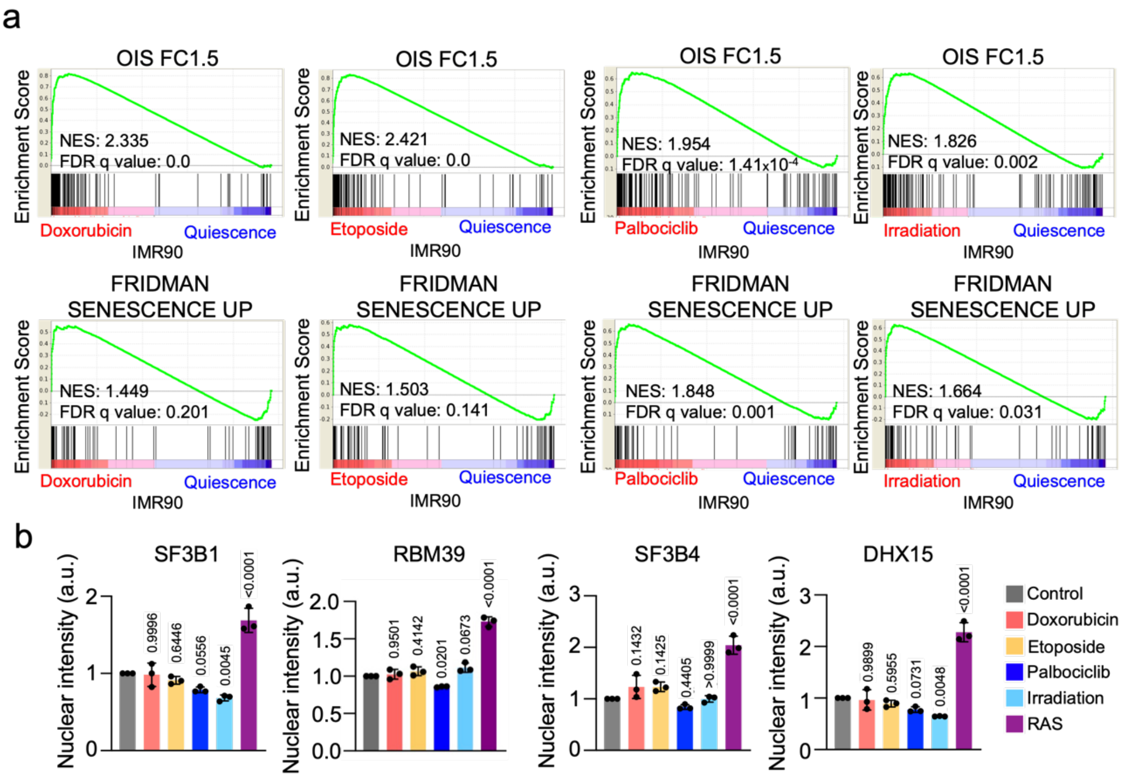

Supplementary Figure 4. Expression of splicing factors in different types of senescence. Related to Figure 2. a, Gene set enrichment analysis plots (GSEA) for senescence-related signatures. The OIS FC1.5 signature was derived from [32]. NES, normalised enrichment score. FDR, false discovery rate. **b,** Quantification of nuclear levels of the indicated splicing factors in IMR90 cells undergoing senescence, as indicated or their corresponding controls. Average values, mean, and standard deviation of n = 3 independent experiments are shown. Ordinary one-way ANOVA with Dunnett's multiple comparisons test. All comparisons are referred to the control. ns, not significant. Source data are provided as a Source Data file.

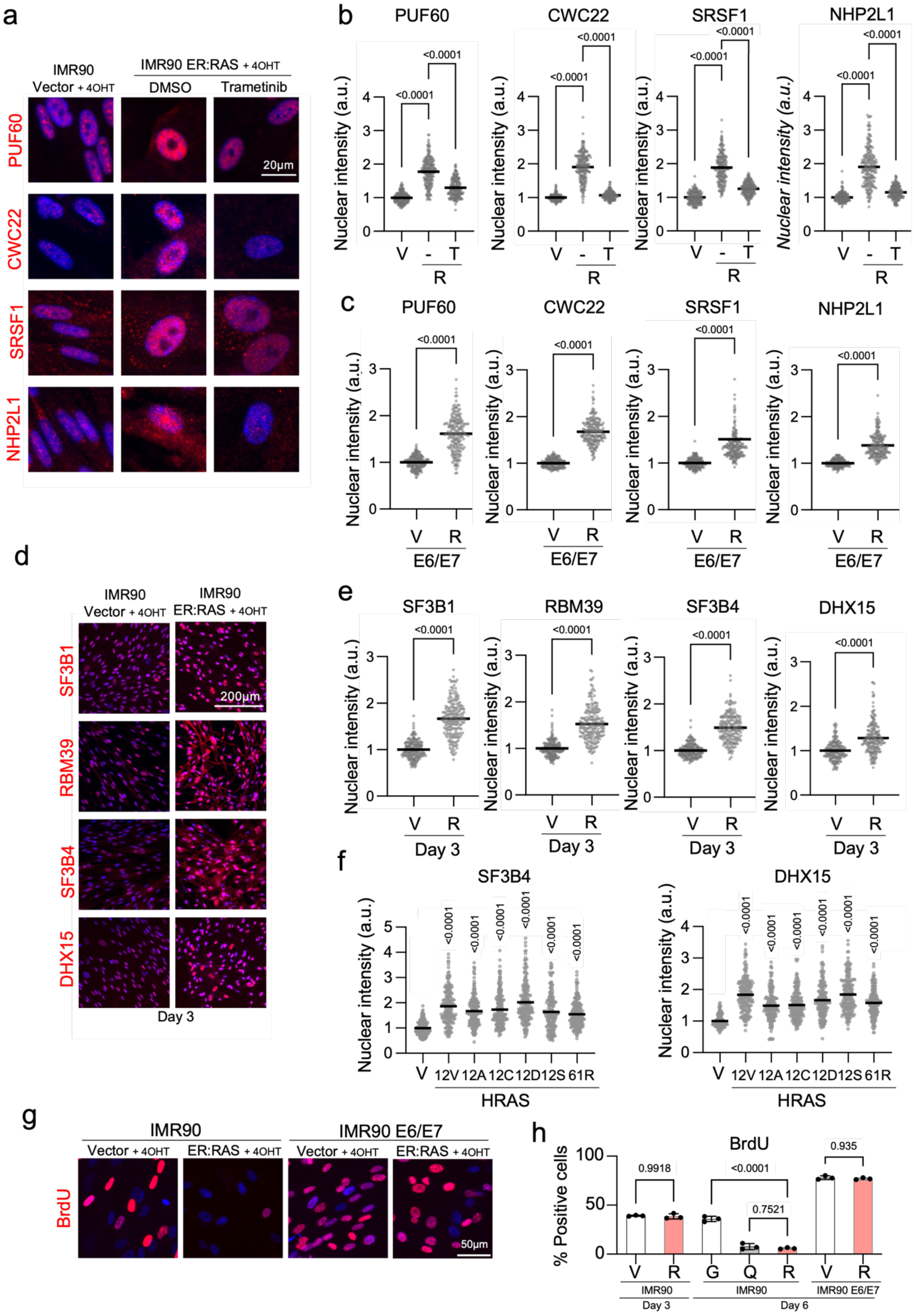

Supplementary Figure 5. Global upregulation of spliceosome components is associated with RAS activation. Related to Figure 2. a -b, Immunofluorescence staining of different splicing factors in control IMR90 cells (vector +4OHT, indicated by V) or IMR90

ER:RAS cells undergoing senescence (IMR90 ER:RAS +4OHT, indicated by R) without (DMSO, -) or with treatment with 50nM Trametinib (T) from day four to day six post 4OHT. Cells were analysed on day six after induction with 4OHT. **a,** Representative images. Scale bar, 20 μm. **b,** Plots showing nuclear intensities per cell and mean value, n = 200 cells per condition. Representative data of one out of three independent experiments. Ordinary one-way ANOVA with Sidak's multiple comparisons test. **c,** Quantification of nuclear intensities of the indicated splicing factors in IMR90 E6/E7 cells expressing RAS (IMR90 E6/E7 ER:RAS + 4OHT, indicated by R) and their corresponding controls (IMR90 E6/E7 vector + 4OHT, V). Single-cell nuclear intensities and mean values, n = 200 cells per condition. Representative data of one out of three independent experiments. Unpaired t-test. (**d-e**) Immunofluorescence staining of different splicing factors in control IMR90 cells (vector +4OHT, indicated by V) or IMR90 ER:RAS cells undergoing senescence (IMR90 ER:RAS +4OHT, indicated by R) three days after treatment with 4OHT. **d,** Representative images. Scale bar, 200 μm. **e,** Plots showing single-cell nuclear intensities and mean values, n = 200 cells per condition. Representative data of one out of three independent experiments. Unpaired t-test, two-tailed. **f,** Quantification of nuclear intensities of splicing factors SF3B4 and DHX15 in IMR90 cells expressing the indicated HRAS mutant or their corresponding vector (V) controls. Plots showing single-cell nuclear intensities and mean values, n = 200 cells per condition. Representative data of one out of three independent experiments. Ordinary one-way ANOVA with Sidak's multiple comparisons test. **g,** Representative images of immunofluorescence against BrdU in the indicated cells. **h**, Quantification of the percentage of BrdU-positive cells in the indicated cells. V, vector; R, RAS:ER; G, growing; Q, quiescence. All cells were treated with 4OHT. Mean and standard deviation, n = 3 independent experiments. Ordinary one-way ANOVA with Sidak's multiple comparisons test. ns, non-significant. Source data are provided as a Source Data file.

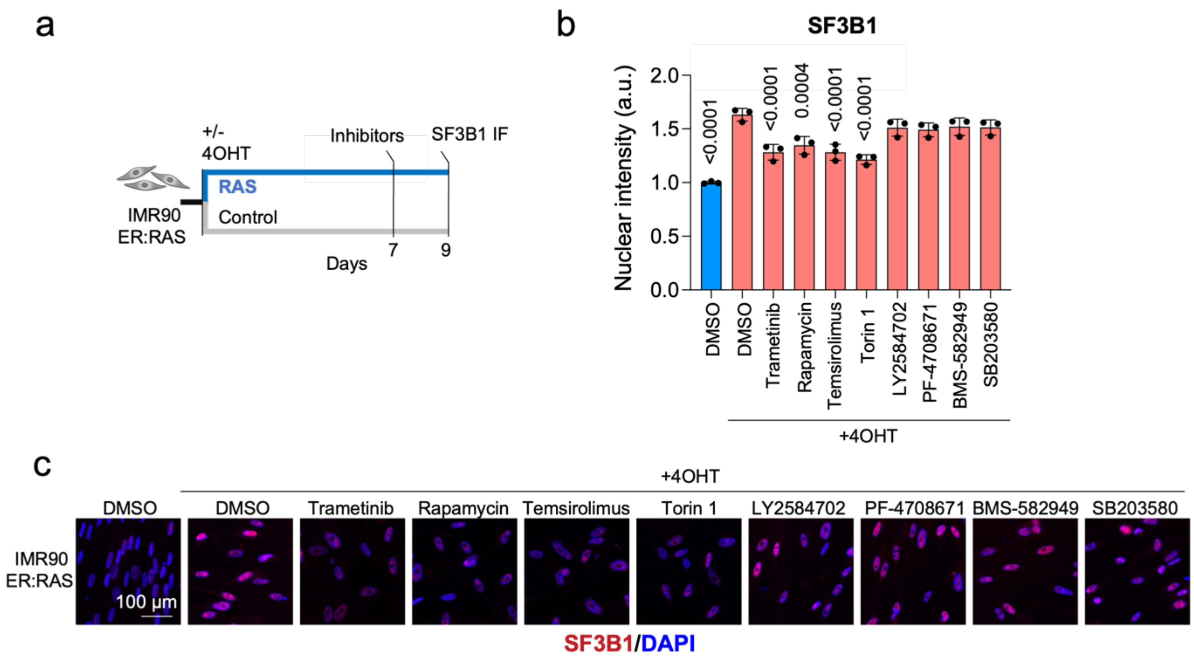

Supplementary Figure 6. mTOR inhibition prevents RAS^{V12} induction of splicing factors. Related to Figure 2. a. Scheme of the experimental design. IMR90 ER:RAS cells were induced for seven days with either DMSO (control) or 4OHT (senescent cells), then treated with the different inhibitors for two days before SF3B1 immunostaining. **b-c,** Quantification (**b**) and representative images (**c**) of SF3B1 nuclear immunostaining. Scale bar, 100 µm. Ordinary one-way ANOVA with Dunnett's multiple comparisons test to the untreated senescent samples (second from left). n = 3 independent experiments. Source data are provided as a Source Data file.

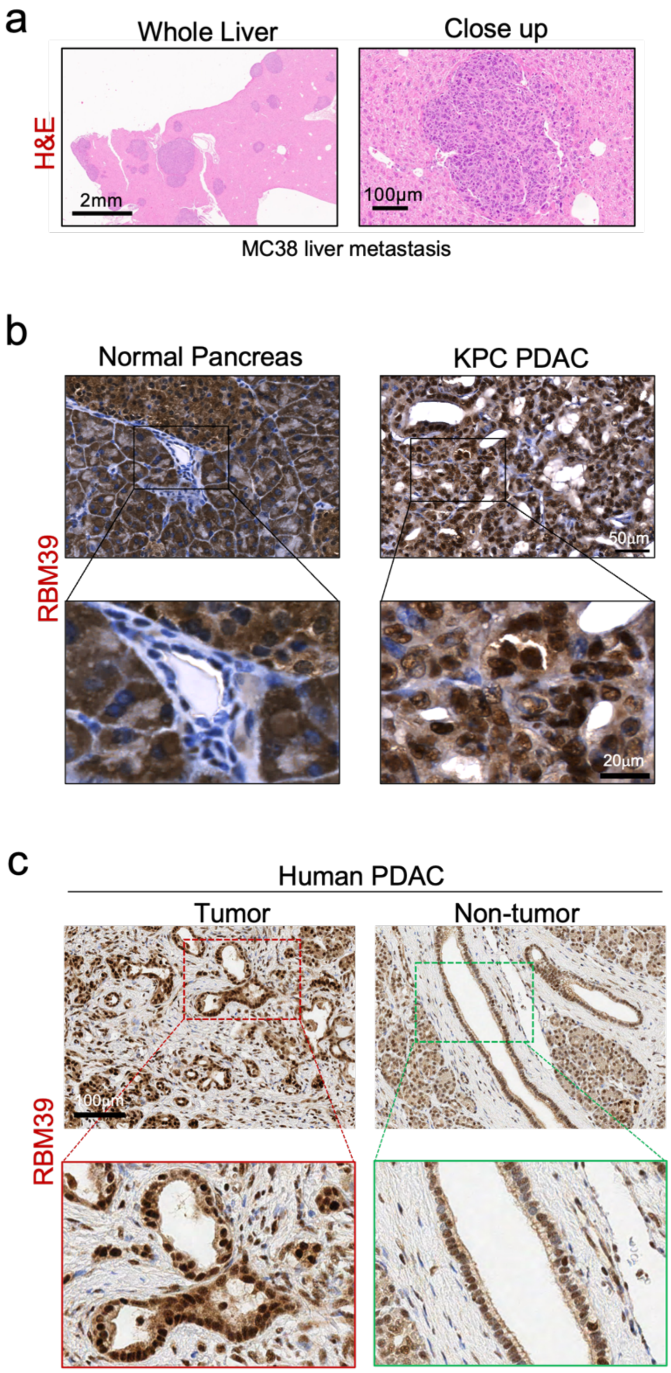

Supplementary Figure 7. High expression of SF3B1 and RBM39 in tumours expressing oncogenic RAS. Related to Figure 3. a, H&E-stained liver sections corresponding to the MC38 KRAS-mutant colorectal cancer liver metastasis are shown in Figure 3e. Scale bars 2 mm and 100 µm. **b,** Expression of RBM39 in the murine pancreas (n = 5) and murine pancreatic ductal adenocarcinomas (PDAC) of KPC mice (*LSL-Kras*^{G12D}*/+; LSL-Trp53*$^{R172H/+}$*; Pdx1-Cre*; n = 6). Scale bars 50 µm and 20 µm. Quantification is shown in Figure 3j. **c,** Representative images of pancreas sections derived from PDAC patients (n = 12) showing staining of RBM39 in tumour (T) and non-tumour (NT) sections. Scale bar, 100 µm. Quantification is shown in Figure 3l.

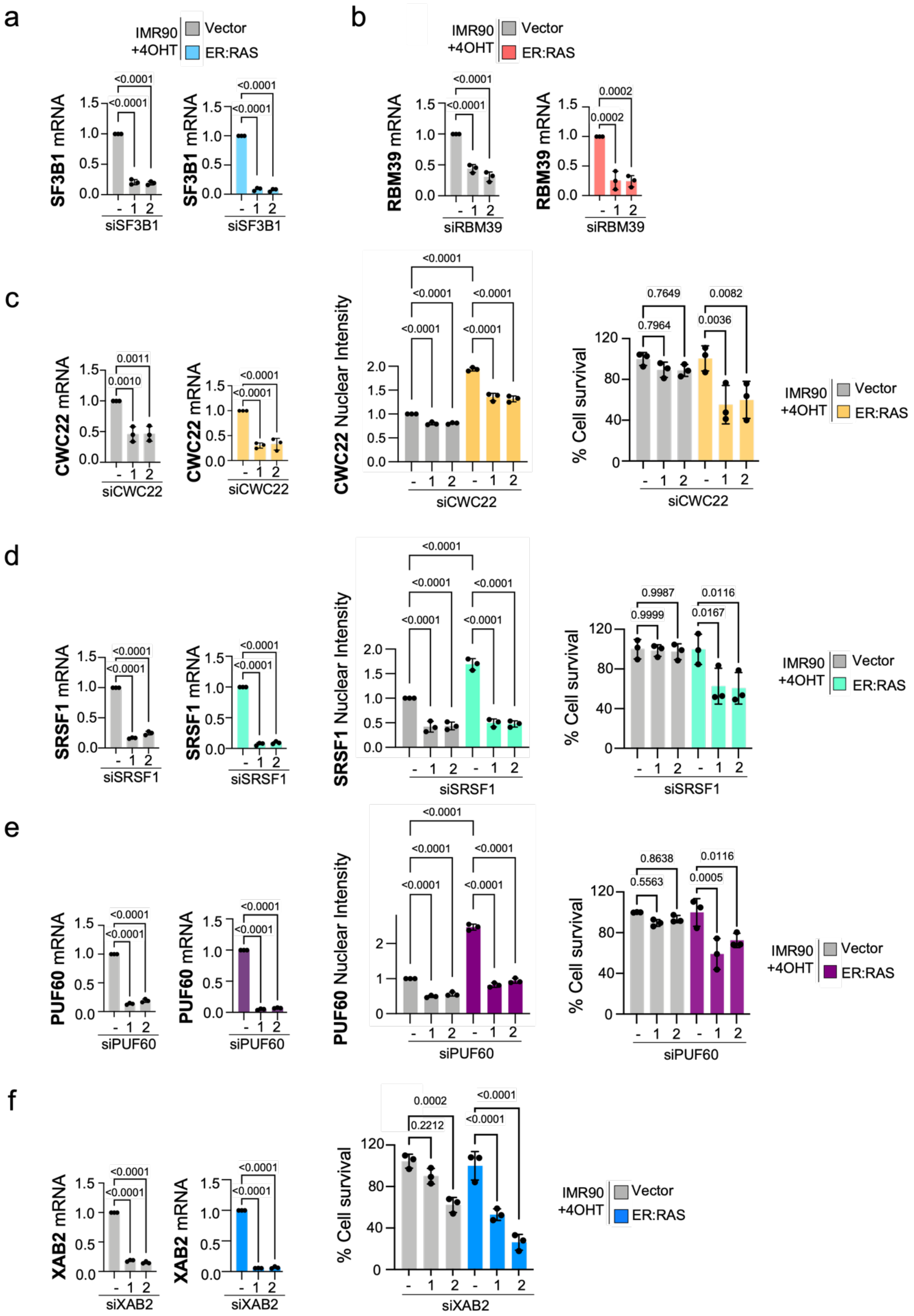

Supplementary Figure 8. The spliceosome is a vulnerability of cells undergoing RAS-induced senescence. Related to Figure 4. a-b, Knockdown of SF3B1 (A) and RBM39 (B)in IMR90 ER:RAS (coloured) or IMR90 vector control (grey) cells. Cells were

transfected with non-targeting siRNAs (siNT, -) or siRNAs targeting SF3B1 (1, siSF3B1.1; 2, siSF3B1.2; **a**) or RBM39 (1, siRBM39.1; 2, siRBM39.2; **b**). mRNA expression of SF3B1 (**a**) or RBM39 9 (**b**) was measured two days after transfection. Individual values, mean, and standard deviation. n = 3 independent experiments. Ordinary one-way ANOVA with Dunnett's multiple comparisons test. ns = not significant, **c-e,** Knockdown of CWC22 (**c**), SRSF1 (**d**), or PUF60 (**e**) in IMR90 cells undergoing RAS-induced senescence (coloured) and their corresponding controls (grey). Cells were transfected with 2 different siRNAs (indicated as 1 or 2) targeting CWC22 (**c**), SRSF1 (**d**), PUF60 (**e**), or non-targeting siRNAs (indicated as -). mRNA expression (left panel, 2 days after siRNA transfection), nuclear expression of the corresponding splicing factors (middle panels, 3 days after siRNA transfection), or cell survival (right panels, 6 days after siRNA transfection). Individual values, mean, and standard deviation. n = 3 independent experiments. Ordinary one-way ANOVA with Dunnett's or Sidak's multiple comparisons test, respectively. ns, not significant. **f,** Knockdown of XAB22 in IMR90 cells undergoing RAS-induced senescence (coloured) and their corresponding controls (grey). Cells were transfected with 2 different siRNAs (indicated as 1 or 2) targeting XAB2, or non-targeting siRNAs (indicated as -). mRNA expression (left panel, two days after siRNA transfection), or cell survival 6 days after siRNA transfection (right panels). Individual values, mean, and standard deviation. n = 3 independent experiments. Ordinary one-way ANOVA with Sidak's multiple comparisons test. Source data are provided as a Source Data file.

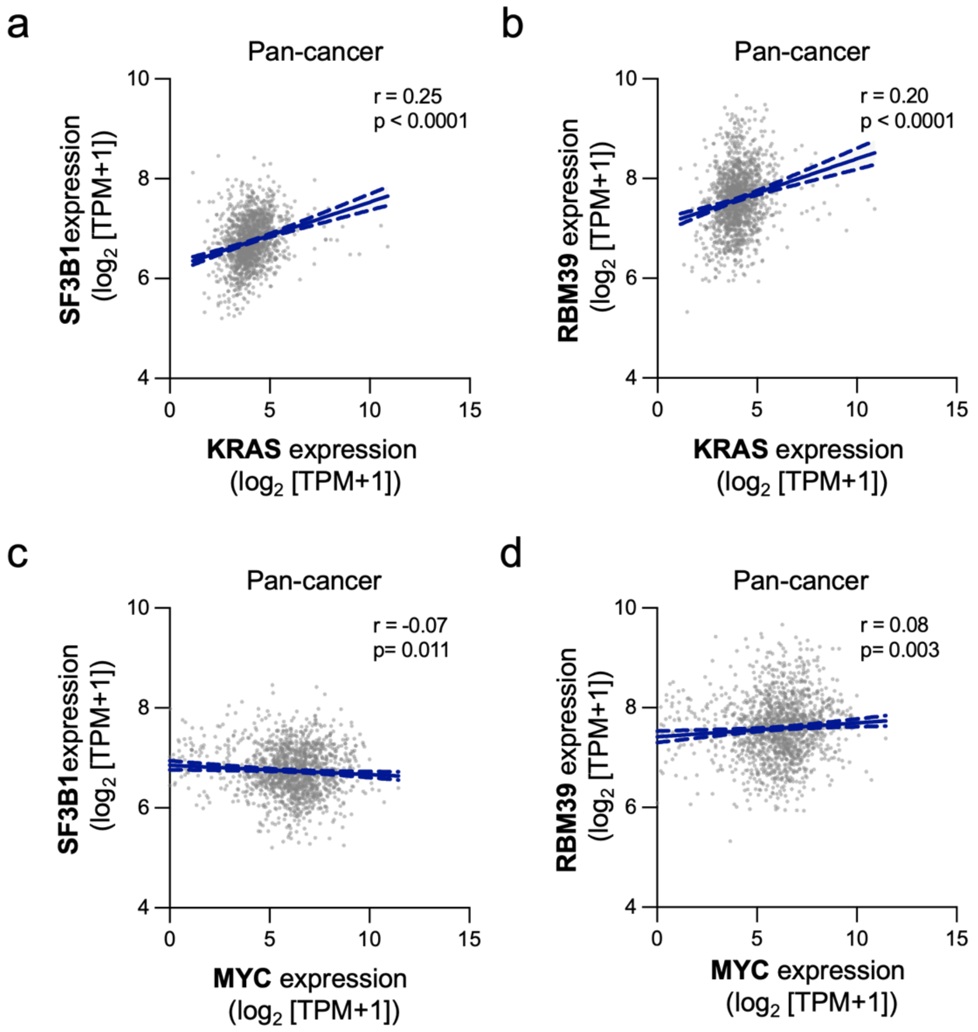

Supplementary Figure 9. KRAS, but not MYC expression, correlates with SF3B1 and RBM39 expression across cancer cell lines. Related to Figure 4. Positive correlation between expression of KRAS (**a-b**) or MYC (**c-d**) and SF3B1 (**a**, **c**) or RBM39 (**b**, **d**) in human cancer samples (pan-cancer analysis). Data were retrieved from the dependency map database (https://depmap.org/portal/). Simple linear regression and 95% CI (dotted lines). n = 1479 cell lines. Pearson r and p-value as indicated. Source data are provided as a Source Data file.

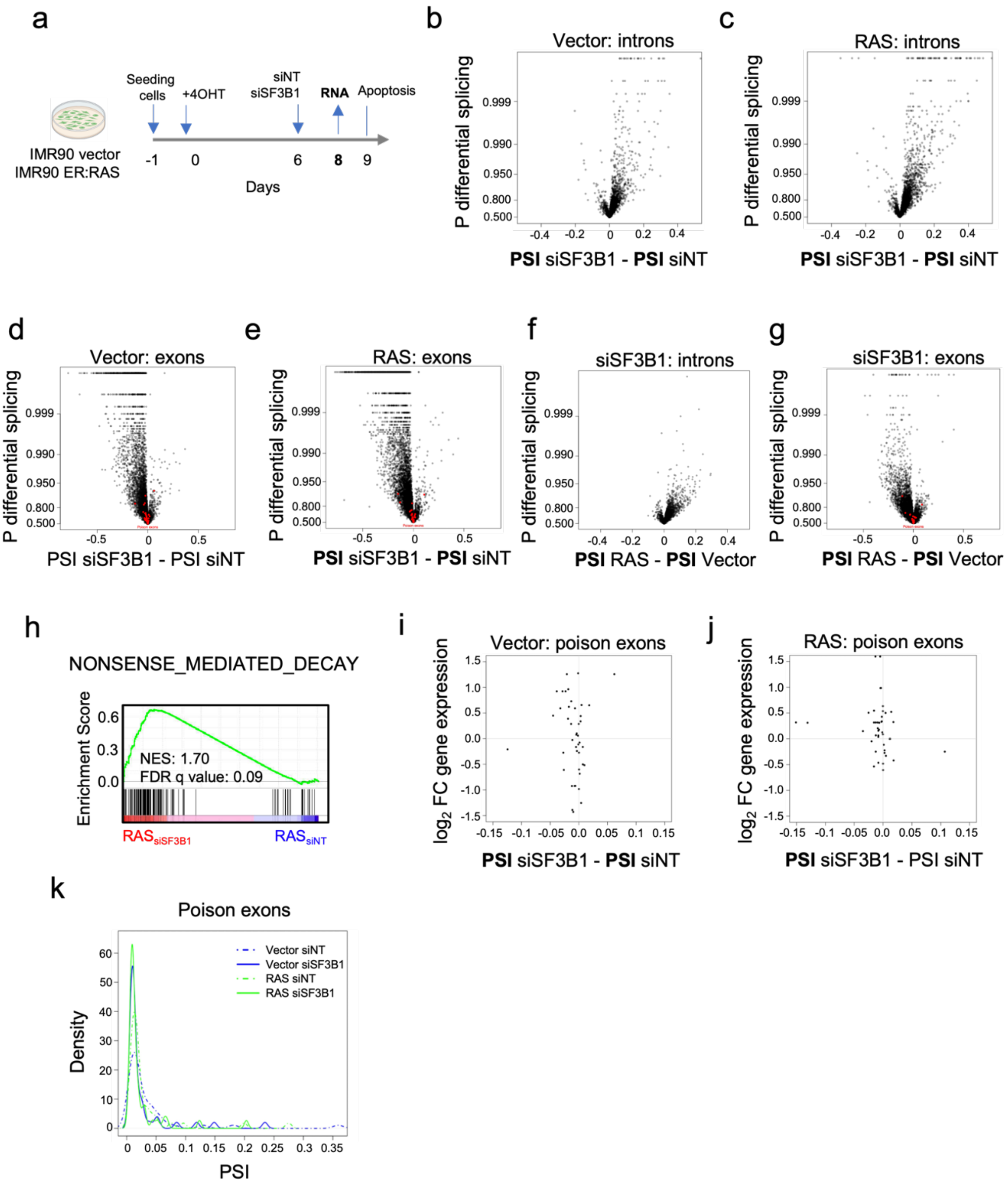

Supplementary Figure 10. SF3B1 is required to maintain efficient splicing in cells expressing oncogenic RAS. Related to Figure 5. a, Design of RNA-Seq experiment. Cells expressing oncogenic RAS (IMR90 ER:RAS) or their corresponding controls were treated with 4OHT (to activate RAS expression) and transfected with siRNAs targeting SF3B1 or non-targeting controls (siNT). RNA was collected 48 hours after transfection, at a point where significant knockdown of SF3B1 was observed, but cell death caused by SF3B1 knockdown in cells expressing oncogenic RAS was not yet apparent. Created in BioRender. Wagner, V. (2025) https://BioRender.com/ut56gut. **b-e,** Volcano plots of differential splicing (ΔPSI on X-axis, probability of differential splicing on log Y-axis) in IMR90 ER: RAS (RAS) or IMR90 control cells (IMR90 vector +4OHT, Vector) transfected

with siRNAs against siSF3B1 compared to non-targeting siRNAs (siNT). **b-c,** Retained introns. **d-e,** “cassette” exons (black) and poison exons (red). Pooled results of two different siRNAs, respectively. **f-g,** Volcano plots of differential splicing (retained introns in **f**, “cassette” exons (black), and poison exons (red) in **g**) in cells expressing oncogenic RAS after knockdown of SF3B1 (siSF3B1) compared to control cells. **h,** Gene set enrichment analysis (GSEA) shows the upregulation of genes involved in nonsense-mediated decay in IMR90 RAS cells transfected with siSF3B1 compared to cells transfected with a non-targeting siRNA (siNT). NES, normalised enrichment score; FDR, false discovery rate. **i-j**, Scatter plots showing changes in inclusion levels (ΔPSI, X-axis) of poison exons and changes in expression of their cognate genes ($\log_2$ fold-change, Y-axis) after knockdown of SF3B1 (siSF3B1 vs. siNT) in IMR90 cells expressing oncogenic RAS (**j**) and control cells (**i**)**. k,** Density plots (i.e., smoothed histograms) show the distribution of average inclusion levels (average PSI) of the analysed 43 poison exons in the four experimental conditions.

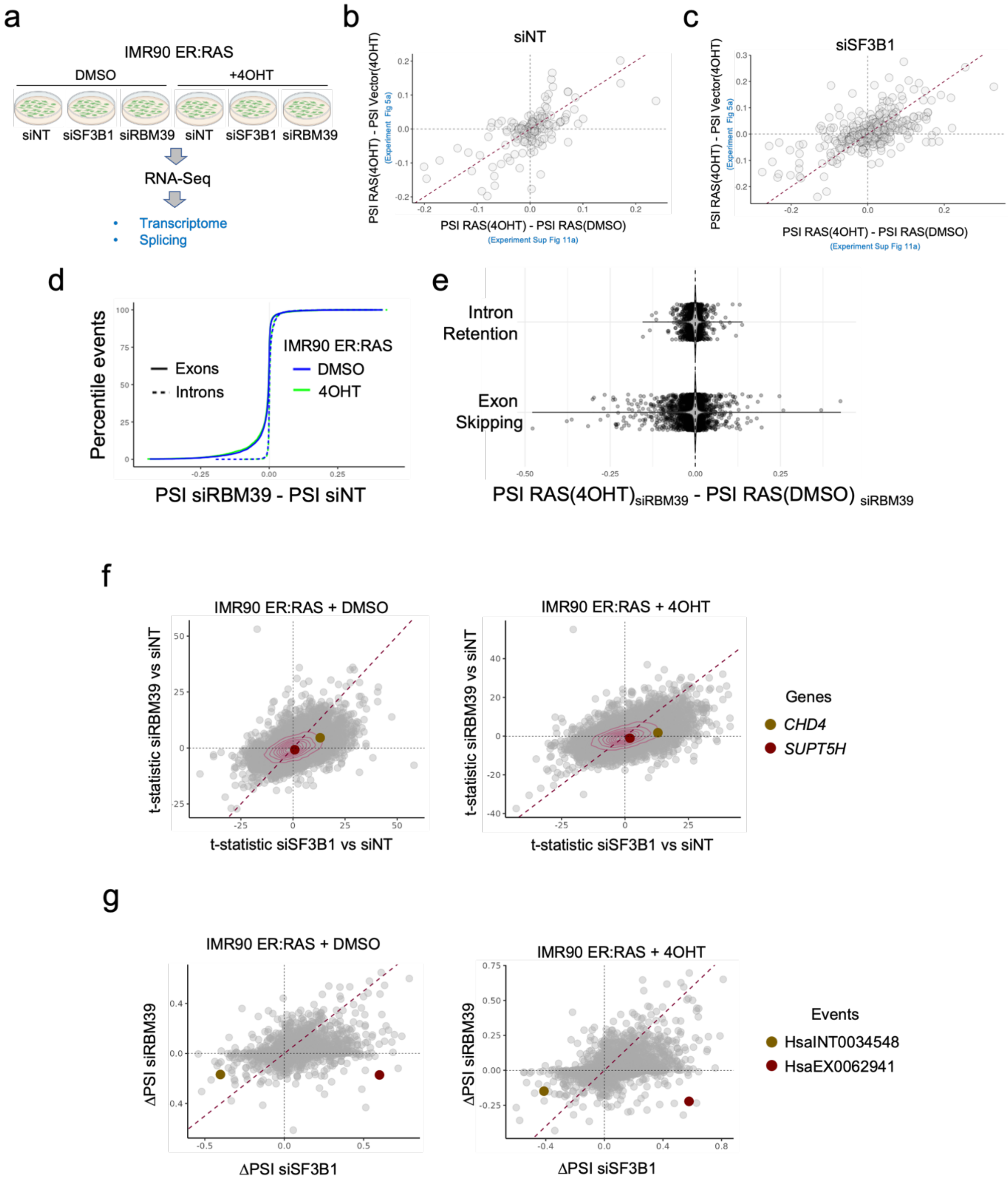

Supplementary Figure 11. SF3B1 and RBM39 knockdowns affect the alternative splicing of different subsets of genes. Related to Figure 5. a, RNA-sequencing analysis to investigate how SF3B1 and RBM39 knockdowns affect IMR90 cells expressing oncogenic RAS (IMR90 ER:RAS +4OHT) compared with the non-induced cells (IMR90 ER:RAS +DMSO). Experimental design. Cells were reversely transfected with siRNAs against SF3B1 (siSF3B1), RBM39 (siRBM39), or non-targeting siRNAs (siNT) on day 6 after treatment with 4OHT. Created in BioRender. Wagner, V. (2025) https://BioRender.com/ut56gut. **b-c,** Correlation plots showing the reproducibility of the detection of the alternative splicing events (measured by ΔPSI) between the SF3B1

knockdown experiment presented in Figure 5a and the new SF3B1 and RBM39 knockdown experiment presented here (Sup Fig 11a). Correlation of the effect of siNT (**b**) and siSF3B1 (**c**) for the two experiments. Dashed lines represent the identity line (Figure 5a ΔPSI = Sup Fig 11a ΔPSI), Sup Fig 11a ΔPSI = 0 and Figure 5a ΔPSI = 0. **d,** Empirical cumulative distributions of differences in percent spliced in index (PSI) between siRBM39 and siNT for IMR90 ER:RAS induced with RAS (4OHT, in green) and non-induced (DMSO, in blue) for 10,364 "cassette" exons (solid lines) and 10,374 retained introns (dashed lines) analysed. **e,** Distributions of ΔPSI of exon skipping and intron retention events in RAS-induced vs not induced IMR90 ER:RAS cells transfected with siRNAs targeting RBM39. **f–g**, Correlation of the effects of SF3B1 and RBM39 knockdowns on gene expression **(f)** and alternative splicing **(g)** in IMR90 ER:RAS cells treated with DMSO (left) or 4OHT (right). In **f**, gene-level t-statistics are shown for siSF3B1 vs siNT (x-axis) and siRBM39 vs siNT (y-axis). In **g**, splicing-level ΔPSI values are shown for siSF3B1 vs siNT (x-axis) and siRBM39 vs siNT (y-axis). *CHD4* and *SUPT5H* (genes in **f** and respective relevant alternative splicing events in **g**) are highlighted in yellow and dark pink, respectively. Dashed lines indicate the identity line (y = x, dark red) and reference lines at x = 0 and y = 0 (black). In **g**, the highlighted alternative splicing events had been filtered out during pre-processing and were added back afterwards for visualisation purposes.

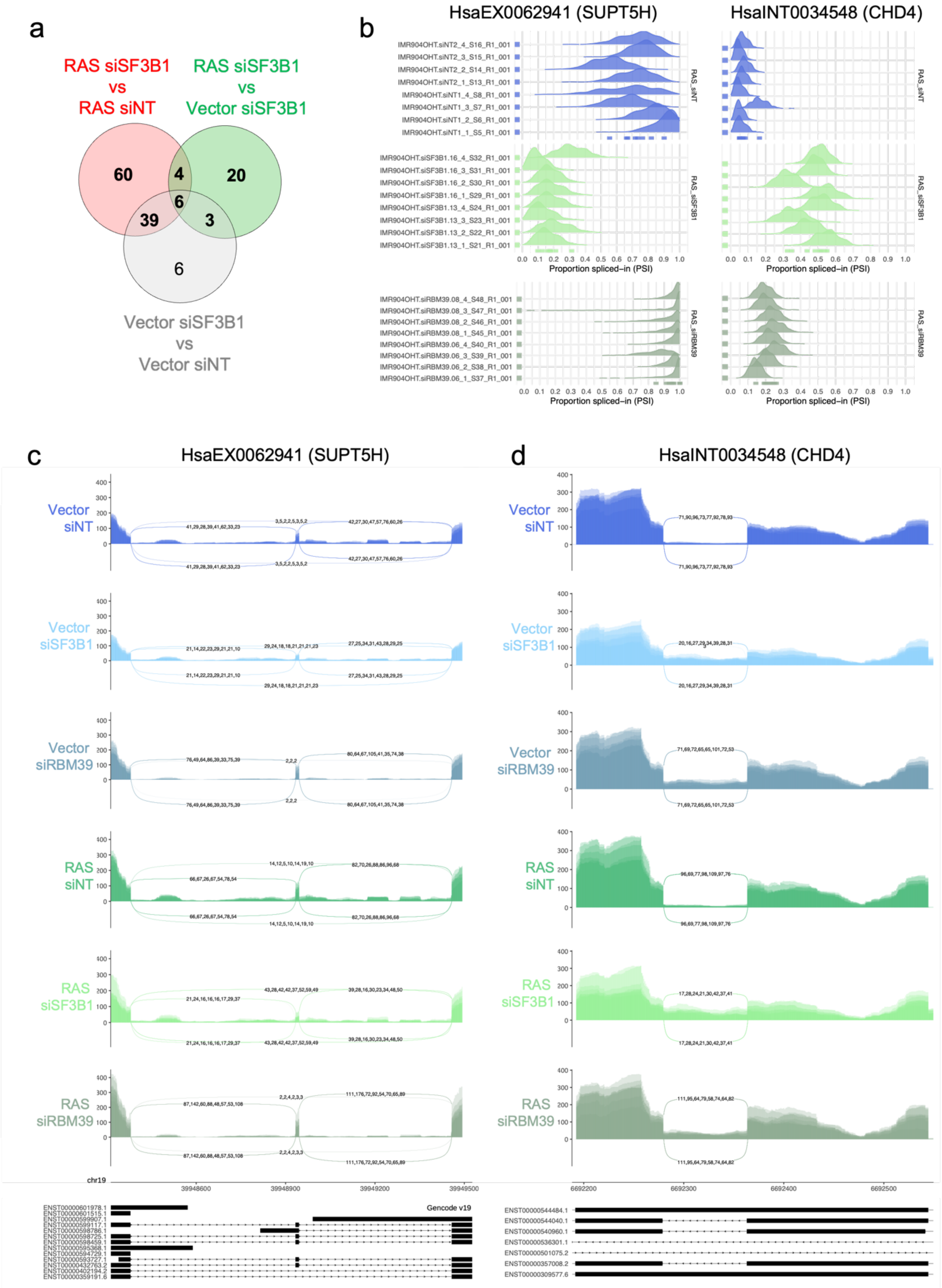

Supplementary Figure 12. The alternative splicing of SF3B1 targets, SPT5 and CHD4, is not modulated by the downregulation of RBM39. Related to Figure 5. a, Selection of genes further evaluated as potential downstream effectors of SF3B1 knockdown-mediated cell death. Genes differentially spliced and differentially

expressed/downregulated in IMR90 RAS cells transfected with siSF3B1, compared to IMR90 RAS cells transfected with siNT, or compared to IMR90 control cells transfected with siSF3B1, were considered. 132 genes fulfilled the criteria. **b,** Probability distributions (density plots of randomly emitted values from beta distributions) and vast-tools PSIs (bottom coloured ticks) for the relevant *SUPT5H* (HsaEX0062941, left column) and *CHD4* (HsaINT0034548, right column) alternative splicing events in cells treated with 4OHT (experimental setup described in Supplementary Figure 11a). Each row corresponds to a different condition: control (siNT, blue), SF3B1 knockdown (siSF3B1, light green), and RBM39 knockdown (siRBM39, dark green). **c-d,** Sashimi plots representing read coverage and splice junctions across all the conditions of the experimental setup described in Supplementary Figure 11a, for the microexon event in *SUPTH5*, HsaEX0062941 (**c**), and the intron retention event in *CHD4*, HsaINT0034548 (**d**). Each panel displays RNA-seq data for a specific condition, with read coverage (y-axis) and exon-exon junctions (arcs labelled with supporting read counts). Ensembl transcript structure in each event's genomic region is shown below.

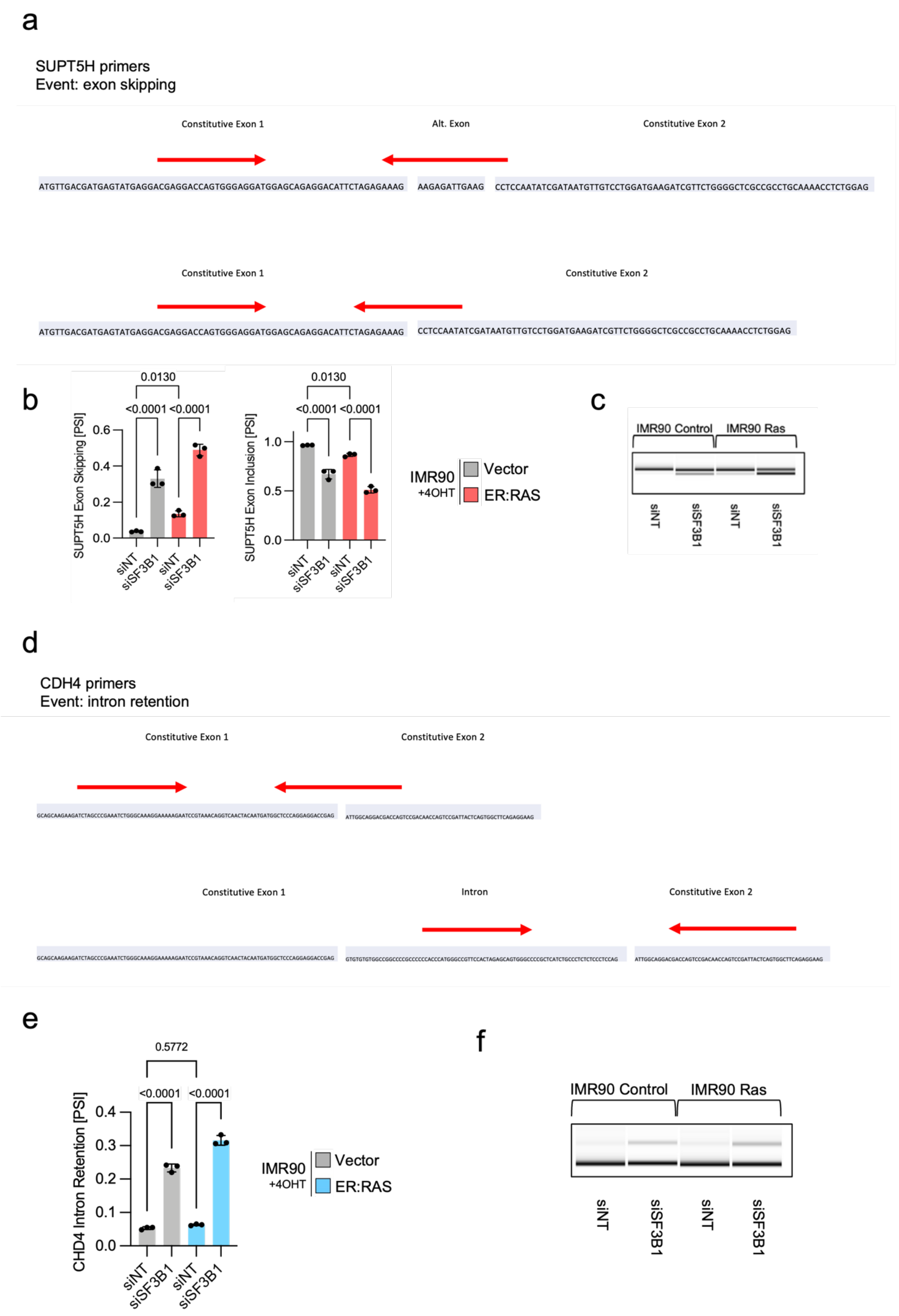

Supplementary Figure 13. Effect of SF3B1 knockdown on SUPT5H and CDH4 splicing. Related to Figure 5. a, Design of primers to detect alternative splicing of SUPT5H (microexon

HsaEX0062941): one primer was placed either across the exon–exon junction (without microexon) or directly on the microexon, ensuring that each primer pair amplified only one of the two alternatively spliced transcripts. **b**, Quantification of SUPT5H exon skipping/inclusion (microexon HsaEX0062941). RT-qPCR with absolute quantification of each transcript (for details see Materials and Methods, "cDNA synthesis and RT-PCR”). Increased exon skipping (and decreased exon inclusion) in both RAS-expressing cells (IMR90 RAS, +4OHT) and vector-expressing cells (IMR90 control, +4OHT) upon knockdown of SF3B1 compared to non-targeting (siNT). Individual values, mean, and standard deviation. n = 3 independent experiments. Ordinary one-way ANOVA with Sidak's multiple comparisons test. **c**, RT-PCR with a third primer pair detecting both alternatively spliced transcripts (with and without microexon) and consecutive microfluidic electrophoresis (Agilent 2100 Bioanalyzer), confirming increased exon skipping. This is reflected by a higher abundance of the smaller isoform (lower band), predominantly in RAS-expressing cells (IMR90 RAS, +4OHT). **d**, Design of primers to detect alternative splicing of CHD4 (HsaINT0034548): one primer was placed either across the exon–exon junction (without intron) or directly on the intron, ensuring that each primer pair amplified only one of the two alternatively spliced transcripts. **e**, Quantification of alternative splicing of CHD4 (intron HsaINT0034548) by RT-qPCR showing increased intron retention upon knockdown of SF3B1 in RAS-expressing cells (IMR90 RAS, +4OHT) and vector-expressing cells (IMR90 control, +4OHT). n = 3 independent experiments. Ordinary one-way ANOVA with Sidak's multiple comparisons test. **f**, RT-PCR and subsequent microfluidic electrophoresis (Agilent 2100 Bioanalyzer) using a primer pair detecting both isoforms, confirming the increase in intron retention upon knockdown of SF3B1. Source data are provided as a Source Data file. Uncropped images of gels are shown at the end of the Supplementary Information.

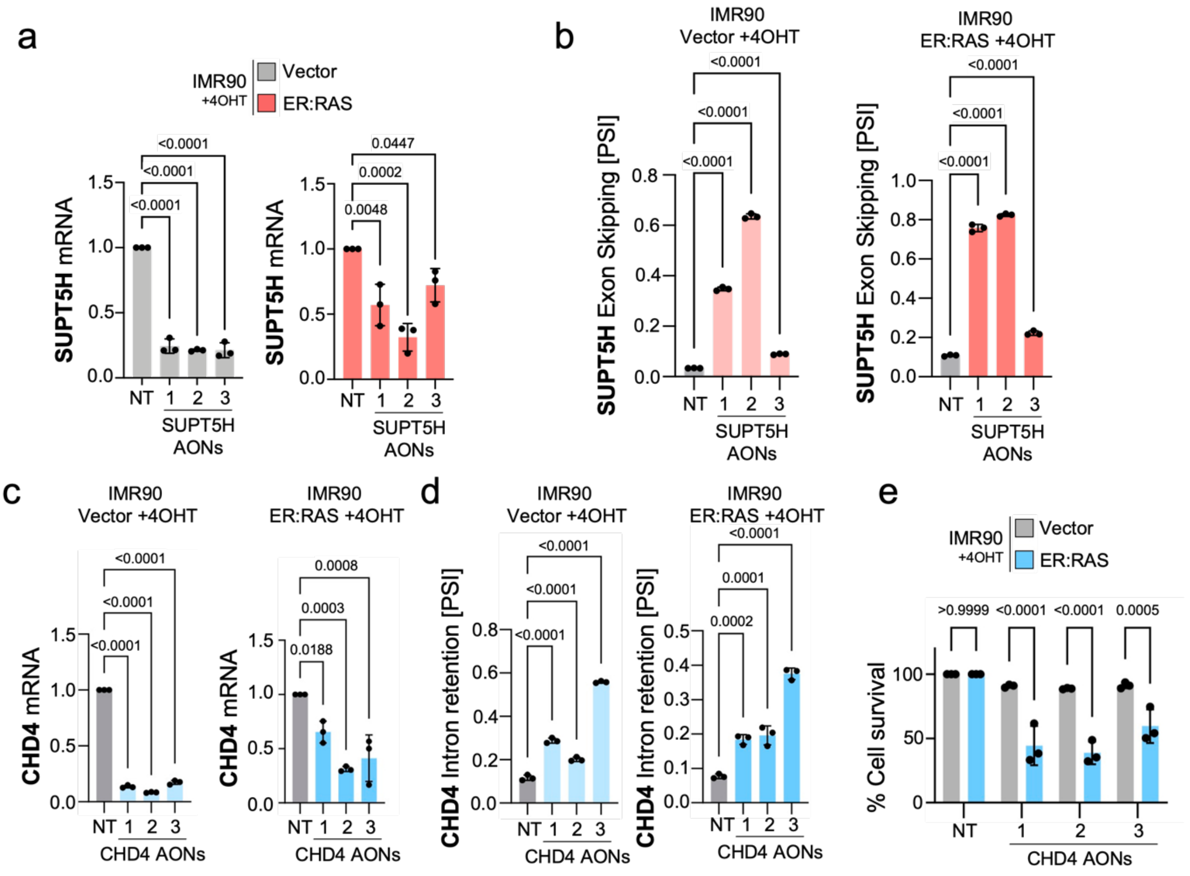

Supplementary Figure 14. Transcription factor SPT5 is a therapeutic vulnerability of cells expressing oncogenic RAS. Related to Figure 5. a-b, Effect of AONs (antisens oligonucleotides) targeting SUPT5H. Transfection of cells with AONs targeting the splicing of SUPT5H significantly reduces the amount of total SUPT5H mRNA **(a)** and leads to increased exon skipping (microexon HsaEX0062941) in both RAS-expressing cells (IMR90 RAS + 4OHT) and vector-expressing cells (IMR90 vector + 4OHT) **(b)**. These experiments relate to Figure 5k. Individual values, mean, and standard deviation. n = 3 independent experiments. One-way ANOVA with Dunnett's multiple comparisons test. **c-d,** Effect of AONs targeting CHD4. Total mRNA levels (**c**) and intron retention (intron HsaINT0034548) (**d**). Individual values, mean, and standard deviation. n = 3 independent experiments. One-way ANOVA with Dunnett's multiple comparisons test. **e,** Transfection of AONs targeting the splicing of CHD4 selectively affect the viability of RAS-expressing cells (IMR90 RAS + 4OHT) compared to control cells (IMR90 vector +4OHT). Individual values, mean, and standard deviation. n = 3 independent experiments. ns = not significant; Ordinary two-way ANOVA with Sidak's multiple comparisons test. For details of quantification of alternative splicing see Materials and Methods "cDNA synthesis and RT-PCR" and Supplementary Figure 13. Source data are provided as a Source Data file.

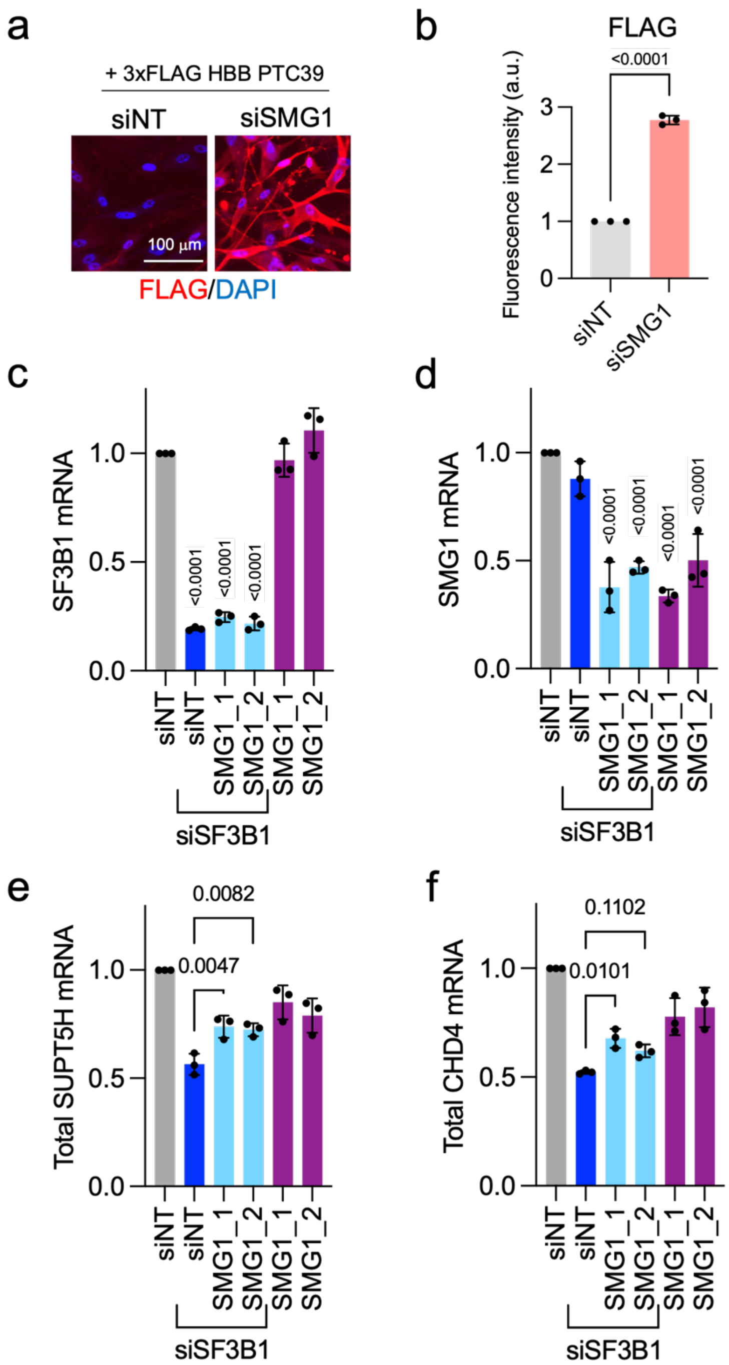

Supplementary Figure 15. Nonsense-mediated decay (NMD) is involved in the downregulation of SPT5 and CHD4. Related to Figure 5. a-b, IMR90 ER:RAS cells overexpressing 3xFLAG HBB PTC39 were transfected with non-targeting siRNAs (siNT) or siRNAs against SMG1 on day six after addition of 4OHT. After another four days, cells were fixed and immunostained (**a**) to quantify the cellular intensity of the 3xFLAG HBB PTC39 construct (**b**). Individual values, mean, and standard deviation. n = 3 independent experiments. Unpaired t-test, two-tailed. **c-f**, IMR90 ER:RAS cells were transfected with either control siRNAs (siNT) or siRNAs against SMG1 on day six. After two days, they were transfected with siRNAs against SF3B1 or siNT. After another two days, RNA was collected, and the amount of RNA of the respective transcripts was quantified by RT-qPCR.

c, SF3B1 mRNA fold change. **d,** SMG1 mRNA fold change. Individual values, mean, and standard deviation. n = 3 independent experiments. Ordinary one-way ANOVA with Dunnett's multiple comparisons test. **e,** mRNA fold change of SUPT5H. **f,** mRNA fold change of CHD4. Individual values, mean, and standard deviation. n = 3 independent experiments. Ordinary one-way ANOVA with Sidak's multiple comparisons test. Source data are provided as a Source Data file.

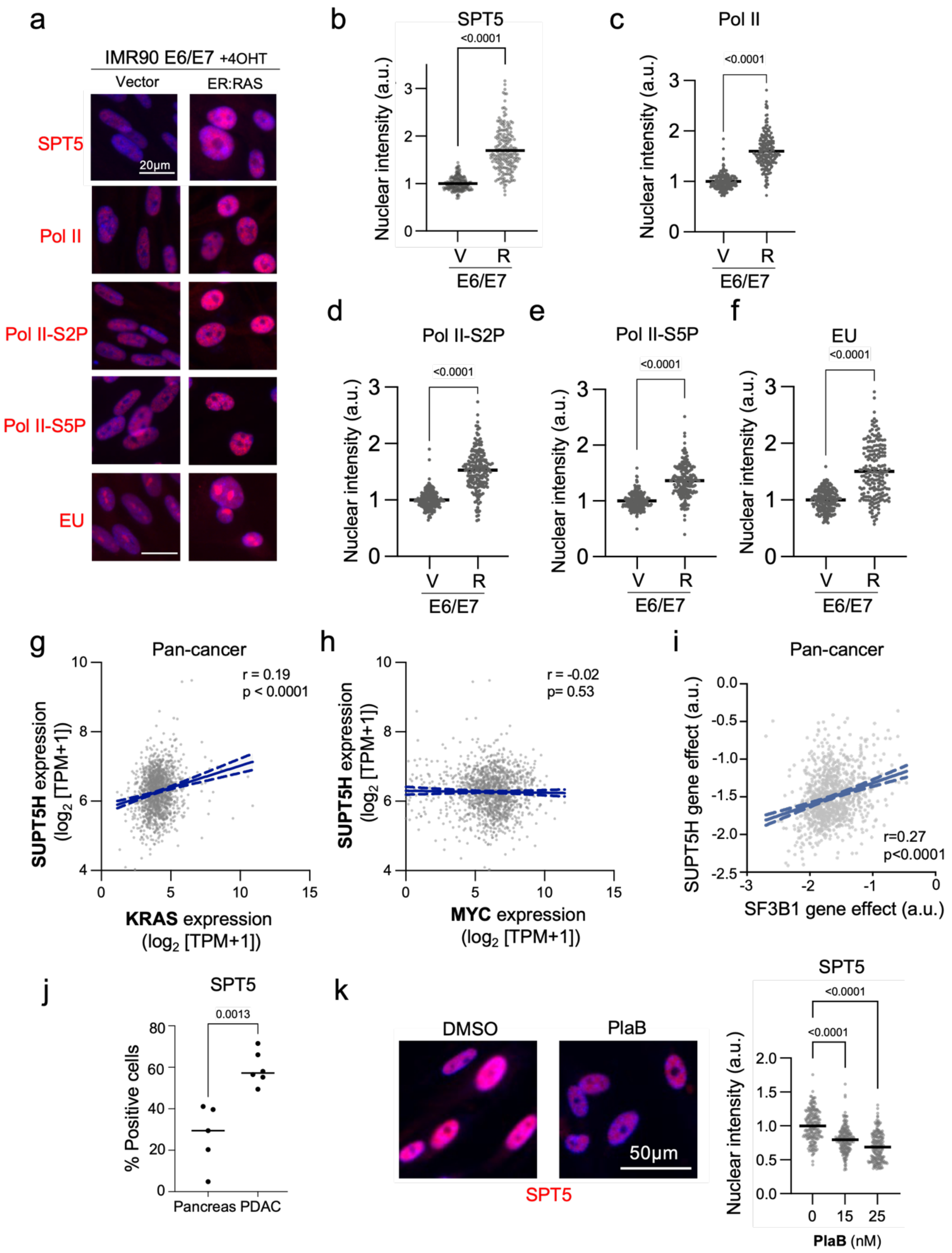

Supplementary Figure 16. Relation between RAS induction, SF3B1, and SPT5 levels. Related to Figure 6. Representative images (**a**) and quantification (**b-f**) of SPT5 (**b**), total levels of RNA polymerase II (Pol II, **c**), RNA polymerase II phosphorylated at S2 of the carboxy-terminal domain (CTD; Pol II-S2P, **d**), RNA polymerase II phosphorylated at S5 of the CTD (Pol II-S5P, **e**), and newly synthesized RNA/incorporated EU (**f**), in IMR90 E6/E7 vector cells (V) and IMR90 E6/E7 ER:RAS cells treated with 4OHT (R). Scale bar, 20 μm.

Plot showing single-cell nuclear intensities and mean values for n = 200 cells per condition. 1 out of 3 independent experiments. Unpaired t-test, two-tailed. **g-h,** Positive correlation between expression of KRAS (**g**) or MYC (**h**) and SUPT5H in human cancer samples (pan-cancer analysis). Data were retrieved from the dependency map database (https://depmap.org/portal/). Simple linear regression and 95% CI (dotted lines). Pearson r and p-value as indicated. **i,** Positive correlation between the effect of SF3B1 and SUPT5H knockout in human cancer samples (pan-cancer analysis, CRISPR (DepMap 21Q4 Public+Score; Chronos). Data were retrieved from the Dependency Map database (https://depmap.org/portal/). Simple linear regression and 95% CI (dotted lines). Pearson r and p-value as indicated. **j,** Expression of SPT5 in the murine pancreas (n = 5) and murine pancreatic ductal adenocarcinomas (PDAC) of KPC mice (*LSL-Kras*$^{G12D/+}$*; LSL-Trp53*$^{R172H/+}$*; Pdx1-Cre*) (n = 6). Individual and mean values for the percentage of positive cells are plotted. Unpaired t-test, two-tailed. **k,** Representative images (left) and quantification (right) of SPT5 protein expression in IMR90 ER:RAS cells treated with DMSO or the indicated concentrations of Pladienolide B (PlaB) six days after 4OHT induction. Cells were fixed 24 hours after treatment. Scale bar, 20 μm. The graph shows nuclear intensities and mean values for n = 200 cells per condition. 1 out of 3 independent experiments. Unpaired t-test, two-tailed. Source data are provided as a Source Data file.

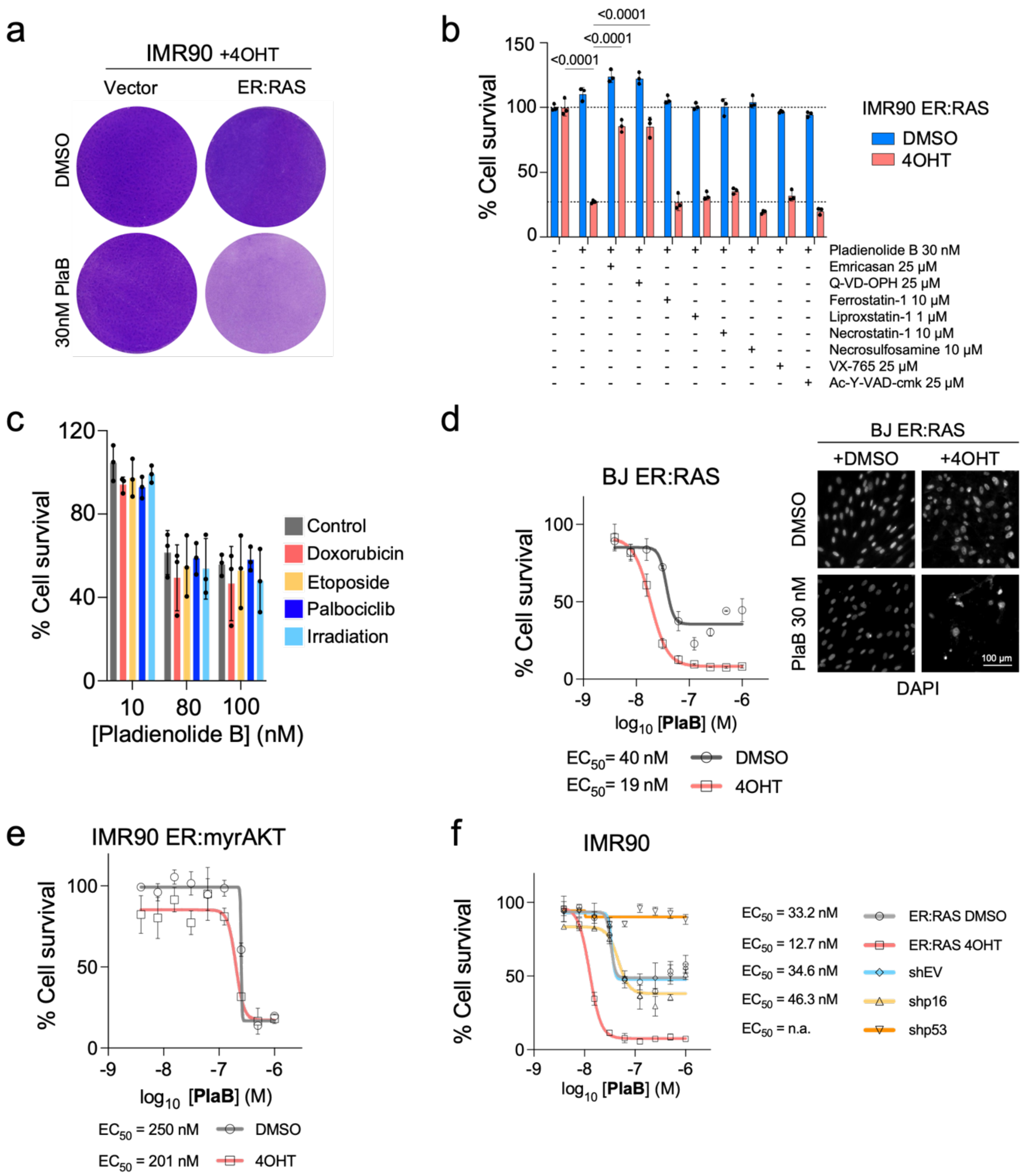

Supplementary Figure 17. The SF3B1 inhibitor PlaB induces apoptosis in cells expressing oncogenic RAS. Related to Figure 7. a, Crystal violet staining of the indicated cells treated with DMSO or 30nM Pladienolide B at day 6 after induction with 4OHT. The plates were fixed four days later. Representative images. **b,** Rescue experiment of the RAS^{V12}-expressing cells death induced by the treatment with the SF3B1 inhibitor Pladienolide B. IMR90 ER:RAS cells were induced for six days with 4OHT (or DMSO for the control cells), then treated with either 30 nM of Pladienolide B alone or in combination with apoptosis (pan-caspase) inhibitors (Emricasan and Q-VD-OPh, 25 µM), ferroptosis inhibitors (Ferrostatin-1, 10 µM, and Liproxstatin-1, 1 µM), necroptosis inhibitors (RIPK1-targeted inhibitor Necrostatin-1, 10 µM, and MLKL-targeted inhibitor Necrosulfosamine, 10 µM) or pyroptosis (caspase 1) inhibitors (VX-765/Belnacasan, 25

μM, and Ac-Y-VAD-cmk, 25 μM) for another three days. Individual values, mean, and standard deviation. n = 3 independent experiments. Ordinary two-way ANOVA with Bonferroni's multiple comparisons test. **c,** Analysis of how the SF3B1 inhibitor Pladienolide B (PlaB) affects the survival of IMR90 cells undergoing senescence induced by doxorubicin, etoposide, Palbociclib, or γ-irradiation compared to controls. Mean and standard deviation, n = 3 independent experiments. Ordinary two-way ANOVA with Dunnett's multiple comparisons test. All comparisons are non-significant. **d-f,** Dose-response curves of BJ ER:RAS (**d**), and IMR90 ER:myrAKT (**e**) induced or not (DMSO) with 4OHT for six days, then treated with Pladienolide B (PlaB) for another four days. The right panels in (**d**) show representative DAPI staining images for BJ ER:RAS cells. Scale bar, 100 μm. **f,** Dose-response curves of IMR90 ER:RAS induced or not (DMSO) with 4OHT for six days, and IMR90 expressing shRNAs against p16 or p53 (empty vector, control) were treated with Pladienolide B (PlaB) for four days. Dots represent the mean of 3 replicates +/- SD. EC_{50} values were calculated based on the nonlinear regression curves (least squares regression). Source data are provided as a Source Data file.

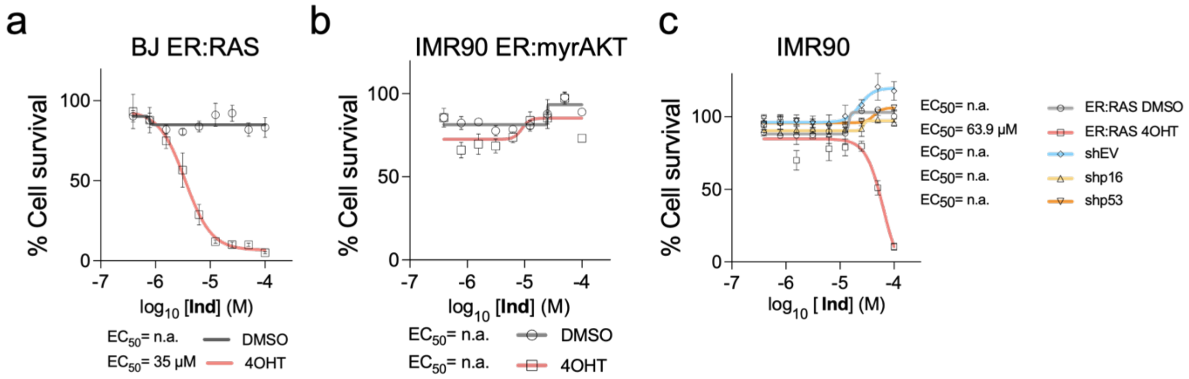

Supplementary Figure 18. The RBM39 degrader indisulam selectively eliminates RAS-expressing cells. Related to Figure 8. a-b, Dose-response curves of BJ ER:RAS (**a**), and IMR90 ER:myrAKT (**b**) induced or not (DMSO) with 4OHT for six days, then treated with indisulam (Ind) for another 4 days. **c,** Dose-response curves of IMR90 ER:RAS induced or not (DMSO) with 4OHT for six days, and IMR90 expressing shRNAs against p16 or p53 (empty vector, control) were treated with Indisulam (Ind) for four days. Dots represent the mean of 3 replicates +/- SD. EC_{50} values were calculated based on the nonlinear regression curves (least squares regression). Source data are provided as a Source Data file.

SUPPLEMENTARY TABLES

Supplementary Table 1. Primer sequences for mutagenesis

Name	Sequence reverse primer	Sequence forward primer
HRAS-G12A	TGCCCACACCGGCGGCGCCCACC	GGTGGGCGCCGCCGGTGTGGGCA
HRAS-G12C	CTTGCCCACACCGCAGGCGCCCACCACC	GGTGGTGGGCGCCTGCGGTGTGGGCAAG
HRAS-G12D	TGCCCACACCGTCGGCGCCCACC	GGTGGGCGCCGACGGTGTGGGCA
HRAS-G12S	CTTGCCCACACCGCTGGCGCCCACCACC	GGTGGTGGGCGCCAGCGGTGTGGGCAAG
HRAS-Q61R	CGCTGTACTCCTCCCTGCCGGCGGTATCCA	TGGATACCGCCGGCAGGGAGGAGTACAGCG

Supplementary Table 2. Sequence of 3xFLAG_NanoLuc_HBB PTC39

3xFLAG_NanoLuc_HBB PTC39	ctcgagATGGACTACAAAGACGATGACGACAAGGACTACAAAGACGATGA<br>CGACAAGGACTACAAAGACGATGACGACAAGGTCTTCACACTCGAAGA<br>TTTCGTTGGGGACTGGCGACAGACAGCCGGCTACAACCTGGACCAAGT<br>CCTTGAACAGGGAGGTGTGTCCAGTTTGTTTCAGAATCTCGGGGTGTC<br>CGTAACTCCGATCCAAAGGATTGTCCTGAGCGGTGAAAATGGGCTGAA<br>GATCGACATCCATGTCATCATCCCGTATGAAGGTCTGAGCGGCGACCA<br>AATGGGCCAGATCGAAAAAATTTTTAAGGTGGTGTACCCTGTGGATGAT<br>CATCACTTTAAGGTGATCCTGCACTATGGCACACTGGTAATCGACGGG<br>GTTACGCCGAACATGATCGACTATTTCGGACGGCCGTATGAAGGCATC<br>GCCGTGTTCGACGGCAAAAAGATCACTGTAACAGGGACCCTGTGGAAC<br>GGCAACAAAATTATCGACGAGCGCCTGATCAACCCCGACGGCTCCCTG<br>CTGTTCCGAGTAACCATCAACGGAGTGACCGGCTGGCGGCTGTGCGAA<br>CGCATTCTGGCGGTGCATCTGACTCCTGAGGAGAAGTCTGCCGTTACT<br>GCCCTGTGGGGCAAGGTGAACGTGGATGAAGTTGGTGGTGAGGCCCT<br>GGGCAGGCTGCTGGTGGTCTACCCTTGGACCTAGAGGTTCTTTGAGTC<br>CTTTGGGGATCTGTCCACTCCTGATGCTGTTATGGGCAACCCTAAGGTG<br>AAGGCTCATGGCAAGAAAGTGCTCGGTGCCTTTAGTGATGGCCTGGCT<br>CACCTGGACAACCTCAAGGGCACCTTTGCCACACTGAGTGAGCTGCAC<br>TGTGACAAGCTGCACGTGGATCCTGAGAACTTCAGGCTCCTGGGCAAC<br>GTGCTGGTCTGTGTGCTGGCCCATCACTTTGGCAAAGAATTCACCCCA<br>CCAGTGCAGGCTGCCTATCAGAAAGTGGTGGCTGGTGTGGCTAATGCC<br>CTGGCCCACAAGTATCACTAAtctaga

Supplementary Table 3. siRNAs

Oligonucleotides	Source	Catalogue number
siSF3B1	Dharmacon	J-020061-13, J-020061-16
siRBM39	Dharmacon	J-011965-06, J-011965-08
siCWC22	Dharmacon	J-023101-17, J-023101-18
siSRSF1	Dharmacon	J-018672-09, J-018672-11
siXAB2	Dharmacon	J-004914-09, J-004914-12
siPUF60	Dharmacon	J-012505-09, J-012505-11
siNT	Dharmacon	D-001810-01, D-001810-02
siSF3B4	Dharmacon	J-017190-05, J-017190-06, J-017190-07, J-017190-08
siDHX15	Dharmacon	J-011250-09, J-011250-10, J-011250-11, J-011250-12
siNHP2L1	Dharmacon	J-019900-05, J-019900-06, J-019900-07, J-019900-08
siSMG1	Dharmacon	J-005033-05, J-005033-07

Supplementary Table 4. Primer sequences and DNA templates

SUPT5H CON_ALT FOR (human)	TGACGATGAGTATGAGGACGAG
SUPT5H CON_ALT REV (human)	TGGAGGCTTCAATCTCTTCTTTC
SUPT5H CON_ALT template	AAGAATGACGATGAGTATGAGGACGAGGACCAGTGGGAGGATGGAGC AGAGGACATTCTAGAGAAAGAAGAGATTGAAGCCTCCAAAGAA
SUPT5H CON_CON FOR (human)	ATGAGTATGAGGACGAGGACCA
SUPT5H CON_CON REV (human)	TCGATATTGGAGGCTTTCTCTAGA
SUPT5H CON_CON template	AAGAAATGAGTATGAGGACGAGGACCAGTGGGAGGATGGAGCAGAGG ACATTCTAGAGAAAGCCTCCAATATCGAAAGAA
SUPT5H_TOTAL FOR (human)	AGAGGAGGAGGAAGAGGA
SUPT5H_TOTAL REV (human)	CACAGATGACTTGGCGTA
CHD4 ALT_CON FOR (human)	CATGGGCCGTTCCACTAGAG
CHD4 ALT_CON REV (human)	TTCATCACCTTCCTCTGAAGCC
CHD4 ALT_CON template	AAGAACATGGGCCGTTCCACTAGAGCAGTGGGCCCCGCTCATCTGCCC TCTCTCCCTCCAGATTGGCAGGACGACCAGTC CGACAACCAGTCCGATTACTCAGTGGCTTCAGAGGAAGGTGATGAAAA GAA
CHD4 CON_CON FOR (human)	AGCAGCAGCAAGAAGATCTAGC
CHD4 CON_CON REV (human)	TGCCAATCTCGGTCCTCCTG
CHD4 CON_CON template	AAGAAAGCAGCAGCAAGAAGATCTAGCCCGAAATCTGGGCAAAGGAAA AAGAATCCGTAAACAGGTCAACTACAATGATG GCTCCCAGGAGGACCGAGATTGGCAAAGAA
CHD4_TOTAL FOR (human)	GCAGGTGGCAAAGAAGAAA
CHD4_TOTAL REV (human)	CCACTGGGTAGTAAAAGCAT
SMG1 FOR (human)	CAAGCGATGTCAGCAGATGT
SMG1 REV (human)	TGCTGACAAAAGCCATTCAG
SF3B1 FOR (human)	GTGGGCCTCGATTCTACAGG
SF3B1 REV (human)	GATGTCACGTATCCAGCAAATCT
RBM39 FOR (human)	CAATGCTTGAGGCTCCTTACA
RBM39 REV (human)	TCCGTTCCTTACTTTTGCTTCTC
SF3B4 FOR (human)	CTCCGAGCGGAATCAGGATG
SF3B4 REV (human)	GGCATGTGGGTGTTGACTACT
DHX15 FOR (human)	GGGGACCGATGGGAAGGAT
DHX15 REV (human)	TAGCATTTGTTGAAGCTCGCA
NHP2L1 FOR (human)	CTCGTTCAGCAGTCATGTAACT
NHP2L1 REV (human)	ATGCCCCTGTTGAGGGTTTTG
SRSF1 FOR (human)	CCGCAGGGAACAACGATTG
SRSF1 REV (human)	GCCGTATTTGTAGAACACGTCCT

Supplementary Table 5. AON sequences

SPT5_1	5' - mC*mA*mG*mG*mG*mA*mA*mU*mC*mA*mC*mA*mC*mU*mU*mU*mU*mC*mA*mU*mA*mU*mU*mC*mU*mU*mA*mC*mC*mU*mU*mC*mA*mA*mU - 3'
SPT5_2	5' - mA*mA*mU*mC*mU*mC*mU*mU*mC*mU*mA*mC*mU*mU*mG*mG*mA*mG*mG*mA*mA*mG*mA*mG*mG*mA*mG*mG*mU*mG*mG - 3'
SPT5_3	5' - mU*mU*mU*mC*mC*mC*mA*mG*mG*mU*mC*mA*mA*mC*mA*mA*mC*mU*mG*mG*mG*mG*mU*mA*mU - 3'
NT	5‘ - mC*mC*mU*mU*mC*mC*mC*mU*mG*mA*mA*mG*mG*mU*mU*mC*mC*mU*mC*mC – 3‘
CHD4_1	5' - mG*mU*mU*mC*mA*mU*mC*mA*mA*mA*mG*mU*mC*mU*mU*mC*mA*mU*mC*mA*mC*mC*mU*mU*mC*mC*mU*mC*mU*mG*mA*mA - 3'
CHD4_2	5' - mU*mA*mC*mG*mG*mA*mU*mU*mC*mU*mU*mU*mU*mU*mC*mC*mU*mU*mU*mG*mC*mC*mC*mA*mG*mA*mU - 3'
CHD4_3	5' - mG*mA*mC*mU*mG*mG*mU*mC*mG*mU*mC*mC*mU*mG*mC*mC*mA*mA*mU*mC*mU - 3'

SUPPLEMENTARY SOURCE DATA

Supplementary Source Data 1. Relative to Supplementary Figure 2a

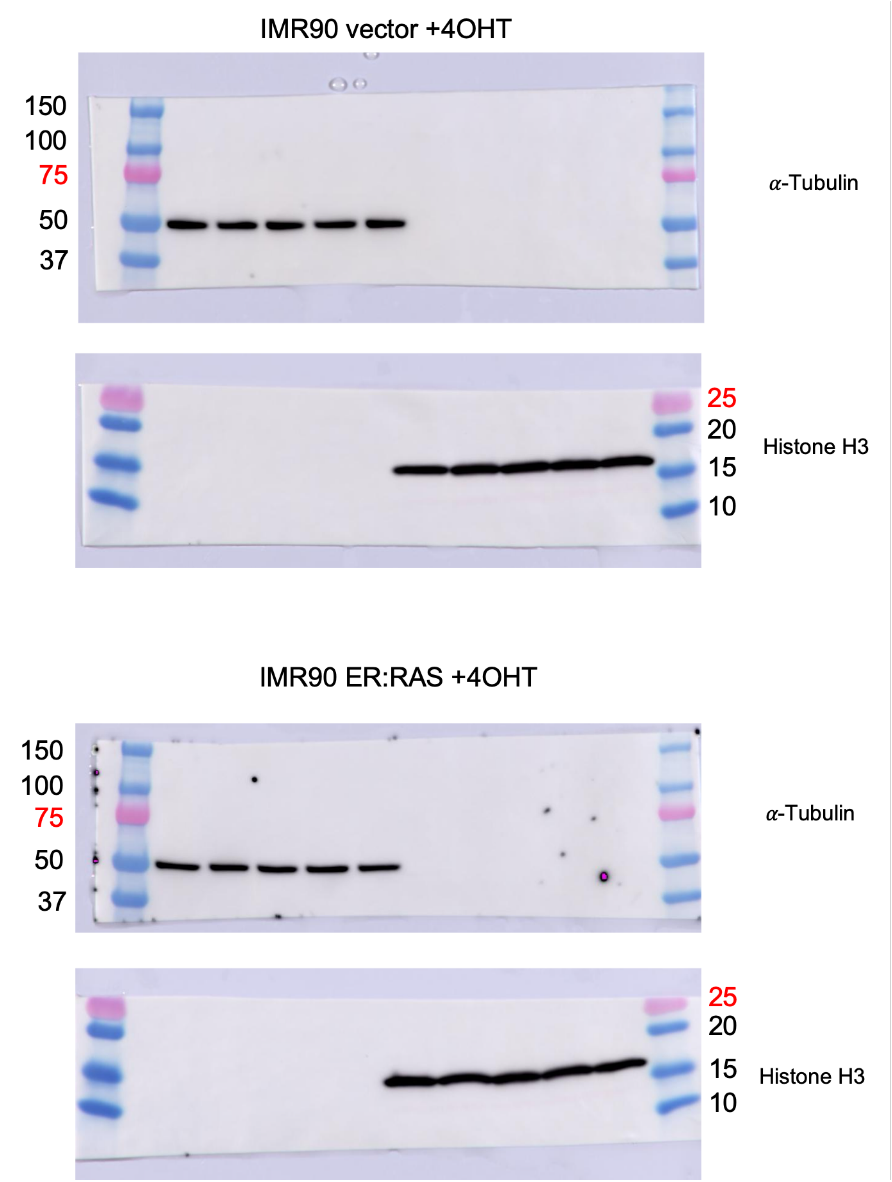

Uncropped images of Western blots shown in Sup. Figure 2a.

The molecular weights (kDa) of size markers are indicated.

Supplementary Source Data 2. Relative to Supplementary Figure 2f

SF3B1

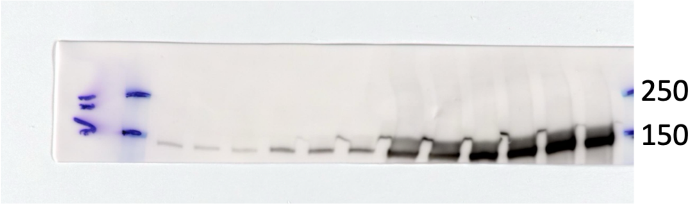

TBP

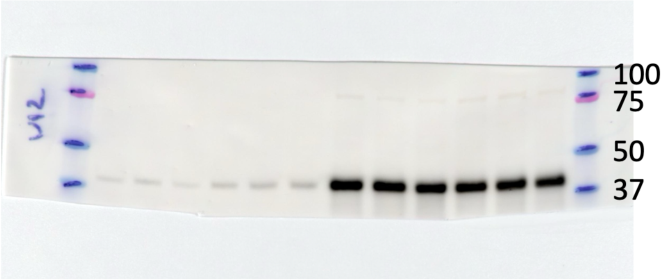

β-Tubulin

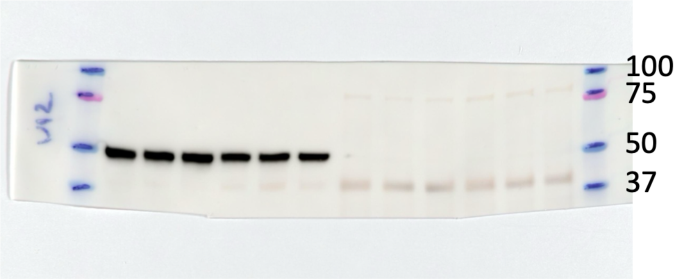

Uncropped images of Western blots shown in Sup. Figure 2f.

The molecular weights (kDa) of size markers are indicated.

Supplementary Source Data 3. Relative to Supplementary Figure 13c and 13f

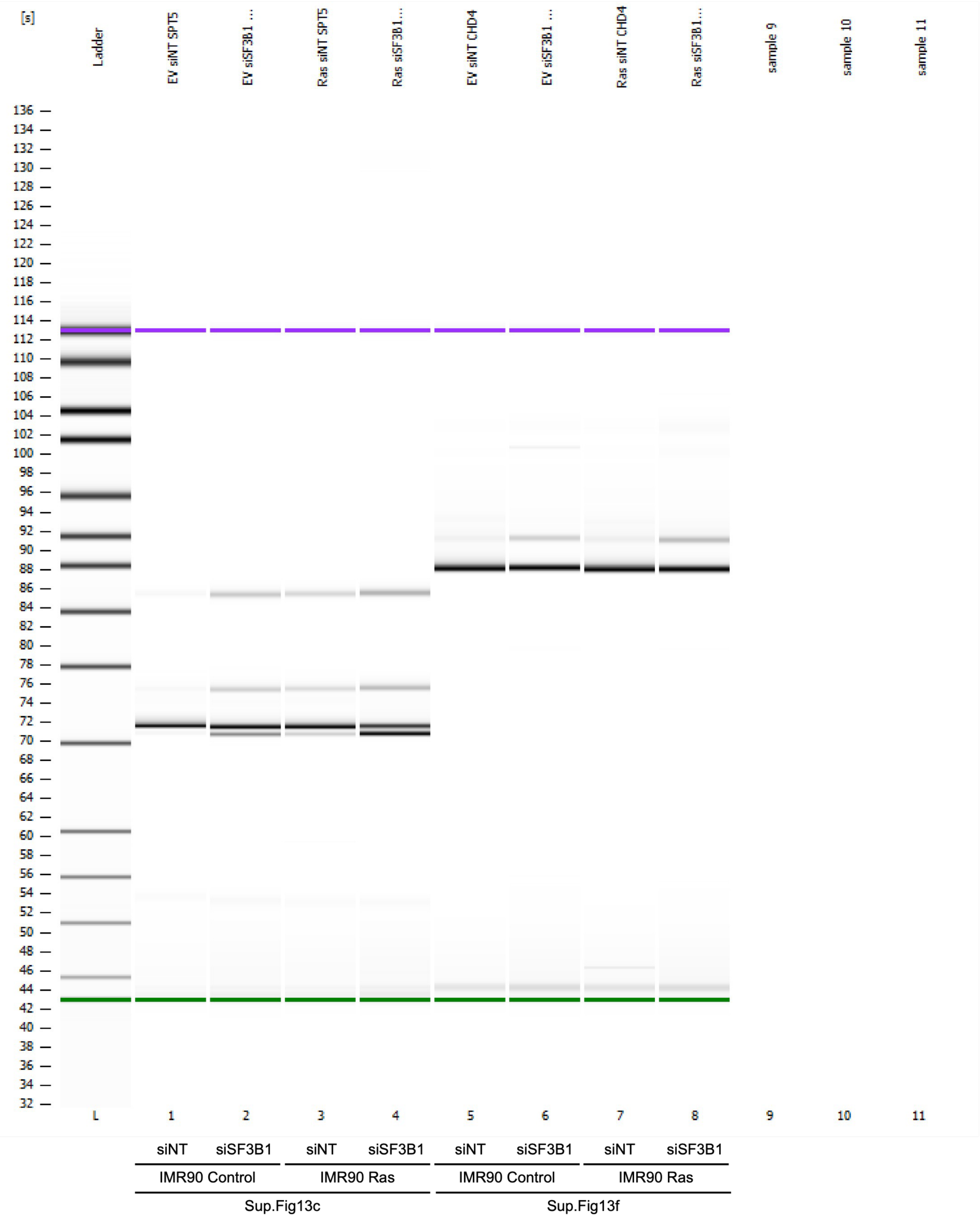

Uncropped image of gels shown in Sup. Figure 13c and 13f.

Retention times (s) are indicated on the left. The first lane is the ladder. There is nothing loaded in lanes 9-11
