# Supplementary material for: Spliceosome induction is a druggable dependency of RAS-driven senescence and cancer
Source: Nat Commun. 2026 Apr 15;17:5208. doi: 10.1038/s41467-026-71564-z (PMC13254117; doi:10.1038/s41467-026-71564-z)
Supplement: Supplementary file 2 — Description of Additional Supplementary Files [file 41467_2026_71564_MOESM2_ESM.pdf]

Title: Supplementary Data 1

Description: Filtered MaxQuant protein group output file MS data.

Title: Supplementary Data 2

Description: siRNA library splicing factors

Title: Supplementary Data 3

Description: siRNA library splicing factors retesting.

Title: Supplementary Data 4

Description: siRNA library downstream effectors

Title: Supplementary Data 5

Description: siRNA library downstream effectors retesting
