# Supplementary material for: Spliceosome induction is a druggable dependency of RAS-driven senescence and cancer
Source: Nat Commun. 2026 Apr 15;17:5208. doi: 10.1038/s41467-026-71564-z (PMC13254117; doi:10.1038/s41467-026-71564-z)
Supplement: Supplementary file 8 — Reporting Summary [file 41467_2026_71564_MOESM8_ESM.pdf]

nature portfolio

Corresponding author(s): Jesus Gil, Verena Wagner

Last updated by author(s): Mar 2, 2026

Reporting Summary

Nature Portfolio wishes to improve the reproducibility of the work that we publish. This form provides structure for consistency and transparency in reporting. For further information on Nature Portfolio policies, see our Editorial Policies and the Editorial Policy Checklist.

Statistics

For all statistical analyses, confirm that the following items are present in the figure legend, table legend, main text, or Methods section.

n/a	Confirmed	
☐	☒	The exact sample size (n) for each experimental group/condition, given as a discrete number and unit of measurement
☐	☒	A statement on whether measurements were taken from distinct samples or whether the same sample was measured repeatedly
☐	☒	The statistical test(s) used AND whether they are one- or two-sided<br>*Only common tests should be described solely by name; describe more complex techniques in the Methods section.*
☒	☐	A description of all covariates tested
☐	☒	A description of any assumptions or corrections, such as tests of normality and adjustment for multiple comparisons
☐	☒	A full description of the statistical parameters including central tendency (e.g. means) or other basic estimates (e.g. regression coefficient) AND variation (e.g. standard deviation) or associated estimates of uncertainty (e.g. confidence intervals)
☐	☒	For null hypothesis testing, the test statistic (e.g. F, t, r) with confidence intervals, effect sizes, degrees of freedom and P value noted<br>*Give P values as exact values whenever suitable.*
☒	☐	For Bayesian analysis, information on the choice of priors and Markov chain Monte Carlo settings
☒	☐	For hierarchical and complex designs, identification of the appropriate level for tests and full reporting of outcomes
☐	☒	Estimates of effect sizes (e.g. Cohen's d, Pearson's r), indicating how they were calculated

Our web collection on statistics for biologists contains articles on many of the points above.

Software and code

Policy information about availability of computer code

Data collection	IIN Cell Analyzer 2000 version 5.2-14311 (64-bit) GE Healthcare<br>IncuCyte Zoom 2018A (2018.1.6628.28170)<br>Microsoft Excel 2016 16.16.13 (190811)

Data analysis

GraphPad Prism Version 9.4.1
IN Cell Investigator 1000 workstation 3.7.2
ImageJ Fiji 2.14.0
GSEA version 2.0.12
DAVID 6.8
Aperio ImageScope (ver. 12.4.0.5043)
MaxQuant v2.4.2.0
FASTQC 0.11.5
Tophat (v.2.0.11)
vast-tools version 2
R limma package, R betAS package, car R package, edgeR package, DESeq2 R package
Cancer Dependency Map (https://depmap.org/portal/)
The files and scripts used for the analysis of the proteomics data are deposited on GitHub (mtinti/spliceosome-senescence) and archived at zenodo (10.5281/zenodo.7858967).

For manuscripts utilizing custom algorithms or software that are central to the research but not yet described in published literature, software must be made available to editors and reviewers. We strongly encourage code deposition in a community repository (e.g. GitHub). See the Nature Portfolio guidelines for submitting code & software for further information.

Data

Policy information about availability of data

All manuscripts must include a data availability statement. This statement should provide the following information, where applicable:

- Accession codes, unique identifiers, or web links for publicly available datasets
- A description of any restrictions on data availability
- For clinical datasets or third party data, please ensure that the statement adheres to our policy

Source data are provided with this paper. RNA-seq data generated in this study have been deposited in the GEO database under accession codes GSE209624, GSE162175, and GSE297268. Mass spectrometry data are deposited in the ProteomeXchange database under accession code PXD035299. The remaining data are available within the Article, Supplementary Information or Source Data file.

Research involving human participants, their data, or biological material

Policy information about studies with human participants or human data. See also policy information about sex, gender (identity/presentation), and sexual orientation and race, ethnicity and racism.

Reporting on sex and gender	Human biopsies were fully anonymised therefore no regard on sex and gender was considered.
Reporting on race, ethnicity, or other socially relevant groupings	Human biopsies were fully anonymised therefore no regard on race, ethnicity was considered.
Population characteristics	Patients who underwent pancreatic resection
Recruitment	Patients provided prior written informed consent
Ethics oversight	Pancreatic cancer tissue specimens were obtained from patients, who underwent pancreatic resection at the y Department of Surgery at Heidelberg University Hospital and provided prior written informed consent under a research protocol approved by the Medical Ethics Committee of Heidelberg University (301/2001 and 159/2002, amendment of May 8th 2012).

Note that full information on the approval of the study protocol must also be provided in the manuscript.

Field-specific reporting

Please select the one below that is the best fit for your research. If you are not sure, read the appropriate sections before making your selection.

☒ Life sciences ☐ Behavioural & social sciences ☐ Ecological, evolutionary & environmental sciences

For a reference copy of the document with all sections, see nature.com/documents/nr-reporting-summary-flat.pdf

Life sciences study design

All studies must disclose on these points even when the disclosure is negative.

Sample size	Sample size was not predetermined. Generally accepted samples sizes were used with reproducible differences between conditions indicating that sample size was sufficient.
Data exclusions	No data was excluded.
Replication	Results of all experiments were reproducible. Numbers of replicates, or numbers of clinical samples, or numbers of animals are indicated for

	each experiment.
Randomization	For animal experiments, littermates were randomly allocated to treatment groups. Tissue culture experiments were not randomized.
Blinding	Investigators were not blinded except for analysis of histological sections.

Reporting for specific materials, systems and methods

We require information from authors about some types of materials, experimental systems and methods used in many studies. Here, indicate whether each material, system or method listed is relevant to your study. If you are not sure if a list item applies to your research, read the appropriate section before selecting a response.

Materials & experimental systems

n/a	Involved in the study
☐	☒ Antibodies
☐	☒ Eukaryotic cell lines
☒	☐ Palaeontology and archaeology
☐	☒ Animals and other organisms
☒	☐ Clinical data
☒	☐ Dual use research of concern
☒	☐ Plants

Methods

n/a	Involved in the study
☒	☐ ChIP-seq
☒	☐ Flow cytometry
☒	☐ MRI-based neuroimaging

Antibodies

Antibodies used	The following antibodies were used in this study: Rabbit polyclonal anti-SF3B1 (Thermo Fisher Scientific, PA5-19679, lot: VL3135364) 1:500-1:1000; Rabbit monoclonal [EPR11986] anti-SF3B1 (Abcam ab172634, lot: 1003548) 1:1000-1:5000; Rabbit polyclonal anti-HCC1 (RBM39) (Thermo Fisher Scientific, PA5-51606, lot: 000018860), 1:100-1:200; Rabbit monoclonal [2D2C8] anti-HCC1 (RBM39) (ProteinTech, 67420-1-Ig, lot:10022276) 1:1000; Rabbit polyclonal anti-HCC1 (RBM39) (Abcam, ab244254, lot 1037608-1) 1:2000; Rabbit polyclonal anti-SF3B4 (Proteintech, 10482-1-AP, lot: 00053884) 1:200; Rabbit polyclonal anti-DHX15 (Atlas Antibodies, HPA047047, lot: 000043569) 1:50-1:200; Rabbit polyclonal anti-PUF60 (Thermo Fisher Scientific, PA5-21411, lot: VA2931357) 1:200; Rabbit polyclonal anti-CWC22 (Thermo Fisher Scientific, PA5-57796, lot: A96309) 1:200; Mouse monoclonal anti-SRSF1 (clone: 103) (Thermo Fisher Scientific, 32-4600, lot: TJ275392) 1:100; Rabbit polyclonal anti-NHP2L1 (Thermo Fisher Scientific, PA5-22010, lot: TH2624110) 1:500; Rabbit polyclonal anti-RNA polymerase II CTD repeat YSPTSPS (Abcam, ab26721, lot: GR3305785-3) 1:1000; Rabbit polyclonal anti-RNA polymerase II CTD repeat YSPTSPS (phospho S2) (Abcam, ab5095, lot: GR3386086-1) 1:1000; Recombinant rabbit monoclonal [EPR19015] anti-RNA polymerase II CTD repeat YSPTSPS (phospho S5) (Abcam, ab193467, lot: GR243496-3) 1:250-1:500; Rat monoclonal [3H9] anti-GFP (Chromotek, 029762, lot: 80626001AB) 1:200; Recombinant rabbit monoclonal [EPR5145(2)] anti-SPT5 (Abcam, ab126592, lot: GR155828-1) 1:200; Mouse monoclonal [3F2/4] anti-CHD4 (Abcam, ab264521, lot: GR3269483-1) 1:200; Recombinant rabbit monoclonal [EPR14104] anti-GFP (Abcam, ab183734, lot: GR298298-24) 1:200; Rabbit polyclonal anti-Histone H3 (Abcam, ab1791, lot: GR65697-1) 1:200; Mouse monoclonal (DM1A) anti-α-Tubulin (Cell Signaling, 3873, lot: 15) 1:800; Rabbit polyclonal anti-SPT5 (Thermo Fisher Scientific, PA5-56100, lot: 000012280) 1:25-1:50; Rabbit monoclonal [EPR21954] anti-TATA binding protein (TBP) (Abcam, ab220788, lot: GR3357208-3) 1:1000; Rabbit monoclonal [D2N5G] anti-β-Tubulin (Cell Signaling Technology 15115, lot: 3) 1:1000; Mouse monoclonal (9A3) anti-DYKDDDDK Tag (Cell Signaling Technology 8146, lot: 5) 1:800; Goat anti-mouse IgG (H+L), Alexa Fluor® 488, conjugated (Thermo Fisher Scientific, A-11029) 1:2000; Goat anti-mouse IgG (H+L), Alexa Fluor® 594, conjugated (Thermo Fisher Scientific, A-11032) 1:2000; Goat anti-rat IgG (H+L), Alexa Fluor® 488, conjugated (Thermo Fisher Scientific, A-11006) 1:2000; Goat anti-rabbit IgG (H+L), Alexa Fluor® 594, conjugated (Thermo Fisher Scientific, A-11037) 1:2000; Goat anti-Rat IgG (H+L) Secondary Antibody, Biotin (Thermo Fisher Scientific, A18869) 1:200; DyLight® 488 Streptavidin (Vector Laboratories, SA-5488-1) 1:200.
Validation	All antibodies against splicing factors and anti-CHD4 and anti-SPT5 have been validated by siRNA-mediated knockdown

Eukaryotic cell lines

Policy information about cell lines and Sex and Gender in Research

Cell line source(s)	IMR90 (CCL-186) and BJ (CRL-2522) cells were purchased from ATCC. MC38 cells were a gift.
Authentication	IMR90 cells were authenticated by DNA (STR) profile performed by Eurofins. BJ and MC38 cells were not authenticated.
Mycoplasma contamination	All cell lines were routinely tested to rule out mycoplasma contamination.
Commonly misidentified lines (See ICLAC register)	IMR90, MC38 and BJ cells are not among commonly misidentified cell lines.

Animals and other research organisms

Policy information about studies involving animals; ARRIVE guidelines recommended for reporting animal research, and Sex and Gender in Research

Laboratory animals	4-8-week-old female C57BL/6, or NSG® mice were purchased from Charles River UK, Ltd.<br>LSL-KrasG12D and Rosa26loxP-STOP-loxP-YFP/+ male and femelle mice have been previously described.<br>8-week-old male C57BL/6 mice were acquired from Janvier Labs.<br>MC38 CRC liver metastasis mouse model as well as the KPC PDAC mouse model have been described previously.
Wild animals	n/a
Reporting on sex	Sex of animals is indicated for each experiment.
Field-collected samples	n/a
Ethics oversight	LThis research complied with all relevant ethical regulations and was approved and overseen by the following ethics review boards. Pancreatic cancer tissue specimens were obtained from patients who underwent pancreatic resection at the Department of Surgery at Heidelberg University Hospital and provided prior written informed consent under a research protocol approved by the Medical Ethics Committee of Heidelberg University (301/2001 and 159/2002, amendment of May 8th, 2012). Liver tumorigenesis experiments and MC38 liver metastasis experiments were performed following German law and with approval from the Regierungspräsidium Karlsruhe (G139/19 and G221/19). All other mouse procedures were performed under license, according to the UK Home Office Animals (Scientific Procedures) Act 1986, ARRIVE 2.0., and local institutional guidelines. The lung cancer experiments were approved by the UCL ethical review committee. Liver cancer initiation experiments were approved by the animal welfare and ethical review board at Imperial College London. Pancreatic cancer experiments were approved by the University of Glasgow's animal welfare and ethical review board.

Note that full information on the approval of the study protocol must also be provided in the manuscript.
