# Supplementary material for: Spliceosome induction is a druggable dependency of RAS-driven senescence and cancer
Source: Nat Commun. 2026 Apr 15;17:5208. doi: 10.1038/s41467-026-71564-z (PMC13254117; doi:10.1038/s41467-026-71564-z)
Supplement: Supplementary file 9 — Transparent Peer Review file [file 41467_2026_71564_MOESM9_ESM.pdf]

nature portfolio

Peer Review File

Spliceosome induction is a druggable dependency of RAS-driven senescence and cancer

Corresponding Author: Professor Jesus Gil

This manuscript has been previously reviewed at another journal. This document only contains information relating to versions considered at Nature Communications.

This file contains all reviewer reports in order by version, followed by all author rebuttals in order by version.

Version 0:

Reviewer comments:

Reviewer #1

(Remarks to the Author)
The authors have properly addressed all issues raised, therefore I recommend publication.

Reviewer #2

(Remarks to the Author)
We have now reviewed the revised manuscript entitled "Spliceosome induction is a druggable dependency of RAS-driven senescence and cancer", which we previously reviewed for another Nature journal. The authors have made changes to the text and included additional experiments and analyses in their revised manuscript to address the comments of the reviewers.
Additional experiments added:
• They have included data on an additional cell line, BJ ER:RAS cells to confirm upregulation of splicing factors levels in response to RAS expression, in support of what they found in IMR90 ER: RAS (Supplementary Fig. 3). They also include new data using BJ ER:RAS cells to confirm sensitivity of these cells to inhibition of SF3B1 and RBM39, and not in other cells (Supplementary Fig. 17d-f and 18a-c).

• They have included data in Supplementary Fig. 6 to address how RAS induction increases splicing factors. They show that multiple inhibitors of mTOR (Rapamycin, Temsirolimus and Torin 1) prevented the induction of SF3B1 in response to oncogenic RAS expression. while inhibition of p38MAPK or S6K did not.

• In response to the reviewers' comment about the use of quantitative immunofluorescence of nuclear staining to measure increased levels of splicing factors - "A validation of these results by Western blot would strengthen the conclusions. For tissue/tumor sections it is understandable why immunofluorescence is preferred, but for cells in culture, a protein-based assay, such as Western blot analysis, would be appropriate since antibodies are available for most of these factors."

The authors supply additional data in a figure for reviewers only (Figure R1) in which they tested monoclonal antibodies against RBM39 and SF3B1 and show that they are specific and show similar induction of splicing factors as observed with the polyclonal antibodies. They added only one Western blot (Supplementary Fig. 2f).

• They have performed new RNA-seq experiment (Supplementary Fig. 11 and 12) to confirm that SF3B1 and RBM39 knockdowns affect the alternative splicing of different subsets of genes. They have also provided experiments in Supplementary Fig. 15 showing that NMD (SMG1 inhibition) is, at least partially, involved in the downregulation of SPT5 and CHD4.

• They have included a new figure (Fig. 8) showing that the RBM39 degrader indisulam targets oncogenic RAS-driven preneoplastic lesions. This is to complement the experiments on inhibition of SF3B1.

• The authors have now included schemes (Supplementary Fig. 13a and c) to explain the strategy to validate splicing of SPT5 and CHD4.

• The authors provide new data to show how AONs targeting CHD4 splicing affect the survival of RAS-expressing cells (Supplementary Fig. 14c-e).

Taken together, the authors have made an effort to address all the reviewers' comments by including new data and analyses and clarifying points in the text. These additions have improved the manuscript significantly.

Open Access This Peer Review File is licensed under a Creative Commons Attribution 4.0 International License, which permits use, sharing, adaptation, distribution and reproduction in any medium or format, as long as you give appropriate credit to the original author(s) and the source, provide a link to the Creative Commons license, and indicate if changes were made.
In cases where reviewers are anonymous, credit should be given to 'Anonymous Referee' and the source.
The images or other third party material in this Peer Review File are included in the article's Creative Commons license, unless indicated otherwise in a credit line to the material. If material is not included in the article's Creative Commons license and your intended use is not permitted by statutory regulation or exceeds the permitted use, you will need to obtain permission directly from the copyright holder.
To view a copy of this license, visit https://creativecommons.org/licenses/by/4.0/

Reviewer #1: senescence, cancer.

In the current work, the authors aim to identify new drugable targets for the treatment of RAS-driven precancerous lesions and cancers. Initially, by employing the IMR90 ER: RAS cellular system, they demonstrate that multiple spliceosome components are upregulated in cells expressing oncogenic RAS. As shown, this up-regulation was: 1. linked to RAS-induced senescence rather than to senescence per se, 2. not related to cell cycle arrest, as it was absent in quiescent cells and interestingly obvious at a stage where cells hyperproliferate and 3. independent of the type of Ras mutation. According to the authors, the high levels of the splicing machinery were also evident in RAS-driven premalignant and malignant in vivo settings. By means of siRNA screening, they found a panel of six spliceosome components, including SF3B1 and RBM39, that turned out imperative for cells expressing oncogenic RAS. They focused on SF3B1 and, by coupling transcriptome and splicing analyses with functional screens, they provide data supporting the importance of SF3B1 in preserving the splicing fidelity in the oncogenic Ras environment. The RNA Pol II-associated factor SPT5 was identified as a potent mediator of the SF3B1 function. Based on the above findings, SF3B1 was inhibited in a liver cancer experimental model, resulting in alleviation of Ras-driven preneoplastic lesions and aggressive tumours. Cumulatively, the authors support the spliceosome as a promising target for RAS-driven cancers.

General Comment

The manuscript is well organised and written, and the authors have performed a detailed analysis using multiple experimental approaches to gain insight into identifying spliceosome components as potential targets to treat RAS-driven lesions. However, in the context of current literature on this topic, the level of novel insight provided in this work falls conceptually short of what would be required for publication in Nature Cell Biology. Particularly, the concept supporting the spliceosome as an essential addiction and vulnerability of Ras-expressing cells has been previously demonstrated in various tumours such as pancreatic and lung cancer, glioblastomas and others (for instance, 10.1186/s13046-021-02153-9; 10.1074/jbc.M116.762757; 10.1186/s13046-022-02241-4). Characteristically, in the work by Alors-Perez et al, upregulated levels of SF3B1 were identified in human Pancreatic Ductal carcinomas and closely associated with distinct splicing event profiles and expression of key PDAC players, including RAS (10.1186/s13046-021-02153-9). In the same work, inhibition of SF3B1 in PDAC cells, exerting RAS signalling, resulted in increased apoptosis and decreased multiple tumour-related features, suggesting SF3B1 targeting as an effective anti-cancer strategy (10.1186/s13046-021-02153-9).
While the reviewer mentions that connections between RAS and some splicing factors have been previously drawn (and they are right), we still believe that many of the aspects of our story, which have been strengthened in our revision, are unique. We take the referenced papers as additional proof of the potential clinical significance of our study.
In particular, our study shows how the upregulation of splicing is a global phenomenon and a broad dependency of RAS-bearing tumours (while the above reference linked it only to specific proteins). We believe that the additional data included in the revised version helps to explain, in part, the mechanism behind this is post-transcriptional (mTOR-mediated translational control). Moreover, our paper has taken advantage of genetic screening to identify multiple splicing vulnerabilities (including SF3B1 and RBM9, but also CWC22, SRSF1, XAB2 and PUF60). In the case of SF3B1, we have also identified novel downstream targets (such as SPT5) that might be both biomarkers and further vulnerabilities. In addition, the novel data added and analysed at the splicing level show the effects of both RBM39 and SF3B1 in this dependency. Finally, we presented 4 sets of experiments in 2 different animal models (of liver cancer initiation and full-blown malignancies), showing that targeting SF3B1 or RBM39 is a vulnerability or oncogenic RAS-driven preneoplasias or full-blown tumours. Overall, we are confident that our study extends and complements the existing literature and includes enough elements of novelty to be considered of interest by the Editors and the broader community.

To acknowledge the reviewer's comment, we have incorporated the following sentence in the introduction:
'Splicing has been suggested to be a vulnerability of different tumour types, including pancreatic cancer (Alors-Perez, Blazquez-Encinas et al. 2021), lung cancer (Fraile, Manchado et al. 2017) and glioblastoma (Fuentes-Fayos, Perez-Gomez et al. 2022). While there is some evidence that splicing might also be a vulnerability of Ras-expressing cells (Fraile, Manchado et al. 2017, Alors-Perez, Blazquez-Encinas et al. 2021), the rationale and specific vulnerabilities have not been systematically explored.'

Specific comments:
1. Complex aspects such as the Ras-signalling cascade and how it can be effectively targeted for anticancer therapy (by targeting spliceosome components in the current study) need to be examined in a broad and multi-level manner to draw firm conclusions and to avoid context-specific phenomena. Thus, functional analysis performed in the current study in the IMR90 ER: RAS cellular system should be expanded by employing additional cellular systems and different oncogenic insults (other than MYC), to verify whether the findings are indeed specific to RAS.

As suggested by the reviewer, we have expanded our previous analysis to other cell systems and analysed additional oncogenic events.

To analyse the induction of splicing factors in response to RAS expression, in addition to the IMR90 ER: RAS model, we have added new data on another cell line, BJ ER: RAS (new Supplementary Fig. 3), confirming the upregulation of splicing factor levels in response to RAS expression. Therefore, we are showing the upregulation of splicing factors in response to Ras expression in 2 cell lines and also in IMR90 ER: RAS E6/E7 cells (e.g. independent of senescence induction). We also observed that Ras-expressing hepatocytes display higher levels of splicing factors both in preneoplastic lesions in the liver (Fig. 3a-c) and in fully-blown malignancies (Fig. 3d). Moreover, Ras-expressing colon metastasis in the liver (Fig. 3e), preneoplastic RAS-expressing epithelial cells in the lung (Fig. 3f- h), and epithelial cells in both mouse and human RAS-driven PDACs (Fig. 3i- l) all display elevated levels of splicing factors.

We have also added new data using BJ ER:RAS, in which we confirmed that inhibition of SF3B1 or RBM39 is a vulnerability of cells expressing oncogenic RAS (new Supplementary Fig. 17d and Supplementary Fig. 18a). Overall, we showed that targeting SF3B1 or RBM39 is a vulnerability associated with RAS signalling in 3 cell lines and in preclinical models of oncogenic RAS-induced liver preneoplasias and full-blown liver tumours. In addition, we took advantage of DepMap to confirm that colon cancer cells expressing mutant *KRAS* are more sensitive to SF3B1 or RBM39 knockdown (but not BUD31or PRMT5 knockdown) than cells expressing wt KRAS (Fig. 4k)

To analyse whether targeting SF3B1 (or RBM39) is a vulnerability associated with oncogenic RAS signaling or can be extended to other oncogenic events, we have included novel experiments that showed that cells expressing an activated version of AKT or in which the expression of the tumour suppressors *TP53* or *CDKN2A* have been knocked down are not more sensitive than their control counterparts to SF3B1 (or RBM39) inhibition (new Supplementary Fig. 17e-f and Supplementary Fig. 18b-c)

Overall, the new (and previous data), show that induction of splicing factors (and the specific associated vulnerabilities described here) is linked with oncogenic RAS signalling (but not with every other oncogenic event) in a variety of cell lines, both in cell culture and *in vivo*.

2. Given that spliceosome component up-regulation was evident even in hyperproliferating RAS-expressing cells prior adoption of the senescence phenotype, contradicting the initial statement that splicing factors are upregulated in nuclei of cells undergoing RAS-induced senescence, it is questionable whether the title accurately reflects the findings in this work.

We have adapted the text to make it clearer that the up-regulation of splicing factors is associated with increased RAS signalling, regardless of the cell fate associated (senescence, hyperproliferation, preneoplasia, neoplasia):

'Altogether, the above results suggest that the induction of the splicing machinery is not a consequence of senescence or the proliferative status of cells but rather is associated with oncogenic RAS signalling and is related, at least in part, to mTOR-mediated translational control.'

3. The expression of a set of spliceosome factors following various manipulations was investigated in the IMR90 ER:RAS cellular model by applying a panel of polyclonal antibodies. To ensure accuracy of the conclusions drawn, the staining results should be further confirmed with at least a second antibody, preferentially a monoclonal one, followed by additional negative control experiments (addition of the corresponding anti-peptide).

To substantiate that the expression of spliceosome factors is induced in response to the expression of oncogenic RAS, we initially have data derived using two different approaches: first, Mass spectroscopy-based proteomics (Fig. 1a-d). This is an unbiased approach that relies on the identification of multiple peptides per protein. In addition, we have also used quantitative immunofluorescence using validated antibodies (e.g., Figure 1f-g). Importantly, the specificity of the antibodies was validated using siRNAs (e.g., Fig. 4e, Fig. 4g, and the second panel from right in Supplementary Fig. 8c, d, e).

As additional controls, we have provided data in Figure R1 only for reviewers, in which we also tested monoclonal antibodies against RBM39 and SF3B1. We show how the signal of other monoclonal antibodies (shown here for antibodies recognising RBM39 and SF3B1) is specific, as the secondary only controls show no signal (Figure R1a, R1c). The monoclonal antibodies against SF3B1 (Figure R1b) and RBM39 (Figure R1d) show a similar induction of the splicing factors on senescence as observed with the polyclonal antibodies. We also validated the specificity of these antibodies using siRNAs, similarly to what we did for the polyclonal antibodies tested in the paper (e.g. Fig. 4e, Fig. 4g and the second panel from right in Supplementary Fig. 8c, d, e). We also demonstrated the specificity of the monoclonal antibodies using siRNAs (Figure R1e, f, g, h). Finally, in the case of SF3B1, we have also conducted Western blots (Supplementary Fig. 2f).

In summary, we have used three different approaches (MS, WB, IF), 2 different antibodies each (for SF3B1 and RBM39), and MS, which is an unbiased approach based on the identification of multiple peptides. We have also provided secondary antibody controls and further validated antibody signals using siRNAs (for all splicing factors tested).

4. In continuation of the previous comment, representative images of lower magnification (depicting more cells per optical field) should be provided for quantitative immunofluorescence of the examined spliceosome factors. Similarly, in Figure 3, images depicting fluorescent signals (b and g) should be complemented by corresponding ones of lower magnification in order to provide a more representative overview of the staining results in the tissues examined.
Following the suggestion of the referee, we have provided representative examples of lower magnification images for the IFs in Fig. 3b, Fig. 3g, Supplementary Fig. 1c, Supplementary Fig. 2d, Supplementary Fig. 3c. It is worth highlighting that IF images are representative examples provided for illustration purposes, but in all cases we have used, as explained in the methods, automated quantification of more than 200 cells per condition.

5. In figures 3i, 3k and 6h, the authors compare staining results in cancerous regions to those observed in areas of the normal exocrine part of the pancreas. This approach is not accurate. The normal counterpart of PDAC lesions is the normal ductal epithelium. Thus, conclusions drawn based on staining outcomes might not be valid. For instance, SF3B1 expression has been previously demonstrated in the epithelium of normal pancreatic ducts ((10.1186/s13046-021-02153-9) and thus staining indices in cancer should be compared with normal baseline levels.
Following the suggestion of the reviewer, we have quantified the normal duct epithelium as a control and provided the new quantifications for the images shown in Figure 3i, 3k, and 6h. While the quantifications vary and are closer in some of the cases (new quantifications shown in Fig. 3j, 3l, and Supplementary Fig. 16j), in all cases they are still significant. We have also provided new images for Figure 3i, 3k, and 6h.

6. According to the results, RBM39 exerted similar to SF3B1 synthetic lethal outcomes when inhibited in Ras-expressing cells, rendering this factor a promising candidate for targeting RAS-driven neoplasias. Thus, extensive functional and in vivo analysis should also include RBM39, given that its inhibition by genetic (silencing) or pharmacological means (for instance, with the aryl sulfonamide drug Indisulam) is feasible and available.
Following the suggestion of the reviewer, we have incorporated a comprehensive set of experiments to analyse the effects of targeting RBM39 using indisulam, a molecular glue that targets RBM39 for degradation.

In the revised manuscript, we have included a new Figure 8 in which we show how indisulam ablates RBM39 expression (Figure 8a), and causes the selective killing of IMR90 cells expressing oncogenic RAS in the context of oncogene-induced senescence (Figure 8b) or RAS-driven hyperproliferation (Figure 8c). This selective killing is due to apoptosis (Figure 8d) and can also be observed upon oncogenic RAS induction in BJ cells (Supplementary Fig. 18a). Importantly, cells with increased expression of AKT or depletion of the tumour suppressors TP53 or CDKN2A are not more sensitive to indisulam than their corresponding controls (Supplementary Fig. 18b-c). Finally, the treatment with indisulam reduces the number of oncogenic RAS-expressing hepatocytes in a model of oncogene-induced senescence in the liver (Fig. 8e-g).

7. More mechanistic insights on apoptosis induction and an increase in Caspase-3/7 activity following SF3B1 inhibition in the RAS-expressing cells should be provided.
We have now added an additional experiment (Supplementary Fig. 17b) in which we use inhibitors of different modalities of cell death (including apoptosis, pyroptosis, ferroptosis, and necroptosis), and we confirm that SF3B1 inhibition results in the selective induction of apoptosis in cells expressing oncogenic RAS.

8. Spliceosome factors play non-canonical functions that support tumorigenesis in all stages. These non-canonical functions are summarised in a recent review (10.1038/s41419-022-05470-9), one of which is chromatin remodelling, which alters the gene expression profile. Since senescence and cancer are characterised by chromatin remodelling, it would be interesting to see how the chromatin landscape changes before and after senescence is induced, and how this plays out in RAS-driven cancer cells following spliceosome factor silencing. Utilisation of chromosome conformation capture methodologies in these settings would provide valuable information in that regard.
This is a very interesting question, but beyond the scope of the current manuscript.

Reviewer #2 cancer, Ras:

In the paper by Wagner et al., the team is assessing the role of upregulated RNA splicing factors in RAS-induced senescence. Currently, there is an interest in understanding the dependencies of mutant RAS-driven cancers to improve therapies. While this paper explores exciting concepts and therapeutic opportunities, it lacks deep mechanistic insights to answer the questions:

1. By which mechanism is RAS inducing increased splicing factors? The authors build a compelling case of splicing factors being upregulated by RAS, independent of senescence; however, the mechanism is missing. While it is suggested that it is post-transcriptional mechanisms, this is not further pursued. Is it by decreased nonsense-mediated decay or decreased protein turnover by oncogenic RAS?
We thank the reviewer for this interesting question. We believe that control of translation is a factor contributing at least in part to the process. To analyse what factors could contribute to the increased expression of splicing factors by oncogenic RAS, we decided to concentrate on SF3B1 expression and analysed the effect of a battery of inhibitors (a selection of which are shown in Supplementary Fig. 6) on the induction of SF3B1 expression in response to oncogenic RAS signalling. While inhibition of p38MAPK or S6K did not, we observed that multiple inhibitors of mTOR (Rapamycin, Temsirolimus and Torin 1) prevented the induction of SF3B1 in response to oncogenic RAS expression. While the mechanism explaining the post-transcriptional induction of multiple splicing factors in response to oncogenic RAS might be more complex, the above experiments suggest that mTOR-driven transcriptional control contributes to the process.

2. Is really the SPT5 a target of SF3B1 and RBM39? And more importantly, is the aberrant splicing of SPT5 leading to NMD and thus the changes in expression? This is assumed in the manuscript but not tested.
In the manuscript, we concentrated on analysing the mechanism associated with SF3B1 inhibition in cells expressing oncogenic RAS. Consequently, we analysed the effect that SF3B1 inhibition has on the expression and splicing of potential effector genes (Fig. 5a-d) and assessed which SF3B1 targets mediate their effects, identifying *SPT5* (i.e., *SUPT5H*) and *CHD4* (Fig. 5e-i). In that context, we expect SPT5 to be a target and mediator of the effects of SF3B1, but not necessarily of RBM39.

To understand whether SPT5 splicing (or expression) is dependent on RBM39, we have performed a new RNA-Seq experiment (presented in the new Supplementary Fig. 11) that reproduced the detection of the alternative splicing events in the aforementioned SF3B1 inhibition experiment (Supplementary Fig. 11c-d). This analysis shows that, while the expression of *CHD4* and splicing of *SPT5* and *CHD4* are controlled by SF3B1, they are not so dependent on RBM39 (Supplementary Fig. 11f-g and new Supplementary Fig. 12b-d). Based on the RNA-Seq analyses and the experimental data, we can conclude that both SPT5 and CHD4 alternative splicing events are targets of SF3B1, a component of the U2 snRNP and therefore a “core” splicing factor.

Regarding whether NMD, apparently upregulated by SF3B1 knockdown, contributes to explaining how the aberrant splicing of *SPT5* (and *CDH4*) relates to its change in expression, we have now added new data (presented in new Supplementary Fig. 15), in which we knock down *SMG1*, a gene involved in NMD. As a control, we showed that *SMG1* knockdown inhibits nonsense-mediated mRNA decay (NMD) by using a vector (HBB PTC39) that expresses a Beta-globin-PTC39 NMD reporter. Knocking down *SMG1* results in higher expression of the reporter (as detected by IF, Supplementary Fig. 15a). Partial inhibition of NMD by knockdown of *SMG1* also resulted in increased levels of SPT5 (Supplementary Fig. 15e) and CHD4 (Supplementary Fig. 15f). Those results suggest that NMD contributed to explaining, at least in part, the observed effects. However, the alternative splicing events of interest are unlikely to be the culprits, as they are frame-preserving. With the drop in splicing efficiency induced by knocking down *SF3B1*, several frame-disrupting introns likely get retained in both genes, triggering NMD. Such events, like poison exons, would not be easily detected by RNA-seq because transcripts are polyA-selected and therefore sensitivity for NMD target molecules is very low. Therefore, we have opted to discuss this carefully in the text.

Other detailed points include:

Given that SF3B1 is an essential gene, and it is upregulated in the senescent cells, how did the authors control for this? The knockdown efficiency in Supp 6a is presented as a fold change within cell line, but how is the knockdown efficiency across models? That can rule out whether differences in the screen are attributed to this efficiency and baseline levels of the proteins. Presenting the western blots, rather than qRT-PCR, can help.
We have previously conducted controls to assess that, in principle, the silencing efficiency of siRNAs across senescence and non-senescence conditions is equal by knocking down GFP in both senescent and non-senescent conditions(e.g. we presented such controls in McHugh et al Nat Cell Bio, 2023; Sup Fig. 1b). The knockdown efficiencies for the genes selected from the screen have been presented (e.g. in the case of SF3B1, Supplementary Fig. 8a) as mRNA. But we also presented it as a quantitative IF analysis (Fig. 4e) and compared both conditions

in the same graph. The data based on quantitative assessment of IF for SF3B1, RBM39, CW22, SRSF1 and PUF60 is presented in Fig. 4e, 4g, and the second panel from right of Supplementary Fig. 8c, d and e.

The paper starts from a senescence standpoint, but intro and abstract lack connecting senescence to therapy resistance. First 3 figures are devoted to describing the upregulation of splicing factors by oncogenic KRAS but not senescence, however, mechanism is lacking.
As explained in point 1 to Reviewer 2, control of translation is a factor contributing at least in part to the process. To analyse what factors could contribute to the increased expression of splicing factors by oncogenic RAS, we decided to concentrate on SF3B1 expression and analysed the effect of a battery of inhibitors (a selection of which are shown in Supplementary Fig. 6) on the induction of SF3B1 expression in response to oncogenic RAS signalling. While inhibition of p38MAPK or S6K did not, we observed that multiple inhibitors of mTOR (Rapamycin, Temsirolimus and Torin 1) prevented the induction of SF3B1 in response to oncogenic RAS expression.

While the mechanism explaining the post-transcriptional induction of multiple splicing factors in response to oncogenic RAS might be more complex, the above experiments suggest that mTOR-driven transcriptional control is a contributor to the process.

Also, if senescence it not what triggers the upregulation of splicing factors and STP5, then maybe de-emphasizing senescence from the manuscript may help and make an easier connection to therapeutic vulnerability in RAS driven tumours.
We realise that the narrative of connecting our initial analysis of senescence and the fact that splicing is rather a vulnerability of RAS-driven tumours needs improving for clarity. We appreciate this suggestion, and we have tried to make this clearer in the revamped version of the manuscript:

‘Altogether, the above results suggest that the induction of the splicing machinery is not a consequence of senescence or the proliferative status of cells but rather is associated with oncogenic RAS signalling and is related, at least in part, to mTOR-mediated translational control.’

There is a missed opportunity to further analyze the RNASeq data on Figure 5A at the splicing level. Are there common nucleotide motifs in exons and introns impacted by SF3B1? How is exactly STP5 misspliced by SF3B1? Can the authors show a sashimi plot? Is RBM39 also regulating the splicing of SPT5?
SF3B1 is a component of the U2 snRNP (which specifically binds to the branch point of introns) and therefore a “core” splicing factor. Indeed, we can observe that its ablation causes widespread changes in alternative splicing, suggesting reduced splicing efficiency: more intron retention and more exon skipping (Fig. 5b, c and Supplementary Fig. 10).

This manner of splicing regulation appears to be maintained during RAS-induced senescence. As visible in RNA Maps derived from ENCODE SF3B1 CLIP data, its binding distribution across exons and introns in proliferative and senescent cells exhibits a peak around the expected location of the branch points of introns, near their 3´ end (Figure R2). Moreover, that signal is stronger upstream of constitutive exons.

We have now added additional information in Supplementary Fig. 12c-d, showing sashimi plots for both SPT5 and CHD4. As explained in response to main question 2 of this reviewer, SPT5 is regulated by SF3B1, not by RBM39. We have included the relevant information in Supplementary Fig. 12b.

Can the authors comment on what were the most upregulated hits in the mass spec from 1C? While they provide a rationale for why focusing on splicing factors, not insight is given for the other significant hits.
We have submitted the full dataset to PRIDE. We have added a brief sentence on the most upregulated hits observed in Fig. 1C:

‘Among the bona fide nuclear proteins more highly upregulated in senescent cells, we identified transcription factors such as RELA (known to be induced on senescence (Chien, Scuoppo et al. 2011)) or BHLHE40 (Supplementary Table S1).’

Reviewer #3 cancer, splicing:

The manuscript by Wagner et al. describes a proteomic analysis of IMR90 cells expressing a chimeric fusion between oncogenic HRAS and estrogen receptor ligand binding domain, which can be induced by treatment with 4OHT, causing the cells to undergo RAS-induced senescence. They uncovered upregulation of multiple splicing factors during RAS-induced senescence. They go on to show that this increase is specifically driven by RAS signaling, and not a general effect due to senescence. They confirmed this correlation using multiple in vivo mouse models for both tumor initiation and aggressive liver cancer driven by RAS, xenografts derived from MC38 KRAS mutant colorectal cancer cells, lung lesions in KRAS model of lung cancer, KRAS P53 PDAC model and tissue sections from PDAC patients. Using a siRNA library targeting 189 splicing factors they identified 18 splicing factors whose knockdown selectively killed RAS-expressing cells, with SF3B1 and RBM39 showing the strongest effect. Focusing on SF3B1 they analyzed global effect of SF3B1 knockdown on splicing in RAS-expressing cells using RNA-seq. They identified 132 genes that were differentially spliced and showed decreased overall expression in SF3B1 knockdown cells. They confirmed that two of these genes, SPT5 and CHD4, are essential in RAS-expressing cells. Targeting SPT5 with antisense oligonucleotides reduced the viability of oncogenic RAS-expressing cells. Finally, using two liver cancer mouse models, they showed that treatment with SF3B1 inhibitors, E7107 or PlaB, resulted in reduction of RAS positive hepatocytes or significant reduction in tumor numbers. The authors suggest that SF3B1 inhibitors could be effective therapeutics for the treatment of RAS-driven tumors.

The findings of this manuscript are very relevant to cancers that have activated or mutated RAS. New approaches to target RAS-driven tumors are important for developing alternative cancer treatments. This manuscript describes such an approach, identifying spliceosome components as targets of RAS-specific vulnerabilities in RAS-expressing cells/tumors. The manuscript is well written, and the figures clearly presented. The authors do a good job of making the connection between RAS signaling and upregulation of spliceosome components, using multiple cell lines, conditions, inhibitors and model systems. However, the experiments describing the splicing targets are limited to a single splicing factor, SF3B1, and a single splicing target, SPT5. More experiments are needed for the results to be generalized.

Specific comments

1. The authors should state what is the biological source of IMR90 cells.
Human lung (female) fibroblasts, this has been specified in the materials and methods.

2. In the discussion the authors write "RAS induces a post-transcriptional accumulation of multiple spliceosome components in the nuclei." The authors offer no explanation or experiments to address how upregulation of the spliceosome components occurs. Is it enhanced production, increased stabilization, decreased degradation etc.?
As explained in point 1 to Reviewer 2, control of translation is a factor contributing at least in part to the process. To analyse what factors could contribute to the increased expression of splicing factors by oncogenic RAS, we decided to concentrate on SF3B1 expression and analysed the effect of a battery of inhibitors (a selection of which are shown in Supplementary Fig. 6) on the induction of SF3B1 expression in response to oncogenic RAS signalling. While inhibition of p38MAPK or S6K did not, we observed that multiple inhibitors of mTOR (Rapamycin, Temsirolimus and Torin 1) prevented the induction of SF3B1 in response to oncogenic RAS expression.

While the mechanism explaining the post-transcriptional induction of multiple splicing factors in response to oncogenic RAS might be more complex, the above experiments suggest that mTOR-driven transcriptional control is a contributor to the process.

3. All the experiments showing increased levels of splicing factors are performed using immunofluorescence of nuclear staining and quantitation (Fig. 1f-g; sup. Fig. 1b-f; Fig. 2a-g; sup. Fig. 2d; sup. Fig. 3b; sup. Fig. 4; Fig. 4c,h; sup. Fig. 6c-e; Fig. 5g-i; Fig. 6; sup. Fig. 10). A validation of these results by Western blot would strengthen the conclusions. For tissue/tumor sections it is understandable why immunofluorescence is preferred, but for cells in culture, a protein-based assay, such as Western blot analysis, would be appropriate since antibodies are available for most of these factors.
As explained in point 3 to Reviewer 1, to substantiate that the expression of spliceosome factors is induced in response to the expression of oncogenic RAS, we initially have data derived using two different approaches: first, Mass spectroscopy-based proteomics (Fig. 1a-d). This is an unbiased approach that relies on the identification of multiple peptides per protein. In addition, we have also used quantitative immunofluorescence using validated antibodies (e.g. Figure 1f-g). Importantly, the specificity of the antibodies was validated using siRNAs (e.g. Fig. 3e, Fig. 3g and the second panel from right in Supplementary Fig. 8c, d, e, f).

As additional controls, we have provided data in Figure R1 only for reviewers, in which we also tested monoclonal antibodies against RBM39 and SF3B1. We show how the signal of other monoclonal antibodies (shown here for antibodies recognising RBM39 and SF3B1) is specific, as the secondary only controls show no signal (Figure R1a). The monoclonal antibodies against SF3B1 (Figure R1b) and RBM39 (Figure R1c) show a similar induction of the splicing factors on senescence as observed with the polyclonal antibodies. We also validated the specificity of these antibodies using siRNAs, similarly to what we did for the polyclonal antibodies tested in the paper (e.g. Fig. 3e, Fig. 3g and the second panel from right in Supplementary Fig. 8c, d, e, f). We have also demonstrated the specificity of the monoclonal antibodies using siRNAs (Figure R1d,e). Finally, in the case of SF3B1, we have also conducted Western blots (Supplementary Fig. 2f).
In summary, we have used three different approaches (MS, WB, IF), 2 different antibodies (each for SF3B1 and RBM39), and MS, which is an unbiased approach based on the identification of multiple peptides. We have provided secondary antibody controls and validated antibody signals using siRNAs.

4. Fig. 5 The authors write on the bottom page 9, "Next, we wondered whether any of the transcripts whose splicing was impacted by the loss of SF3B1 would also represent vulnerabilities in oncogenic RAS-expressing cells. We, therefore, looked for transcripts that were both differentially/aberrantly spliced in SF3B1 knockdown cells and showed decreased overall expression." It is not clear why the authors focused on targets with decreased expression if they are looking for transcripts that show splicing changes upon loss of SF3B1. How do they explain the mechanism by which loss of SF3B1 causes decreased expression of the targets?
We focused on that set of transcripts for two reasons: on one hand, one of the potential consequences of aberrant splicing might be the destabilisation of transcripts and reduced expression via NMD or other mechanisms; on the other hand, in practical terms, the effect of this subset of genes is more amenable to being experimentally tested using siRNAs.

As explained in point 2 to reviewer 2, our new data suggest that NMD contributes at least in part to explain it. We have now added some new data (presented in the new Supplementary Fig. 15), in which we knock down SMG1, a gene involved in NMD. As a control, the knockdown of SMG1 inhibits Nonsense-mediated mRNA decay (NMD), so we took advantage of a vector (HBB PTC39) that expresses a Beta-globin-PTC39 NMD reporter. Knocking down SMG1 results in higher expression of the reporter (as detected by IF, Supplementary Fig. 15a). Partial inhibition of NMD by knockdown of SMG1 resulted in increased levels of SPT5 (Supplementary Fig. 15f) and CHD4 (Supplementary Fig. 15e). Those results suggest that NMD contributed to explaining, at least in part, the observed effects. However, the alternative splicing events of interest are unlikely to be the culprits, as they are frame-preserving. With the drop in splicing efficiency induced by knocking down SF3B1, several frame-disrupting introns likely get retained in both genes, triggering NMD. Such events would not be easily detected by RNA-Seq because transcripts are polyA-selected and, therefore sensitivity for NMD target molecules is very low. Therefore, we have opted to discuss this carefully in the text.

5. bottom page 9 The authors write "Taken together the above results show that cells expressing oncogenic RAS require high splicing levels and that it is possible to target specific splicing factors, including RBM39 and SF3B1, to disrupt splicing and reduce the viability of the cells". The authors do not include any further experiments with RBM39 although they mention in the Discussion "Inhibitors for two of these factors, SF3B1 and RBM39, already exist".
As described in point 6 to reviewer 1, we have included a new Figure 8 in which we show how indisulam results in ablation of RBM39 expression (Fig. 8a), and results in the selective killing of IMR90 cells expressing oncogenic RAS in the context of oncogene-induced senescence (Fig. 8b) or RAS-driven hyperproliferation (Fig. 8c). This selective killing is due to apoptosis (Fig. 8d) and can be also observed upon oncogenic RAS induction in BJ cells (Supplementary Fig. 17d). Importantly, cells with increased expression of AKT or depletion of the tumour suppressors TP53 or CDKN2A are not more sensitive to indisulam than their corresponding controls (Supplementary Fig. 17e-f). Finally, the treatment with indisulam reduces the number of oncogenic RAS-expressing hepatocytes in a model of oncogene-induced senescence in the liver (Fig. 8e-g).

6. Sup. Figure 9b-c The authors write "We confirmed that the AONs induced exon skipping and resulted in reduced overall levels of SPT5 mRNA (Sup. Figure 9b-c)." Panel b shows decreased SUPT5H mRNA after treatment with AONs, but it is not clear from panel c how splicing was validated.
We have now included schemes (Supplementary Fig. 13a, c) to show the strategy to validate splicing.

7. Page 10 The authors write "These results are consistent with the idea that aberrant splicing of specific transcripts, such as CHD4 and SPT5, may contribute to the sensitivity of cells expressing oncogenic RAS to splicing inhibition". The manuscript does not include experiments showing that aberrant splicing of CHD4 has an effect on sensitivity of RAS-expressing cells, only knockdown of CHD4. If the authors want to write a generalized statement about aberrant splicing, they need to demonstrate further examples.
We have now included new data in Supplementary Fig. 14c-e to show how AONs targeting CHD4 splicing affect the survival of cells expressing oncogenic RAS.

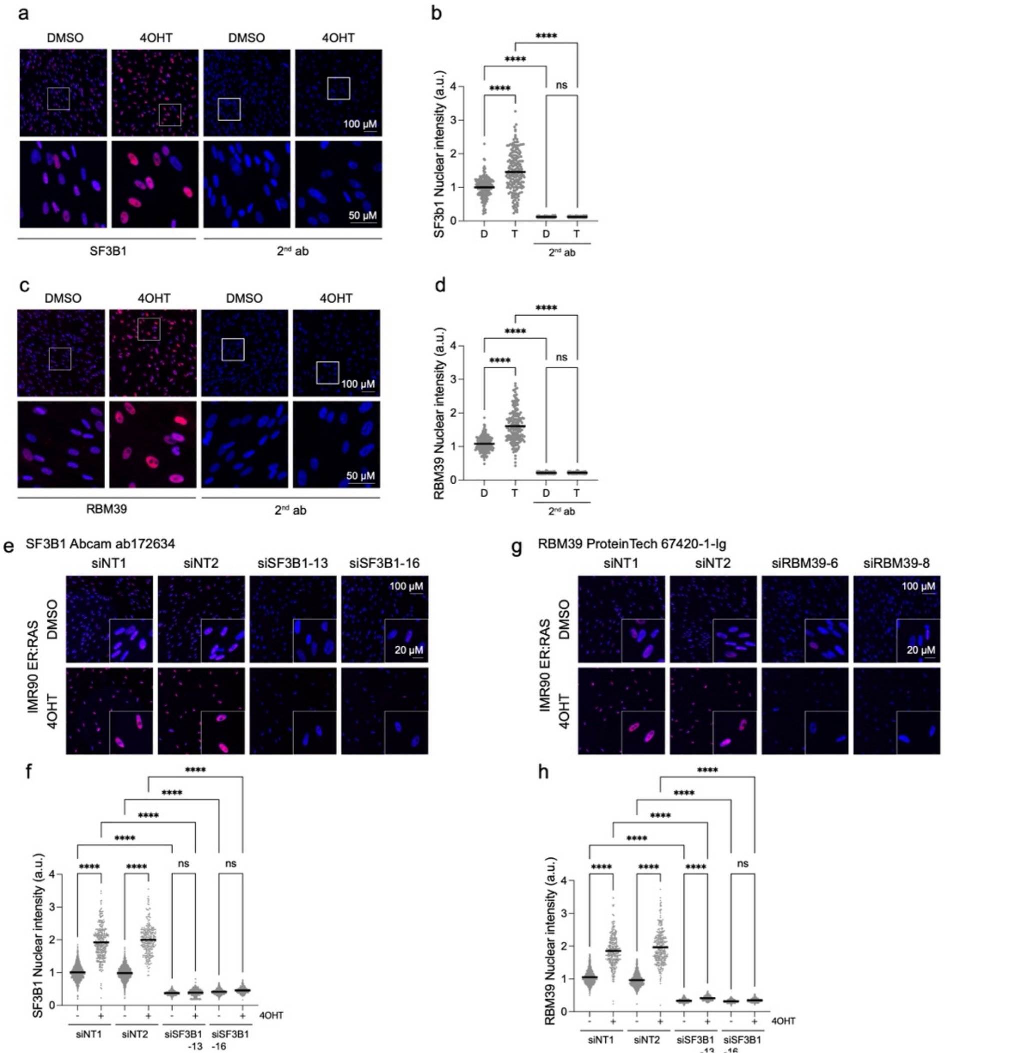

Figure R1 only for reviewers. Additional control to show the specificity of SF3B1 and RBM39 immunofluorescences. **a,** Immunofluorescence staining of SF3B1 in control IMR90 ER:RAS cells (+DMSO) or IMR90 ER:RAS cells undergoing senescence (+4OHT) with and without the primary antibody (Abcam ab172634). Representative images, scale bar 100 µM and 20 µM for the higher magnification. **b,** Quantification of the nuclear intensities. n>200 cells per condition. Ordinary one-way ANOVA with Tukey's multiple comparisons test. **** <0.0001; ns: non-significant. **c,** Immunofluorescence staining of RBM39 in control IMR90 ER:RAS cells (+DMSO) or IMR90 ER:RAS cells undergoing senescence (+4OHT) with and without the primary antibody (ProteinTech 67420-1-Ig). Representative images, scale bar 100 µM and 20 µM for the higher magnification. **d,** Quantification of the nuclear intensities. n>200 cells per condition. Ordinary one-way ANOVA with Tukey's multiple comparisons test. **** <0.0001; ns: non-significant. **e,** Immunofluorescence staining of SF3B1 in control IMR90 ER:RAS cells (+DMSO) or IMR90 ER:RAS cells undergoing senescence (+4OHT) 72h after the transfection with the siRNAs non-targeting (siNT1 and siNT2) or targeting SF3B1 (siSF3B1-13 and siSF3B1-16). Representative images, scale bar 100 µM and 20 µM for the higher magnification. **f,** Quantification of the nuclear intensities. n>200 cells per condition. Ordinary one-way ANOVA with Tukey's multiple comparisons test. **** <0.0001; ns: non-significant. **g,** Immunofluorescence staining of RBM39 in control IMR90 ER:RAS cells (+DMSO) or IMR90 ER:RAS cells undergoing senescence (+4OHT) 72h after the transfection with the siRNAs non-targeting (siNT1 and siNT2) or targeting SF3B1 (siRBM39-6 and siRBM39-8). Representative images, scale bar 100 µM and 20 µM for the higher magnification. Quantification of the nuclear intensities. n>200 cells per condition. Ordinary one-way ANOVA with Tukey's multiple comparisons test. **** <0.0001; ns: non-significant.

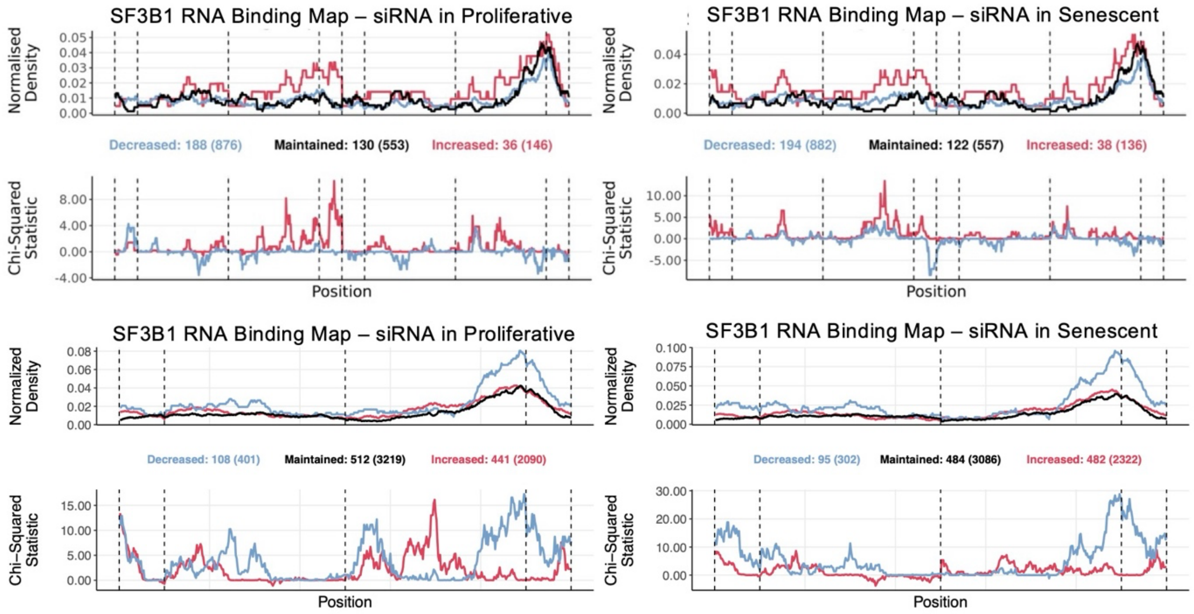

Figure R2 only for reviewers. RNA binding maps of alternative exon inclusion (top) and intron retention (bottom) events in IMR90 cells treated with DMSO (Left) or 4OHT (Right) exposed to siRNA against SF3B1. On the top half of each plot, the normalised binding of SF3B1 to metagenomic regulatory sequences of alternative events is shown. The plot discriminates more excluded (blue), more included (red) or maintained (black) alternative sequences (exons/introns) upon SF3B1 knockdown. The total number of events in each category can be found beneath the plot in brackets, preceded by the number of events for which there is at least one binding site for SF3B1. On the bottom half of each plot, the effect size (Chi-Squared Statistic) of excluded (blue) or included (red) normalised binding, in relation to "maintained" events, is given. For alternative exons (top), vertical lines delimitate the following metagenomic splicing regulatory sequences, in order: last 50 nt of upstream constitutive exon; first 200 nt of upstream intron; last 200 nt of upstream intron; first 50 nt of alternative exon; last 50 nt of alternative exon; first 200 nt of downstream intron; last 200 nt of downstream intron; first 50 nt of downstream constitutive exon. For intron retention events (bottom), vertical lines delimitate the following metagenomic splicing regulatory sequences, in order: last 50 nt of upstream exon; first 200 nt of intron; last 200 nt of intron; first 50 nt of downstream exon.

REFERENCES FOR THIS REBUTTAL

Alors-Perez, E., R. Blazquez-Encinas, S. Alcala, C. Viyuela-Garcia, S. Pedraza-Arevalo, V. Herrero-Aguayo, J. M. Jimenez-Vacas, A. Mafficini, M. E. Sanchez-Frias, M. T. Cano, F. Abollo-Jimenez, J. A. Marin-Sanz, P. Cabezas-Sainz, R. T. Lawlor, C. Luchini, L. Sanchez, J. M. Sanchez-Hidalgo, S. Ventura, L. Martin-Hijano, M. D. Gahete, A. Scarpa, A. Arjona-Sanchez, A. Ibanez-Costa, B. Sainz, Jr., R. M. Luque and J. P. Castano (2021). "Dysregulated splicing factor SF3B1 unveils a dual therapeutic vulnerability to target pancreatic cancer cells and cancer stem cells with an anti-splicing drug." J Exp Clin Cancer Res **40**(1): 382.

Chien, Y., C. Scuoppo, X. Wang, X. Fang, B. Balgley, J. E. Bolden, P. Premsrirut, W. Luo, A. Chicas, C. S. Lee, S. C. Kogan and S. W. Lowe (2011). "Control of the senescence-associated secretory phenotype by NF-{kappa}B promotes senescence and enhances chemosensitivity." Genes & development **25**(20): 2125-2136.

Fraile, J. M., E. Manchado, A. Lujambio, V. Quesada, D. Campos-Iglesias, T. R. Webb, S. W. Lowe, C. Lopez-Otin and J. M. Freije (2017). "USP39 Deubiquitinase Is Essential for KRAS Oncogene-driven Cancer." J Biol Chem **292**(10): 4164-4175.

Fuentes-Fayos, A. C., J. M. Perez-Gomez, G. G. ME, J. M. Jimenez-Vacas, C. Blanco-Acevedo, R. Sanchez-Sanchez, J. Solivera, J. J. Breunig, M. D. Gahete, J. P. Castano and R. M. Luque (2022). "SF3B1 inhibition disrupts malignancy and prolongs survival in glioblastoma patients through BCL2L1 splicing and mTOR/ss-catenin pathways imbalances." J Exp Clin Cancer Res **41**(1): 39.

POINT BY POINT ANSWER TO REVIEWERS' COMMENTS for NCOMMS-24-22649-T

REVIEWER #1

The authors have properly addressed all issues raised, therefore I recommend publication.

We thank the reviewer for their feedback that helped improve the manuscript.

REVIEWER #2

We have now reviewed the revised manuscript entitled "Spliceosome induction is a druggable dependency of RAS-driven senescence and cancer", which we previously reviewed for another Nature journal. The authors have made changes to the text and included additional experiments and analyses in their revised manuscript to address the comments of the reviewers.

Additional experiments added:
• They have included data on an additional cell line, BJ ER:RAS cells to confirm upregulation of splicing factors levels in response to RAS expression, in support of what they found in IMR90 ER: RAS (Supplementary Fig. 3). They also include new data using BJ ER:RAS cells to confirm sensitivity of these cells to inhibition of SF3B1 and RBM39, and not in other cells (Supplementary Fig. 17d-f and 18a-c).

• They have included data in Supplementary Fig. 6 to address how RAS induction increases splicing factors. They show that multiple inhibitors of mTOR (Rapamycin, Temsirolimus and Torin 1) prevented the induction of SF3B1 in response to oncogenic RAS expression. while inhibition of p38MAPK or S6K did not.

• In response to the reviewers' comment about the use of quantitative immunofluorescence of nuclear staining to measure increased levels of splicing factors - "A validation of these results by Western blot would strengthen the conclusions. For tissue/tumor sections it is understandable why immunofluorescence is preferred, but for cells in culture, a protein-based assay, such as Western blot analysis, would be appropriate since antibodies are available for most of these factors."

The authors supply additional data in a figure for reviewers only (Figure R1) in which they tested monoclonal antibodies against RBM39 and SF3B1 and show that they are specific and show similar induction of splicing factors as observed with the polyclonal antibodies. They added only one Western blot (Supplementary Fig. 2f).

• They have performed new RNA-seq experiment (Supplementary Fig. 11 and 12) to confirm that SF3B1 and RBM39 knockdowns affect the alternative splicing of different subsets of genes. They have also provided experiments in Supplementary Fig. 15 showing that NMD (SMG1 inhibition) is, at least partially, involved in the downregulation of SPT5 and CHD4.

• They have included a new figure (Fig. 8) showing that the RBM39 degrader indisulam targets oncogenic RAS-driven preneoplastic lesions. This is to complement the experiments on inhibition of SF3B1.

• The authors have now included schemes (Supplementary Fig. 13a and c) to explain the strategy to validate splicing of SPT5 and CHD4.

• The authors provide new data to show how AONs targeting CHD4 splicing affect the survival of RAS-expressing cells (Supplementary Fig. 14c-e).

Taken together, the authors have made an effort to address all the reviewers' comments by including new data and analyses and clarifying points in the text. These additions have improved the manuscript significantly.

We thank the reviewer for their suggestions that has resulted in new experiments which improved the quality of the manuscript.
